# Branched Linkers for Homogeneous Antibody-Drug Conjugates: How Long Is Long Enough?

**DOI:** 10.3390/ijms252413356

**Published:** 2024-12-12

**Authors:** Evgeny L. Gulyak, Olga A. Komarova, Yury A. Prokopenko, Elina A. Faizullina, Diana M. Malabuiok, Aigul R. Ibragimova, Yuliana A. Mokrushina, Oxana V. Serova, Galina P. Popova, Mikhail Y. Zhitlov, Timofei D. Nikitin, Vladimir A. Brylev, Alexey V. Ustinov, Vera A. Alferova, Vladimir A. Korshun, Ivan V. Smirnov, Stanislav S. Terekhov, Ksenia A. Sapozhnikova

**Affiliations:** 1 Shemyakin-Ovchinnikov Institute of Bioorganic Chemistry, Miklukho-Maklaya 16/10, 117997 Moscow, Russia; evgeny.gulyak@gmail.com (E.L.G.); okomarova802@gmail.com (O.A.K.); tetrahydrofuran@mail.ru (Y.A.P.); faiz.eline2000@yandex.ru (E.A.F.); malabuiokdiana@gmail.com (D.M.M.); aigul.r.ibragimova@gmail.com (A.R.I.); yuliana256@mail.ru (Y.A.M.); oxana.serova@gmail.com (O.V.S.); gpopova305@gmail.com (G.P.P.); droplbox38@gmail.com (M.Y.Z.); timanikitin36@gmail.com (T.D.N.); v.brylev@yandex.ru (V.A.B.); austinov@yandex.ru (A.V.U.); alferovava@gmail.com (V.A.A.); v-korshun@yandex.ru (V.A.K.); smirnov@ibch.ru (I.V.S.); sterekhoff@gmail.com (S.S.T.); 2 Higher Chemical College of the Russian Academy of Sciences, D. Mendeleev University of Chemical Technology of Russia, Miusskaya Square 9, 125047 Moscow, Russia

**Keywords:** antibody–drug conjugates, enzymatic modification, MTGase, cleavable linkers, MMAE, branched linkers, homogeneous conjugates

## Abstract

Homogeneous antibody–drug conjugates (ADCs) exhibit significantly improved pharmacological properties compared to their heterogeneous counterparts. Site-specific conjugation of the payload to the IgG required for homogeneity can be achieved using enzymes. One example is microbial transglutaminase (MTGase), which can selectively perform transamidation on the Q295 residue of human Fc when N297 glycans are removed. As a result, two modifications can be introduced per IgG molecule; however, achieving higher drug-to-antibody ratios (DARs) requires the use of branched linkers. While several such linkers have been reported, little information is available on the relationship between linker structure and ADC properties. To address this gap, we synthesized two branched amino triazide linkers, differing by a PEG_4_ fragment inserted after the branching point, which were used to prepare two homogeneous trastuzumab-based DAR 6 ADCs (a “short” and a “long” one). This was achieved by a two-step process consisting of enzymatic linker conjugation followed by bioorthogonal coupling with a cleavable linker bearing monomethyl auristatin E (MMAE). Two other trastuzumab–MMAE conjugates were used as controls: a heterogeneous DAR 6 ADC, made using conventional thiol–maleimide chemistry, and a homogeneous DAR 2 ADC. We found that, while the four conjugates had identical affinity for HER2, their cytotoxicity differed significantly: the “long” homogeneous DAR 6 ADC was just as active as its heterogeneous counterpart, but the “short” DAR 6 ADC was an order of magnitude less potent, inferior even to the DAR 2 conjugate. Our findings indicate that the length of the branched linker critically affects the cytotoxic activity of ADCs, possibly due to steric hindrance influencing the rate of linker cleavage by lysosomal enzymes.

## 1. Introduction

The concept of targeted drug delivery to tumor cells has been implemented in the form of antibody–drug conjugates (ADCs). This concept has been known since the 1950s, but only in recent years has the technology gained traction. This is due to the fact that ADCs are a complex combination of key components, such as antibodies, linkers, and drugs, each of which has a significant impact on the overall result [1]. It should be noted that the optimization of these components has received a lot of attention from both academic scientists and R&D departments of large pharmaceutical companies. Different trends in ADCs have succeeded each other as the technology has evolved, and the understanding of exactly how these drugs work in the human body has deepened [2].

**Figure 1:**
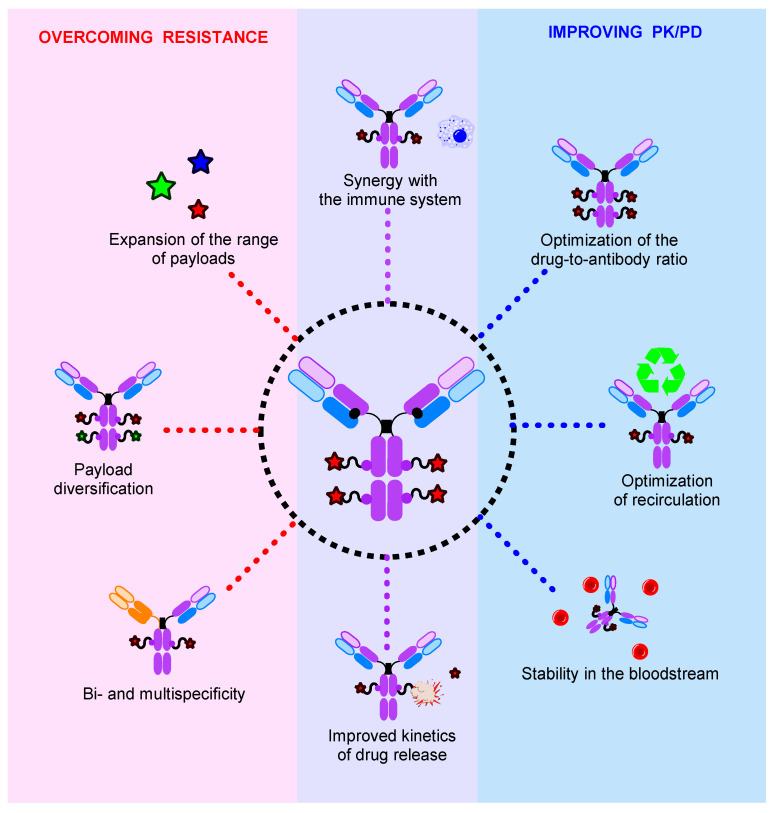
Main trends in modern ADC design.

**Figure 2:**
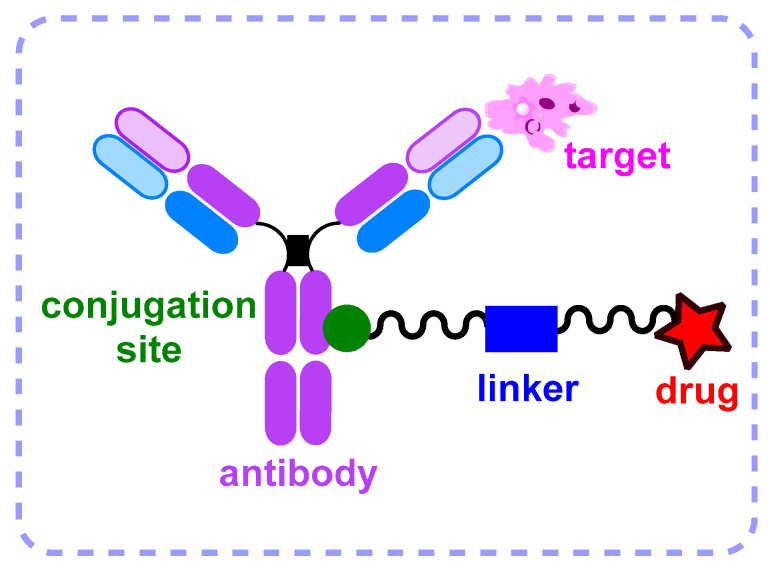
Optimization of the structural elements of an ADC can improve its therapeutic potential.

The most prominent trends in ADC development in recent years can be divided into two broad groups: overcoming resistance [3] and improving the pharmacological properties (Figure 1) [4]. The first group includes the expansion of the range of drugs used; their diversification [5,6,7,8], including the involvement of drugs with a new mechanism of action [9]; the use of bi- and multispecific antibodies [10]; and the multi-payload approach [11]. The second group may include the optimization of antibody recirculation in the body and the drug–antibody ratio (DAR), increased stability in the bloodstream, and improved drug release kinetics and synergistic interaction with the immune system [12]. All of these goals can be achieved by optimizing the key parts of the conjugate (Figure 2).

One of the most important aspects of ADC design is the conjugation of the linker to the antibody. Since the inception of the field, relatively few conjugation methods have been developed. Some of the first and most popular are conjugation to the amino groups of lysine residues within the antibody using NHS esters and conjugation to the reduced cysteines of the disulfide bonds involved in maintaining the structure of the antibody using maleimides. These approaches are well established in ADC drugs on the market; 11 of the 12 conjugates approved by the FDA for use in patients to date (not including Aidixi^TM^ from RemeGen, which is currently only approved in China) [13] have been made using these approaches. However, despite the apparent simplicity and efficiency of these approaches, they have a number of drawbacks. One of them is the high heterogeneity of the resulting conjugates, i.e., the presence of antibodies with various drug-to-antibody ratios and even unconjugated antibodies in the mixture. This leads to a deterioration of the pharmacological properties; in particular, the more loaded species are eliminated faster and can damage liver cells. On the other hand, unconjugated antibodies compete with conjugates for binding to the target and also reduce the efficacy of the conjugate.

The only homogeneous conjugates currently available for use in patients (approved by the FDA in 2019 and 2020, respectively) are Enhertu^TM^ (trastuzumab deruxtecan), a superhit drug developed by Daiichi-Sankyo for the treatment of breast and gastric cancer, and Trodelvy^TM^ (sacituzumab govitecan), developed by the Gilead Sciences company for the treatment of metastatic triple-negative breast cancer and metastatic urothelial cancer. The conjugates were prepared by exhaustive reduction of the native disulfide bonds of the antibody, followed by modification of the resulting cysteines with maleimides, to achieve the maximum drug-to-antibody ratio of eight and, thus, homogeneity. However, this approach can only be applied to conjugates with hydrophilic camptothecin derivatives, such as DXd and SN38, since for other cytotoxic payloads such a high DAR results in a significant increase in hydrophobicity, uptake by liver cells, and rapid clearance from the bloodstream [4].

**Figure 3:**
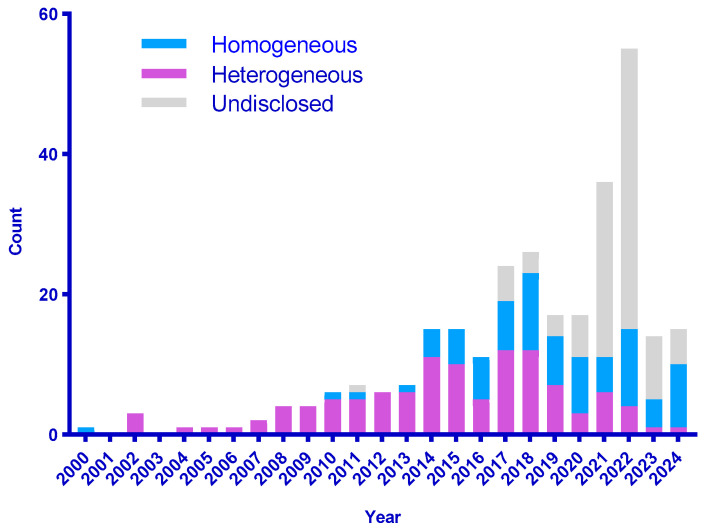
Introduction of homogeneous and heterogeneous ADCs into clinical trials by year.

The development of homogeneous conjugates with a precisely defined drug–antibody ratio is a prominent trend in ADC development [14,15]. From around the 2010s to the present day, the percentage of homogeneous conjugates among those entering clinical trials has steadily increased, reaching more than 50% in the last two years, 2023–2024 (Figure 3) [16].

Currently, there are several approaches to obtaining strictly homogeneous conjugates, with varying degrees of technology complexity [11,15]. These methods include the introduction of additional cysteines, unnatural amino acids, or peptide tags into the antibody backbone; rebridging of natural cysteines; glycan modification; and various enzymatic approaches. Some of the conjugates obtained by such methods have reached the clinical trials stage, and their number is increasing every year [16].

The Thiomab^TM^ technology from Genentech (a member of the Roche group) is one of the technologies that has entered clinical trials [17]. This technology relies on the introduction of additional cysteines by genetic engineering and their subsequent selective labeling with conventional or stabilized self-hydrolyzed maleimides [18]. Another variant of cysteine labeling is exhaustive cross-linking of antibody disulfides using bifunctional reagents, the so-called disulfide rebridging. In this case, genetically engineered cysteine residues are not required; the use of natural cysteines allows a minimum of four drug molecules per antibody [19]. Synaffix’s GlycoConnect^TM^ technology, which involves remodeling glycans with the introduction of a bioorthogonal azido group, followed by a click reaction with a proprietary hydrophilized linker, HydraSpace^TM^, has also gained popularity [20]. Approaches such as the introduction of non-natural amino acids with biorthogonal groups followed by linker modifications are less popular due to the high complexity of the technology [21].

**Figure 4:**
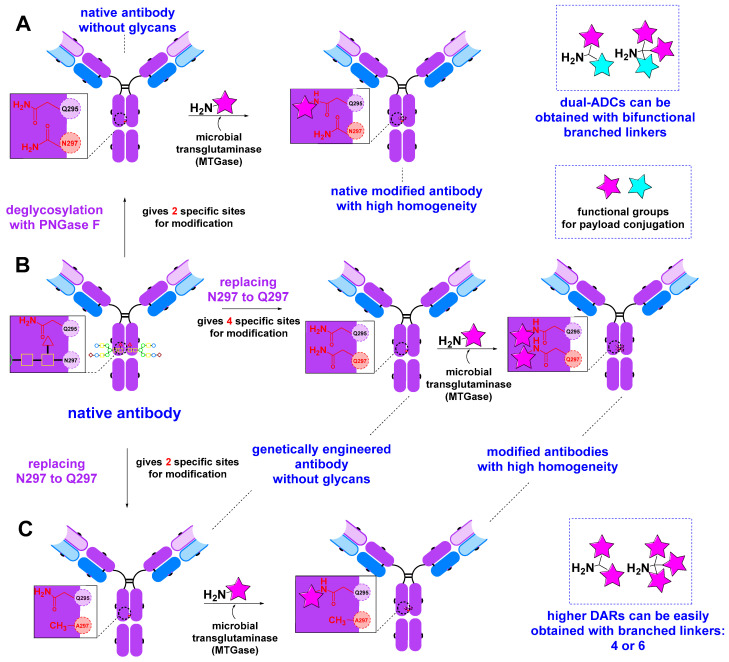
Labeling of antibody with MTGase: key strategies. (**A**) Deglycosylation of the native antibody and subsequent enzymatic labeling yields a conjugate with a DAR of 2. (**B**) Genetically engineered replacement of N297 with Q297 and subsequent enzymatic labeling yields a conjugate with a DAR of 4; deglycosylation of the antibody is not required. (**C**) Genetically engineered replacement of N297 with A297 and subsequent enzymatic labeling yields a conjugate with a DAR of 2; deglycosylation of the antibody is not required [22,23,24].

Methods using natural enzymes are also promising because they use mild conditions and do not interfere with antigen binding. Microbial transglutaminase (MTGase)-based approaches are among the most popular (Figure 4).

The advantages of this method are its low cost, versatility, high site-specificity, and the homogeneity of the products obtained, which, in turn, leads to improved pharmacological properties. The method is based on enzymatic recognition and ligation of a substrate to a specific site on the antibody. In the case of microbial transglutaminase, this is the glutamine 295 (Q295) residue on the Fc fragment of the antibody. It is normally inaccessible due to shielding by the glycans attached to the neighboring asparagine 297 residue (N297). The glycans can be removed by another enzyme, PNGase F (Figure 4A), or by sequence engineering to replace N297 with an A297 residue (Figure 4C). Without the involvement of genetic engineering, it is possible to obtain a conjugate with a maximum DAR of two, because the antibody has two Q295 sites. To increase the DAR, it is possible to use branched linkers (Figure 4A,C) or to introduce additional sites by engineering the antibody (Figure 4B). 

**Figure 5:**
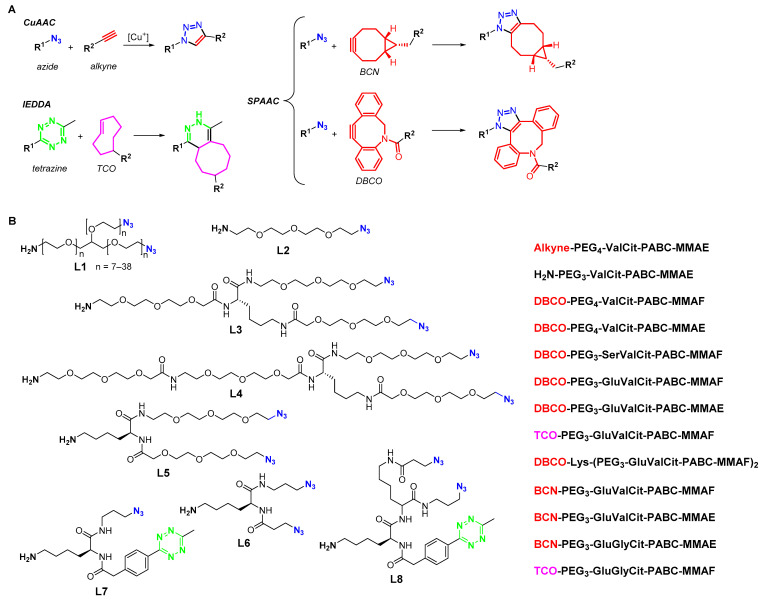
(**A**) Bioorthogonal click reactions; (**B**) functional amino-linkers used for MTGase-mediated antibody modification (**L1**–**L8**) and functional payload derivatives. For full structures, see Figure S1 in the Supplementary Materials.

Linkers with an amino group are suitable as substrates for MTGases. The linker can contain a payload in the structure and be linked to the antibody by the enzyme [25], or it can be functionalized with an appropriate functional group for further conjugation with the payload [4]. Figure 5 shows the linkers and functional payload derivatives used in different strategies of MTGase-mediated ADC assembly.

Direct MTGase-mediated modification of an antibody with an amino payload derivative, H_2_N-PEG_3_-ValCit-PABC-MMAE, has been reported [25]. However, the strategy of enzymatically assembling the conjugate in a single step has found limited use because MTGase-mediated conjugation requires a large excess of the linker (200–400 equivalents), making the use of a pre-assembled linker-payload rather expensive; in addition, payload solubility issues may arise.

Stepwise chemoenzymatic assembly of ADCs has been reported more frequently. An early patent [26] reported the conjugation of branched amino diazide linkers of different lengths (**L1**) using MTGase, followed by the copper-catalyzed click modification of the azido groups with Alkyne-PEG_4_-ValCit-PABC-MMAE. Dennler et al. [27] used a two-step procedure for a DAR 2 ADC: MTGase-mediated antibody modification with a short amino azide linker, **L2**, followed by strain-promoted azide-alkyne cycloaddition (SPAAC) reaction with DBCO-PEG_4_-ValCit-PABC-MMAE. Anami et al. [22] extended this approach to branched amino azide linkers of different lengths (**L3**–**L5**) and investigated the conditions of the MTGase-catalyzed reaction. A further SPAAC reaction with DBCO-PEG_4_-ValCit-PABC-MMAF afforded ADCs with a DAR of four. It was found that the arm length of a branched linker conjugated to a cleavable linker has a significant effect on the efficiency of subsequent cleavage by cathepsin. A linker that was too short was found to dramatically reduce the rate of hydrolysis of the Val-Cit-PAB fragment, probably due to steric hindrance; the introduction of a PEG_3_ moiety after the branching point was shown to solve this problem. Subsequent papers, also from the Tsuchikama group [23,24], reported DAR 4 ADCs that were prepared with the short branching linker **L5** using SPAAC and novel cathepsin B-cleavable tripeptide linkers, DBCO-PEG_3_-SerValCit-PABC-MMAF and DBCO-PEG_3_-GluValCit-PABC-MMAF.

In a 2021 publication [28], trifunctional amino-azido-tetrazine and amino-diazido-tetrazine linkers, **L7** and **L8**, were proposed for the chemoenzymatic assembly of homogeneous conjugates, and a series of new payload derivatives, DBCO-PEG_3_-GluValCit-PABC-MMAE and TCO-PEG_3_-GluValCit-PABC-MMAF, and even a bis-payload compound, DBCO-Lys-(PEG_3_-GluValCit-PABC-MMAF)_2_, were reported. These building blocks allow the assembly of homogeneous dual-drug ADCs, such as MMAE/F 4+2. Later, the Tsuchikama group used a less hydrophobic (as compared to DBCO) BCN function for SPAAC (the BCN-PEG_3_-GluValCit-PABC-MMAF and BCN-PEG_3_-GluValCit-PABC-MMAE reagents) [29,30] and discovered a novel cathepsin-cleavable tripeptide linker, Glu-Gly-Cit (EGCit), with improved hydrophilicity and stability in mouse and human plasma [30]. This linker was used in several payload derivatives, e.g., BCN-PEG_3_-GluGlyCit-PABC-MMAE and TCO-PEG_3_-GluGlyCit-PABC-MMAF.

Several papers have been devoted to the analysis of the length of hydrophilizing PEG [31,32] and polysarcosine moieties [33] and the way they are placed in the linker-payload molecule. It has been found that the use of a long PEG molecule, e.g., PEG_12_ or PEG_24_, as a linear linker with a payload at its end, leads to increased exposure of the hydrophobic part of the linker and the payload to the aqueous medium and to an increased overall hydrophobicity of the conjugate. This leads to uptake by liver cells and rapid clearance of the conjugate from the bloodstream, reducing the exposure and the therapeutic effect of the conjugate. At the same time, the use of masking PEG inserts of a similar length, placed as a side branch of the linker, leads to an increase in the overall hydrophilicity of the conjugate and an improvement in its pharmacological properties. However, these works do not investigate the patterns of optimal linker length for branched-core linkers for conjugates with higher DAR, only for linear ones. Such an architecture, in which a branched structure with multiple linker-payload molecules is conjugated to a single site on the antibody, requires special attention: a local increase in hydrophobicity on the surface of the antibody can lead to aggregation. Nevertheless, many approaches to the production of homogeneous ADCs, such as enzymatic labeling, glycan remodeling, and others, rely on this architecture [11].

While a considerable number of branched ADC linkers with different architectures have been reported (Figure 5), there is limited information on the relationship between the branched linker structure and ADC activity. Furthermore, among the hundreds of ADCs developed by private companies [34], there are many examples with claimed branched linkers of undisclosed structure. Thus, it remains unclear what constitutes the optimal design of a branched-linker-based ADC synthesis platform. In the present work, we sought to address this gap by synthesizing branched linkers of different lengths and directly comparing the conjugates based on them.

To this end, we chose to use site-specific MTGase-mediated antibody modification with branched amino-triazide linkers of different lengths, followed by strain-promoted azide-alkyne cycloaddition (SPAAC) conjugation with the payload. For the payload part, we used the clinically validated monomethyl auristatin E (MMAE), conjugated to the hydrophilic EGCit linker. Our results indicate that the activity of the ADC is dependent on the linker length, with a shorter branched linker performing significantly worse than a longer one with an additional PEG_4_ moiety.

## 2. Results and Discussion

### 2.1. ADC Synthesis and Characterization

**Scheme 1:**
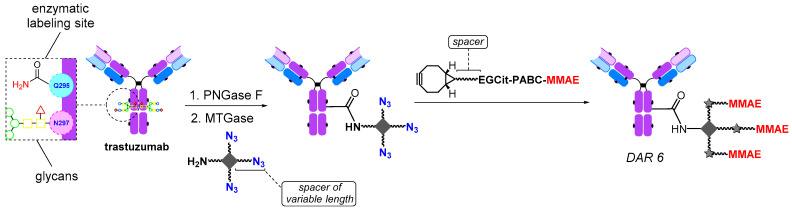
General ADC synthesis scheme.

To determine the influence of the structure of a branched linker on ADC activity, we set out to synthesize homogeneous DAR 6 conjugates with varying linker lengths. We aimed to perform site-specific MTGase-mediated modification of the deglycosylated therapeutic IgG1 antibody trastuzumab with branched amino triazide linkers, followed by SPAAC conjugation of an EGCit-based cleavable linker bearing monomethyl auristatin E (MMAE), as per Scheme 1.

**Scheme 2:**
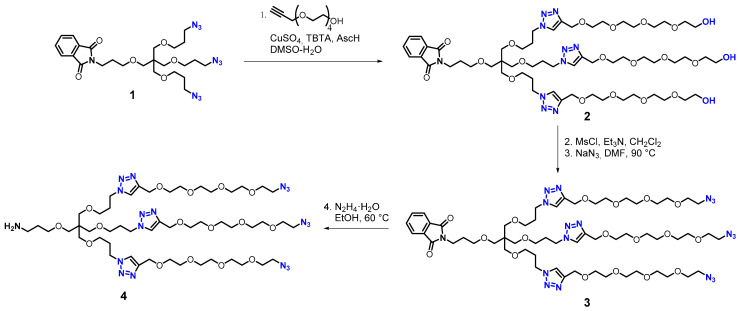
Synthesis of branched amino triazide linker **4**.

To this end, we extended the previously reported triazide **1** [35] with tetraethylene glycol monopropargyl ether using copper-catalyzed azide-alkyne cycloaddition (CuAAC) to obtain triol **2**. Notably, the use of the CuSO_4_-TBTA-ascorbic acid combination, as opposed to the more conventional CuI [36], allowed for facile separation of the copper catalyst and isolation of the pure product. Following a two-step mesylation/azidation procedure, the extended triazide **3** was isolated; subsequent phthalimide deprotection yielded amino triazide **4** (Scheme 2).

**Scheme 3:**
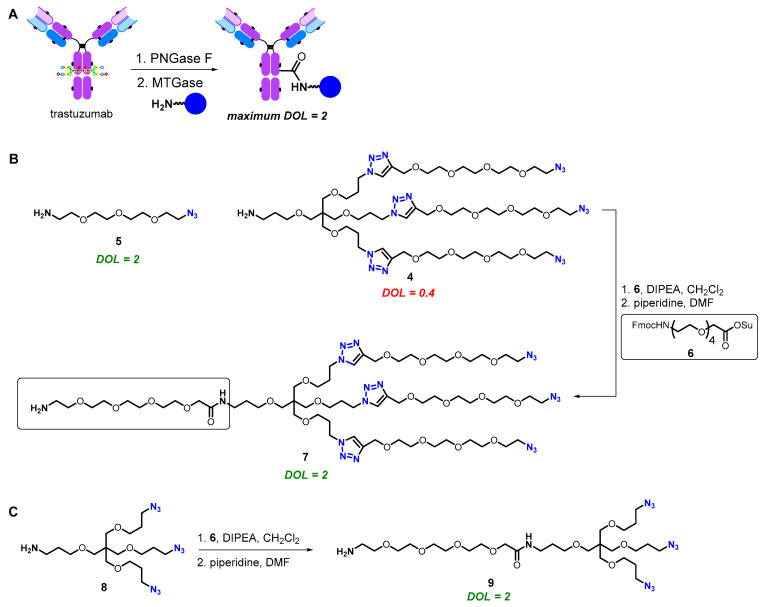
General scheme of MTGase-mediated conjugation (**A**); Synthesis of amino-PEG_4_-triazide linkers **7** (**B**) and **9** (**C**). DOL—degree of labeling.

With linker **4** in hand, we attempted its conjugation to deglycosylated trastuzumab (Scheme 3A). In our hands, MTGase-mediated conjugation initially proved challenging, failing to furnish fully conjugated products for a number of substrates. After extensive optimization, we were able to achieve nearly complete conversion with amino azide **5**. However, under optimized conditions, the conjugation with **4** proceeded with low efficiency (Scheme 3B). We attributed this to the insufficient distance between the amine moiety and the bulky branching point of the linker. Since microbial transglutaminase is known to accept sterically unhindered primary amines as substrates [22,37], we added a PEG_4_ moiety to **4** in two synthetic steps, obtaining triazide **7** (Scheme 3B), which was used to modify trastuzumab with complete conversion. It should be noted that the enzymatic reaction requires specific concentrations of the antibody (more than 1 mg/mL, preferably 5–10 mg/mL) and MTGase (0.08% *w*/*v*), and no agitation should be used.

Based on the information that an excessively long linker connecting the antibody and the therapeutic payload molecule leads to a decrease in efficacy [31,32,33], we decided to synthesize a shorter, branched linker, **9**, with an architecture similar to linker **7**, in order to compare the effect of spacer length in the branched linker core on the properties of the conjugate.

The shorter linker, **9**, had the optimal amino spacer length for the reaction with MTGase that we had previously determined (Scheme 3B) and shorter (by one PEG_4_ unit) azido spacers for subsequent click reaction with the MMAE-containing moiety **19**.

**Scheme 4:**
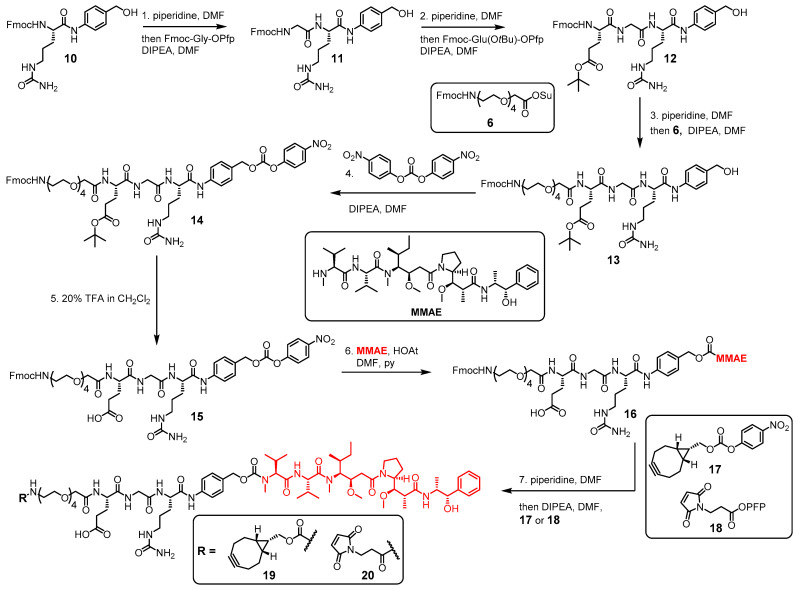
Synthesis of EGCit-PABC-MMAE-based reagents **19** and **20**.

Linker **19** is an EGCit tripeptide linker that was modified with a reactive cyclooctyne group with a PEG_4_ fragment and an MMAE therapeutic payload molecule on the other side (Scheme 4). This linker architecture was chosen due to the fact that EGCit is an improved version of the classic, well-established cathepsin B-cleavable Val-Cit linker used in most approved ADCs. This tripeptide linker is more hydrophilic and stable in plasma and is not cleaved by the neutrophil elastase [30]. The PEG_4_ fragment was introduced to further improve the hydrophilicity of the conjugate. We chose *endo*-BCN over the commonly used, more stable DBCO because of its higher reactivity and our previous observation that DBCO, when used for conjugation with antibodies modified by branched linkers containing azido groups, results in a significantly increased propensity for conjugates to aggregate [35].

The synthesis of the tripeptide by the solid-phase method has been described in the literature [30]. We developed our own approach for the synthesis of EGCit-PAB-OH in a solution by performing a stepwise assembly of the target linker (Scheme 4). We obtained the activated carbonate intermediate **15** and then the MMAE derivative **16**, which allowed us to install any functional group of interest for further antibody modification. As a result of the synthesis, we obtained the BCN derivative of the linker-MMAE **19** and a similarly structured maleimide derivative, **20**.

**Figure 6:**
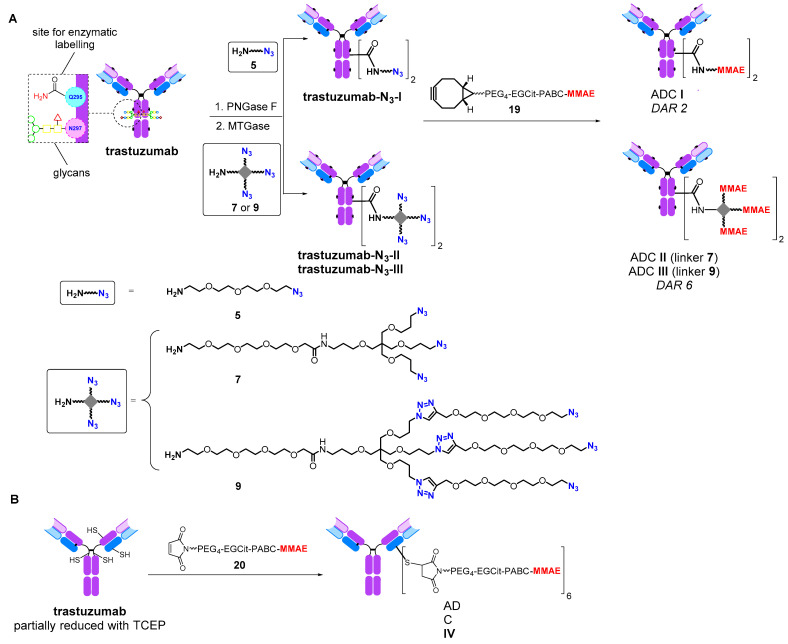
The synthesis of ADCs **I**–**III** (**A**) and ADC **IV** (**B**).

The BCN-bearing linker-payload **19** was used to synthesize ADCs **I**–**III** from azido-modified trastuzumab (Figure 6A). In turn, maleimide **20** was used to synthesize the heterogeneous ADC **IV** by labeling native cysteine residues of the interchain disulfide bonds of the antibody with a comparable DAR value (Figure 6B). Such a heterogeneous conjugate is a necessary control for evaluating the efficacy of homogeneous derivatives. Under in vitro conditions, heterogeneous conjugates obtained by maleimide labeling are usually as active as homogeneous conjugates. The drug-to-antibody ratio of the conjugates was determined spectrophotometrically (Section 3.6); ADCs **II**, **III**, and **IV** had a DAR of six, while ADC **I** had a DAR value of two.

**Figure 7:**
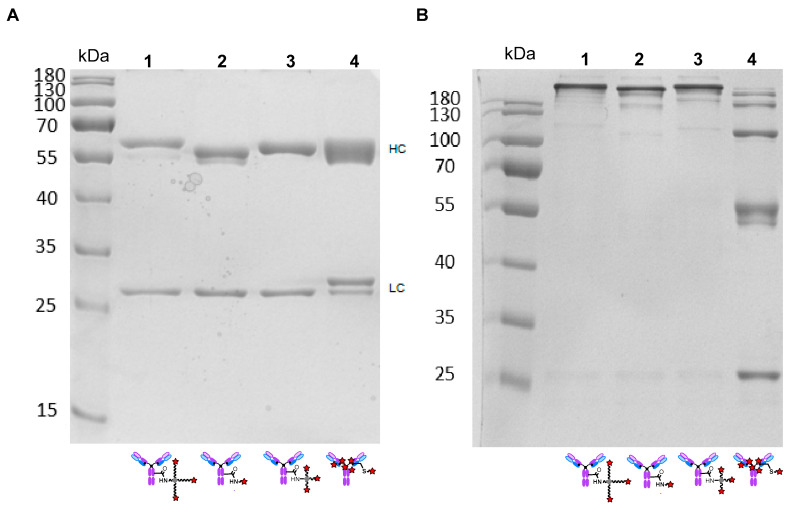
SDS-PAGE of conjugates. (**A**) Reducing conditions; (**B**) non-reducing conditions. Lane; 1—ADC **III**; 2—ADC **I**; 3—ADC **II**; 4—ADC **IV**. Conjugates in the figure caption are presented in native form for clarity.

The resulting conjugates, ADC **I**–**IV,** were then examined by SDS-PAGE under reducing (Figure 7A) and non-reducing conditions (Figure 7B). It can be seen that the enzymatic labeling proceeds with a high conversion (Figure 7A, lanes 1–3), especially for branched linkers **7** and **9**. In the case of the linear linker **5**, an unreacted deglycosylated antibody is observed (Figure 7A, lane 2). In all cases, the light chains are not affected. It can also be observed that the SPAAC conjugation of BCN-MMAE **19** proceeds quantitatively. For the heterogeneous conjugate ADC **IV** (Figure 7A, lane 4), the modification of both heavy and light chains is observed. Under non-reducing conditions, it can be observed that the integrity of the antibody is preserved in ADCs **I**–**III** (Figure 7B, lanes 1–3), whereas in the case of ADC **IV**, a number of bands are seen due to the partial reduction of interchain disulfide bonds (Figure 7B, lane 4).

**Figure 8:**
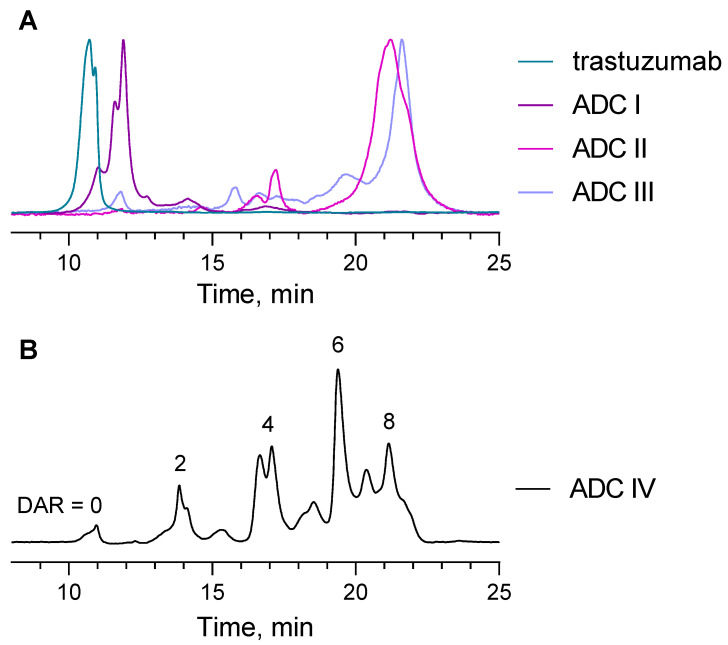
HIC traces of trastuzumab and ADCs **I**–**III** (**A**) and ADC **IV** (**B**).

The conjugates were also analyzed by hydrophobic interaction chromatography (HIC) (Figure 8). The results show that conjugates **I**–**III** have high homogeneity (Figure 8A), whereas the heterogeneous ADC **IV** conjugate consists of a mixture of species with different DAR values (Figure 8B). The average DAR value for ADC **IV,** calculated from the HIC trace, is 5.4, which is in good agreement with the UV/Vis data.

Interestingly, ADC **III** shows a slightly longer retention time compared to ADC **II**, i.e., it is slightly more hydrophobic. This is due to the fact that, in both cases, the hydrophobic payload moieties located at the ends of the long linker arms are exposed to the aqueous medium, and this exposure is the more efficient the longer the linker [31]. Thus, in our ADC architecture, the inclusion of an additional PEG_4_ into the branched linker slightly increases the hydrophobicity of the ADC, even though the linker itself becomes more hydrophilic.

### 2.2. ELISA

**Figure 9:**
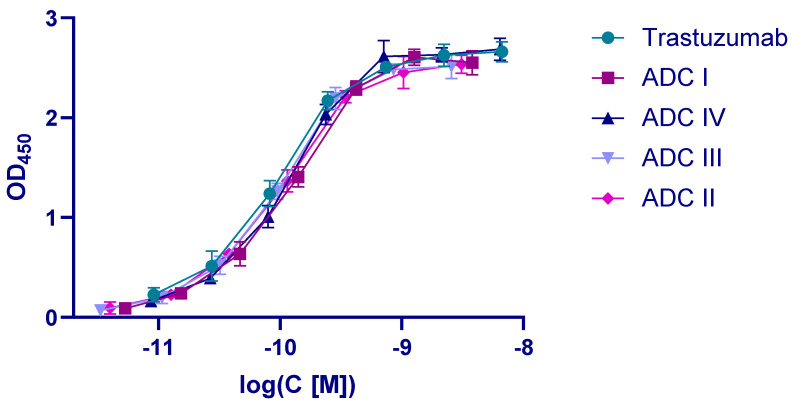
Antigen-binding properties of trastuzumab and ADCs **I**–**IV,** analyzed by ELISA.

To determine whether the enzymatic reaction and payload conjugation affected target binding, we performed an ELISA assay using immobilized recombinant extracellular domain of human HER2. We found that both the enzymatically constructed ADCs **I**–**III** and the heterogeneous ADC **IV** showed no loss of affinity compared to trastuzumab (Figure 9).

### 2.3. In Vitro Cytotoxicity Assay

**Figure 10:**
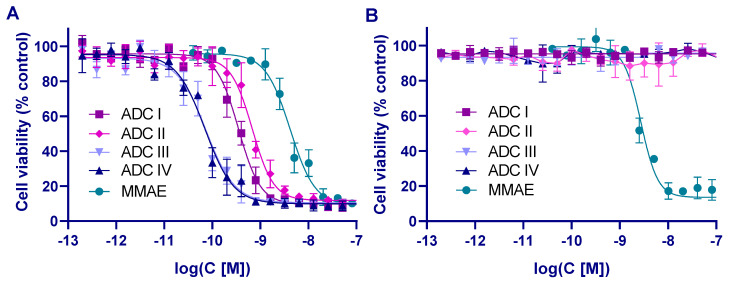
The cytotoxicity of ADCs **I**–**IV** and MMAE towards (**A**) BT-474 and (**B**) MDA-MB-231 cells. The molarity of **I**–**IV** refers to the whole ADC.

The cytotoxicity of ADCs **I**–**IV** was evaluated using HER2^+^ BT-474 and control triple negative MDA-MB-231 human breast carcinoma cell lines; free MMAE was used as a control. Figure 10A shows the toxicity of the conjugates against BT-474 cells, expressed as the percentage of surviving cells. Figure 10B shows a similar graph for MDA-MB-231 cells.

By approximating the obtained dose–effect relationships (Figure 10), the following IC_50_ values were obtained for the ADCs in BT-474 cells (Table 1).

Both the BT-474 and MDA-MB-231 cell lines were sensitive to free MMAE (IC_50_ values of 4.2 and 2.7 nM, respectively). The ADCs, however, were found to be highly selective for HER2-expressing cells, with no effect on the HER2-negative MDA-MB-231 cell line at concentrations up to 100 nM. ADC **I** was less potent (IC_50_ = 0.35 nM) than both ADC **III** (0.074 nM) and ADC **IV** (0.071 nM). Here, a threefold increase in the DAR resulted in a comparable increase in activity, which was consistent with the literature. More interestingly, ADC **II**, the DAR 6 conjugate with a short, branched linker, proved to be less active (IC_50_ = 0.68 nM) than the other two enzymatically constructed ADCs **I** and **III**. In fact, the latter had the same activity as the heterogeneous DAR 6 ADC **IV**. 

The low activity of ADC **II** compared to ADC **III** was a rather unexpected phenomenon, which can be explained by the fact that the branched core of the linker sterically interferes with the action of cathepsin B and leads to a slower release of the drug in the lysosome. We draw this conclusion based on the fact that all the conjugates were tested on the same cell line with the same expression of cathepsin B, but both the short linear PEG_4_ spacer in ADC **I** and the extended branched linker in ADC **III** showed no abnormal activity compared to the heterogeneous control, while architecturally similar ADC **II** showed a decrease in activity by almost an order of magnitude compared to ADC **IV**. It should be noted that the branched core of the linker also interfered with the activity of MTGase in the case of linker **4**, albeit to a lesser extent and in a more sterically demanding context.

**Figure 11:**
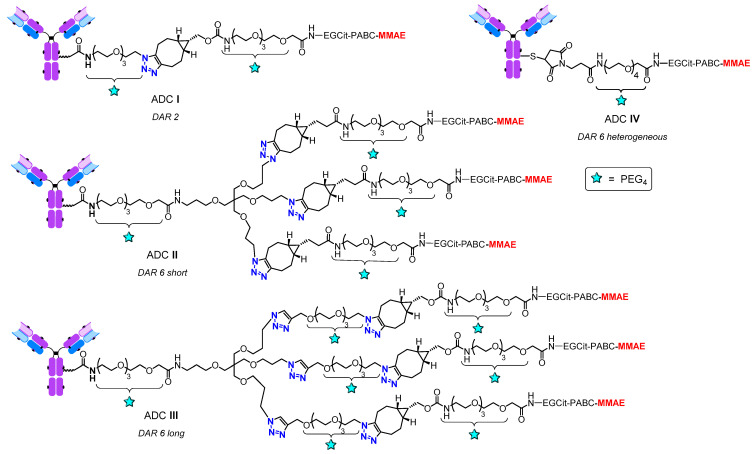
ADCs **I**–**IV**. Full structures of the branched linker parts are shown.

We can conclude that, when using branched linkers for conjugation, the distance from the branched core to the enzymatically cleavable part of the linker should be considered. In our linker design, an equivalent of a PEG_8_ fragment (in ADC **III**, there is a PEG_4_ fragment in the branched core and a PEG_4_ spacer in the cleavable linker) is sufficient, but PEG_4_ is not (Figure 11). Previously published works using enzymatic conjugation and branched linkers have not investigated the dependence of ADC activity on the structure of the linker and have not compared branched ADCs with heterogeneous DAR-matched cleavable linker–based controls [22,23,28,29,30]. To the best of our knowledge, the only available data on the efficiency of cathepsin B-mediated cleavage of branched linkers were obtained using low-molecular-weight fluorescent probes [22], and it was shown that a PEG_3_ spacer was sufficient for cathepsin B to manifest full catalytic activity towards the Val-Cit-PAB fragment, although the branched core was less sterically congested than in our case, and a low-molecular-weight compound can be expected to be cleaved more easily than an ADC. In an example from the literature, a DAR 4 trastuzumab-EGCit-PABC-MMAE conjugate assembled using a branched linker with PEG_3_ spacers was tested in the HER2^+^ BT-474 cell line used in our study, allowing comparison with our results [30]. This PEG_3_-based DAR 4 conjugate had an IC_50_ of 0.48 nM, showing an activity more similar to that of the PEG_4_-based ADC **II** (0.68 nM) than the PEG_8_-based ADC **III** (0.074 nM).

Thus, we have shown that, for branched enzymatically cleavable linkers, there is a dependence of ADC cytotoxicity on the length of the spacer between the branched core and the cleavable fragment. In contrast to conventional linear linkers, a short, branched linker may not be advantageous. The potential increase in ADC hydrophobicity because of payload exposure due to the use of long branched linkers seems problematic, but it could be overcome with additional hydrophilization. Undoubtedly, a more in-depth study of this phenomenon is needed, including more variation in spacer length and different linker architectures.

## 3. Materials and Methods

### 3.1. General Methods

All the reactions were carried out under an argon atmosphere with the dry solvents under anhydrous conditions, unless otherwise noted. Dimethylformamide (DMF) was purified by distillation with benzene/water, followed by vacuum distillation over CaH_2_, and stored over 4 Å molecular sieves. Dichloromethane (DCM) and methanol (MeOH) were purified by distillation, and dimethyl sulfoxide (DMSO) was purified by vacuum distillation and stored over 4 Å molecular sieves. The reagents were purchased at the highest commercial quality and used without further purification, unless otherwise stated. Trastuzumab was obtained from Biocad (Saint Petersburg, Russia). Microbial transglutaminase was obtained from Biopreparat (Moscow, Russia) as a 1% (*w*/*w*) formulation with dextran. Compounds **1** [35], **5** [38], **8** [35], **17** [39], **18** [40], **22** [41], Fmoc-Gly-OPfp [42], and Fmoc-Glu(t-Bu)-OPfp [42] were prepared as described previously. The reactions were monitored by thin-layer chromatography (TLC), carried out on 0.20 mm 60 F_254_ silica gel plates (Merck, Darmstadt, Germany) and using UV light as a visualizing agent and an alkaline aqueous solution of KMnO_4_, an ethanolic solution of ninhydrin (used alone for amines or in combination with a solution of triphenylphosphine in DCM for azides), and heat as developing agents; preparative TLC was carried out on the same silica gel plates using UV light as a visualizing agent. Silica gel 60 (0.040–0.063 mm) (Merck) was used for flash column chromatography. In some instances, column chromatography was performed on silica gel 60 (0.015–0.040 mm) (Merck) using a Biotage SP4 Flash Purification System (Biotage, Uppsala, Sweden). Size exclusion chromatography was performed using Sephadex G-50 (Pharmacia Fine Chemicals, Uppsala, Sweden) and Bio-Gel P-100 (Bio-Rad Laboratories, Hercules, CA, USA). Proteins were concentrated using Spin-X^®^ UF 500 µL 50,000 MWCO Centrifugal Concentrators (Corning Inc., Corning, NY, USA). UV-Vis absorbance spectra were recorded on a Cary 100 UV-Visible spectrophotometer (Agilent Technologies, Santa Clara, CA, USA). NMR spectra were recorded on 700 and 800 MHz Bruker Avance III instruments (Bruker Corporation, Billerica, MA, USA) and calibrated using a residual solvent as the internal reference (for CDCl_3_: ^1^H, δ 7.26 ppm and ^13^C, δ 77.16 ppm; for DMSO-*d*_6_: δ 2.50 ppm and ^13^C, δ 39.52 ppm), unless otherwise noted. The following abbreviations were used to represent the multiplicities: s = singlet, d = doublet, t = triplet, q = quartet, ABq = AB quartet, p = pentet, m = multiplet, app = apparent, br = broad. High-resolution mass spectra (HRMS) were recorded on a Thermo Scientific Orbitrap Exactive mass spectrometer (Thermo Fisher Scientific, Waltham, MA, USA) using ESI (Electrospray ionization).

### 3.2. Synthetic Procedures

#### 3.2.1. Triol **2**

In a 50 mL round-bottom flask, triazide **1** [35] (1.70 g, 2.97 mmol, 1 equiv.) was dissolved in DMSO (15 mL) under argon, and tetraethylene glycol monopropargyl ether (2.27 mg, 9.8 mmol, 3.3 equiv.) was added. In a separate vessel were mixed a solution of CuSO_4_⋅5H_2_O (93 mg, 0.37 mmol, 0.125 equiv.) in 1 mL H_2_O and a solution of TBTA (197 mg, 0.37 mmol, 0.125 equiv.) in 1 mL DMSO; the resultant blue-green mixture was allowed to cool to 23 °C and added to the reaction mixture, which turned pale blue. Ascorbic acid (650 mg, 3.7 mmol, 1.25 equiv.) was then added to the mixture, upon which the reaction mixture turned colorless. The reaction mixture was stirred at 23 °C for 2 h, after which, TLC analysis (silica gel, hexane:EtOAc 2:1) indicated a complete conversion of **1**. The reaction mixture was then transferred to a separatory funnel, diluted with DCM (300 mL), and washed with water (6 × 50 mL) to remove the DMSO. The organic layer was dried over anhydrous Na_2_SO_4_ and concentrated under reduced pressure. The resultant residue was purified by flash column chromatography (silica gel, DCM–MeOH, 10:1→7:1) to afford triol **2** (2.55 g, 68%) as a colorless viscous oil. *R*_f_ 0.34 (silica gel, DCM–MeOH, 7:1). ^1^H NMR (800 MHz, DMSO-*d*_6_) δ 8.04 (s, 3H), 7.85–7.82 (m, 2H), 7.82–7.79 (m, 2H), 4.54 (t, *J* = 5.5 Hz, 3H), 4.50 (s, 6H), 4.37 (t, *J* = 7.1 Hz, 6H), 3.64 (t, *J* = 6.9 Hz, 2H), 3.56–3.44 (m, 42H), 3.42–3.37 (m, 8H), 3.30 (t, *J* = 6.0 Hz, 6H), 3.28 (s, 2H), 3.23 (s, 6H), 2.02 (p, *J* = 6.4 Hz, 6H), 1.83 (p, *J* = 6.4 Hz, 2H). ^13^C NMR (201 MHz, DMSO-*d*_6_) δ 167.9, 143.9, 134.3, 131.7, 123.7, 122.9, 72.3, 69.78, 69.75 (two overlapping signals), 69.73, 69.65, 69.2, 69.1, 68.9, 68.7, 67.3, 63.5, 60.2, 46.6, 44.9, 35.2, 29.8, 28.0. HRMS (ESI) calcd for C_58_H_98_N_10_O_21_^2+^ [M+2H]^2+^ 635.3449, found 635.3475.

#### 3.2.2. Trimesylate **21**

**Scheme 5:**
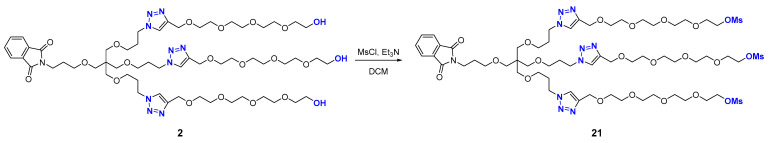
Synthesis of trimesylate **21**.

In a 100 mL round-bottom flask, triol **2** (2.19 g, 1.72 mmol, 1 equiv.) was dissolved in DCM (30 mL) and cooled to 0 °C (ice bath), after which triethylamine (3.35 mL, 2.43 g, 24.1 mmol, 14 equiv.) was added. The reaction mixture was stirred at 0 °C for 15 min, which was followed by dropwise addition of a solution of MsCl (1.33 mL, 1.97 g, 17.2 mmol, 10 equiv.) in DCM (20 mL) from a dropping funnel. The reaction mixture was stirred at 0 °C for 15 min, after which the ice bath was removed and the reaction mixture was stirred at 23 °C for 3 h. As the TLC analysis (silica gel, DCM–MeOH, 7:1) indicated a complete conversion of the triol, the reaction mixture was transferred to a separatory funnel, diluted with DCM (150 mL), and washed with water (4 × 30 mL). The organic layer was dried over anhydrous Na_2_SO_4_ and concentrated under reduced pressure. The resultant crude trimesylate **21** (Scheme 5) was obtained as a dark yellow viscous oil (2.67 g), which was then coevaporated with EtOAc (3 × 50 mL) to remove the residual DCM and used in the next step without further purification. *R*_f_ 0.28 (silica gel, EtOAc–MeOH, 2:1). ^1^H NMR (700 MHz, DMSO-*d*_6_) δ 8.04 (s, 3H), 7.85–7.82 (m, 2H), 7.82–7.79 (m, 2H), 4.50 (s, 6H), 4.37 (t, *J* = 7.1 Hz, 6H), 4.31–4.28 (m, 6H), 3.68–3.65 (m, 6H), 3.64 (t, *J* = 6.9 Hz, 2H), 3.57–3.47 (m, 36H), 3.40 (t, *J* = 5.8 Hz, 2H), 3.30 (t, *J* = 6.9 Hz, 6H), 3.28 (s, 2H), 3.23 (s, 6H), 3.16 (s, 9H), 2.02 (p, *J* = 6.5 Hz, 6H), 1.83 (p, *J* = 6.4 Hz, 2H).

#### 3.2.3. Triazide **3**

**Scheme 6:**
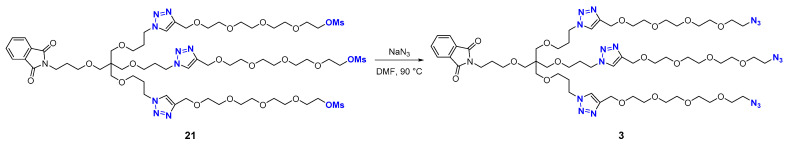
Synthesis of triazide **3**.

In a 100 mL round-bottom flask, crude trimesylate **21** (2.67 g) obtained in the previous step was dissolved in DMF (35 mL), and NaN_3_ (0.84 g, 12.9 mmol, 7.5 equiv.) was added. The reaction mixture was heated to 90 °C and stirred for 18 h. As TLC analysis (silica gel, EtOAc–MeOH, 2:1) indicated full conversion, the reaction mixture was cooled to 23 °C, and the DMF was removed on a rotary evaporator (bath temperature: 50 °C). The resultant light-brown oil was cooled to −18 °C (freezer) and triturated with THF (40 mL) at 23 °C. The resultant precipitate (sodium mesylate) was filtered out on Celite and washed with THF (60 mL). The THF solution was concentrated under reduced pressure to yield a light-brown oil, which was purified by flash column chromatography (silica gel, EtOAc–MeOH, 10:1→8:1→6:1→4:1→3:1) to afford triazide **3** (Scheme 6) as a colorless viscous oil (1.93 g, 83% over two steps). *R*_f_ 0.50 (silica gel, EtOAc–MeOH, 2:1). ^1^H NMR (700 MHz, DMSO-*d*_6_) δ 8.04 (s, 3H), 7.85–7.82 (m, 2H), 7.82–7.79 (m, 2H), 4.50 (s, 6H), 4.37 (t, *J* = 7.0 Hz, 6H), 3.64 (t, *J* = 6.9 Hz, 2H), 3.59 (app t, *J* = 5.0 Hz, 6H), 3.56–3.47 (m, 36H), 3.40 (t, *J* = 5.9 Hz, 2H), 3.37 (app t, *J* = 5.0 Hz, 6H), 3.30 (t, *J* = 6.0 Hz, 6H), 3.28 (s, 2H), 3.23 (s, 6H), 2.02 (p, *J* = 6.4 Hz, 6H), 1.83 (p, *J* = 6.4 Hz, 2H). ^13^C NMR (176 MHz, DMSO-*d*_6_) δ 167.8, 143.9, 134.2, 131.7, 123.7, 122.9, 69.76, 69.74, 69.72, 69.6 (two overlapping signals), 69.21, 69.17, 69.05, 68.9, 68.7, 67.3, 63.5, 49.9, 46.5, 44.8, 35.2, 29.7, 28.0. HRMS (ESI) calcd for C_58_H_95_N_19_O_18_^2+^ [M+2H]^2+^ 672.8546, found 635.8575.

#### 3.2.4. Amino Triazide **4**

In a 50 mL round-bottom flask, triazide **3** (1.93 g, 1.44 mmol, 1 equiv.) was dissolved in 96% EtOH (15 mL). N_2_H_4_∙H_2_O (1.40 mL, 1.44 g, 28.8 mmol, 20 equiv.) was added, and the reaction mixture was stirred at 60 °C for 1 h, after which more N_2_H_4_∙H_2_O (0.200 mL, 0.205 g, 4.1 mmol, 2.8 equiv.) was added, and the reaction mixture was stirred at 60 °C for 30 more min. In the course of the reaction, white flaky precipitate formed. As TLC analysis (EtOAc–MeOH, 3:1) indicated full conversion, the mixture was cooled to 23 °C and carefully concentrated under reduced pressure, avoiding bumping. The resultant residue was triturated with THF (30 mL) and filtered through a pad of Celite (washing with THF). The filtrate was concentrated under reduced pressure, the resultant residue was purified by flash column chromatography (silica gel, DCM–MeOH–Et_3_N, 3:1:0→2:1:0→1:1:0→1:1:0.05) to yield amino triazide **4** as a viscous yellowish oil (1.34 g, 77%). *R*_f_ 0.31 (silica gel, EtOAc–MeOH–Et_3_N, 1:1:0.1). ^1^H NMR (800 MHz, DMSO-*d*_6_) δ 8.05 (s, 3H), 4.51 (s, 6H), 4.39 (t, *J* = 7.0 Hz, 6H), 3.59 (app t, *J* = 5.0 Hz, 6H), 3.56–3.49 (m, 36H), 3.39 (t, *J* = 6.3 Hz, 2H), 3.38 (app t, *J* = 5.0 Hz, 6H), 3.34 (t, *J* = 6.0 Hz, 7H), 3.29 (s, 8H), 2.57 (t, *J* = 6.8 Hz, 2H), 2.04 (p, *J* = 6.5 Hz, 6H), 1.55 (p, *J* = 6.5 Hz, 2H). ^13^C NMR (201 MHz, DMSO-*d*_6_) δ 143.9, 123.7, 69.79, 69.77, 69.75, 69.66 (two overlapping signals), 69.2, 69.1, 68.95, 68.93, 68.89, 67.3, 63.5, 50.0, 46.6, 44.9, 38.8, 32.9, 29.8. HRMS (ESI) calcd for C_50_H_93_N_19_O_16_^2+^ [M+2H]^2+^ 607.8518, found 607.8561.

#### 3.2.5. FmocNH-PEG_4_-CH_2_CO_2_NSu **6**

**Scheme 7:**
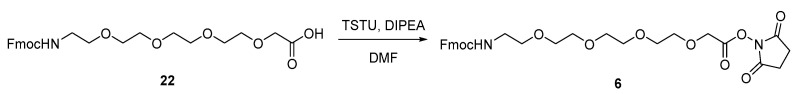
Synthesis of NHS ester **6**.

In a 50 mL round-bottom flask, FmocNH-PEG_4_-CH_2_CO_2_H **22** [41] (3.52 g, 7.43 mmol, 1 equiv.) was dissolved in dry DMF (15 mL). DIPEA (1.68 mL, 1.25 g, 9.66 mmol, 1.3 equiv.) was added, followed by TSTU (2.91 g, 9.66 nmol, 1.3 equiv.) The reaction mixture was stirred at 23 °C for 10 min, at which point TLC analysis (silica gel, DCM–MeOH–AcOH, 5:1:0.06) indicated a complete conversion of **22**. The mixture was diluted with EtOAc (50 mL) and washed with 1% *w*/*w* aqueous HCl (4 × 50 mL). The organic layer was dried over anhydrous Na_2_SO_4_ and concentrated under reduced pressure to yield FmocNH-PEG_4_-CH_2_CO_2_NSu **6** (Scheme 7) as a viscous yellowish oil (3.15 g, 74%), which was used in the subsequent steps without further purification. *R*_f_ 0.30 (silica gel, hexane–acetone, 1:1). ^1^H NMR (800 MHz, CDCl_3_) δ 7.75 (d, *J* = 7.5 Hz, 2H), 7.60 (d, *J* = 7.5 Hz, 2H), 7.39 (t, *J* = 7.4 Hz, 2H), 7.31 (t, *J* = 7.4 Hz, 2H), 5.40 (br t, *J* = 5.3 Hz, 1H), 4.52 (s, 0.3H, minor rotamer), 4.48 (s, 1.7H, major rotamer), 4.40 (d, *J* = 7.0 Hz, 2H), 4.22 (t, *J* = 7.0 Hz, 1H), 3.82–3.51 (m, 14H), 3.38 (q, *J* = 5.3 Hz, 2H), 2.81 (s, 4H). ^13^C NMR (201 MHz, CDCl_3_) δ 168.8, 166.1, 156.6, 144.1, 141.4, 127.7, 127.1, 125.1, 120.0, 71.4, 70.7, 70.61, 70.59 (two overlapping signals), 70.4, 70.1, 66.6 (two overlapping signals by HSQC), 47.4, 41.0, 25.6. HRMS (ESI) calcd for C_29_H_38_N_3_O_10_^+^ [M+NH_4_]^+^ 588.2552, found 588.2575.

#### 3.2.6. Triazide **23**

**Scheme 8:**
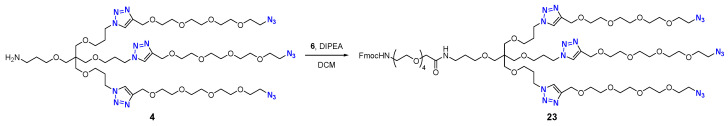
Synthesis of triazide **23**.

In a 2 mL polypropylene microcentrifuge tube, amino triazide **4** (100 mg, 0.082 mmol, 1 equiv.) was dissolved in DCM (1 mL). DIPEA (21.5 µL, 16.0 mg, 0.123 mmol, 1.5 equiv.) was added, followed by a solution of FmocNH-PEG_4_-CH_2_CO_2_NSu **6** (70.5 mg, 0.123 mmol, 1.5 equiv) in DCM (0.2 mL). The reaction mixture was agitated on an orbital shaker at 23 °C for 1 h, after which TLC (silica gel, EtOAc–MeOH–Et_3_N, 1:1:0.1) showed complete consumption of **4**. The reaction mixture was transferred to a round-bottom flask and concentrated under reduced pressure. The resultant residue was purified by flash column chromatography (silica gel, DCM–MeOH, 30:1→25:1) to afford triazide **23** (Scheme 8) as a colorless viscous oil (95 mg, 69%). *R*_f_ 0.48 (silica gel, DCM–MeOH, 9:1). ^1^H NMR (800 MHz, DMSO-*d*_6_) δ 8.04 (s, 3H), 7.88 (d, *J* = 7.5 Hz, 2H), 7.68 (d, *J* = 7.5 Hz, 2H), 7.58 (t, *J* = 5.9 Hz, 1H), 7.41 (t, *J* = 7.4 Hz, 2H), 7.32 (t, *J* = 7.4 Hz, 2H), 7.28 (t, *J* = 5.9 Hz, 1H), 4.51 (s, 6H), 4.38 (t, *J* = 7.0 Hz, 6H), 4.29 (d, *J* = 7.0 Hz, 2H), 4.20 (t, *J* = 7.0 Hz, 1H), 3.84 (s, 2H), 3.58 (app t, *J* = 7.0 Hz, 6H), 3.57–3.47 (m, 48H), 3.40 (t, *J* = 6.0 Hz, 2H), 3.37 (t, *J* = 4.9 Hz, 6H), 3.36 (m, 1H), 3.33 (t, *J* = 6.1 Hz, 6H), 3.29 (s, 8H), 3.15 (q, *J* = 6.6 Hz, 2H), 3.13 (q, *J* = 5.9 Hz, 1H), 2.04 (p, *J* = 6.5 Hz, 6H), 1.65 (p, *J* = 6.6 Hz, 2H). ^13^C NMR (201 MHz, DMSO-*d*_6_) δ 169.0, 156.1, 143.92, 143.87, 140.7, 127.5, 127.0, 125.1, 123.7, 120.0, 70.2, 69.9, 69.79, 69.77, 69.75, 69.71, 69.66, 69.54, 69.50, 69.2, 69.07, 69.03, 68.9, 68.7, 67.3, 65.3, 63.5, 50.0, 46.7, 46.6, 44.9, 40.1, 35.6, 29.8, 29.3. HRMS (ESI) calcd for C_75_H_122_N_20_O_23_^2+^ [M+2H]^2+^ 835.4490, found 835.4506.

#### 3.2.7. Amino Triazide **7**

**Scheme 9:**
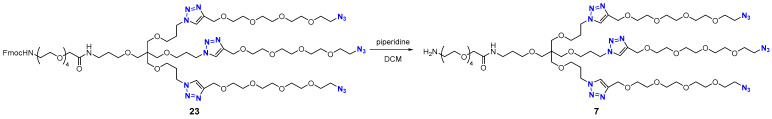
Synthesis of amino triazide **7**.

In a 10 mL round-bottom flask, triazide **23** (59.6 mg, 0.036 mmol, 1 equiv.) was dissolved in DCM (2 mL), and piperidine (0.4 mL) was added. The reaction mixture was stirred at 23 °C for 2 h and concentrated under reduced pressure. The resultant white solid residue was coevaporated with DCM (3 × 5 mL) and purified by flash column chromatography (silica gel, DCM–MeOH, 1:0→10:1) to afford amino triazide **7** (Scheme 9) as a colorless viscous oil (21 mg, 50%). *R*_f_ 0.43 (silica gel, EtOAc–MeOH–Et_3_N, 1:1:0.1). ^1^H NMR (800 MHz, DMSO-*d*_6_) δ 8.23 (br s, 2H), 8.06 (s, 3H), 7.67 (t, *J* = 5.8 Hz, 1H), 4.51 (s, 6H), 4.39 (t, *J* = 7.1 Hz, 6H), 3.86 (s, 2H), 3.60 (m, 2H), 3.59 (app t, *J* = 5.0 Hz, 6H), 3.57–3.48 (m, 48H), 3.374 (t, *J* = 5.0 Hz, 6H), 3.367 (t, *J* = 6.6 Hz, 2H), 3.34 (t, *J* = 6.0 Hz, 6H), 3.30 (s, 8H), 3.15 (q, *J* = 6.6 Hz, 2H), 2.95 (t, *J* = 5.3 Hz, 2H), 2.04 (p, *J* = 6.4 Hz, 6H), 1.65 (p, *J* = 6.6 Hz, 2H). ^13^C NMR (201 MHz, DMSO-*d*_6_) δ 169.0, 143.9, 123.7, 70.1, 69.9, 69.78, 69.76, 69.74, 69.68, 69.65, 69.58, 69.55, 69.52, 69.20, 69.08, 69.05, 68.93, 68.7, 67.4, 66.8, 63.5, 50.0, 46.6, 44.9, 38.6, 35.7, 29.8, 29.2. HRMS (ESI) calcd for C_60_H_112_N_20_O_21_^2+^ [M+2H]^2+^ 724.4150, found 724.4182.

#### 3.2.8. Triazide **24**

**Scheme 10:**
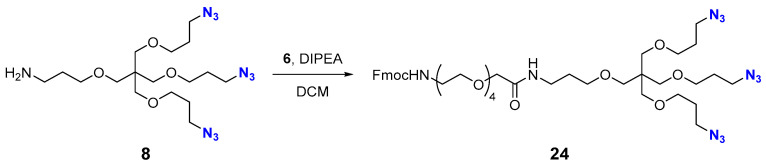
Synthesis of triazide **24**.

In a 10 mL round-bottom flask, amino triazide **8** [41] (326 mg, 0.737 mmol, 1.4 equiv.) was dissolved in DCM (2 mL). DIPEA (0.095 mL, 70 mg, 0.542 mmol, 1 equiv.) was added, followed by a solution of FmocNH-PEG_4_-CH_2_CO_2_NSu **6** (309 mg, 0.542 mmol, 1 equiv.) in DCM (1 mL). The reaction mixture was stirred at 23 °C for 2.5 h (the conversion of **6** could not be easily tracked by TLC since **6** has the same *R*_f_ as **24** in a range of systems), then transferred to a separatory funnel, diluted with DCM (10 mL), and washed with saturated aqueous NH_4_Cl (10 mL). The organic layer was dried over anhydrous Na_2_SO_4_ and concentrated under reduced pressure. The resultant residue was purified by flash column chromatography (silica gel, DCM–EtOAc, 1:1→1:3→0:1) to afford triazide **24** (Scheme 10) as a yellow viscous oil (248 mg, 51%). *R*_f_ 0.26 (silica gel, EtOAc). ^1^H NMR (800 MHz, DMSO-*d*_6,_ major rotamer) δ 7.88 (d, *J* = 7.5 Hz, 2H), 7.69 (d, *J* = 7.5 Hz, 2H), 7.57 (t, *J* = 5.9 Hz, 1H), 7.41 (t, *J* = 7.4 Hz, 2H), 7.32 (t, *J* = 7.3 Hz, 2H), 7.28 (t, *J* = 5.8 Hz, 1H), 4.29 (d, *J* = 7.1 Hz, 2H), 4.21 (t, *J* = 7.0 Hz, 1H), 3.85 (s, 2H), 3.60–3.47 (m, 12H), 3.41 (t, *J* = 5.9 Hz, 2H), 3.40 (t, *J* = 6.0 Hz, 6H), 3.36 (t, *J* = 6.8 Hz, 6H), 3.34 (t, *J* = 6.2 Hz, 2H), 3.29 (s, 6H), 3.28 (s, 2H), 3.16 (q, *J* = 6.6 Hz, 2H), 3.14 (q, *J* = 5.8 Hz, 2H), 1.73 (p, *J* = 6.4 Hz, 6H), 1.65 (p, *J* = 6.6 Hz, 2H). ^13^C NMR (201 MHz, DMSO-*d*_6,_ major rotamer) δ 168.9, 156.1, 143.9, 140.7, 127.5, 127.0, 125.1, 120.0, 70.2, 70.0, 69.76, 69.72, 69.55, 69.51, 69.1, 69.0, 68.8, 67.5, 65.3, 47.8, 46.7, 44.9, 40.1, 35.7, 29.3, 28.4. HRMS (ESI) calcd for C_42_H_64_N_11_O_11_^+^ [M+H]^+^ 898.4781, found 898.4819.

#### 3.2.9. Amino Triazide **9**

**Scheme 11:**
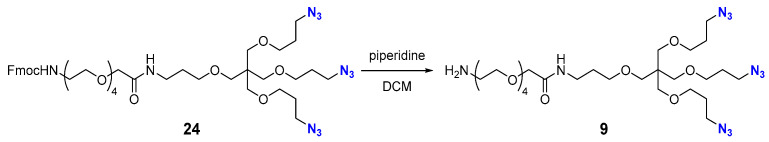
Synthesis of amino triazide **9**.

In a 2 mL polypropylene microcentrifuge tube, triazide **24** (40 mg, 0.044 mmol) was dissolved in DCM (0.5 mL), and piperidine (0.1 mL) was added. The reaction mixture was agitated on an orbital shaker at 23 °C for 1.5 h and concentrated under reduced pressure. The resultant white solid residue was purified by flash column chromatography (DCM–MeOH–Et_3_N, 1:0:0→2:1:0→1:1:0→1:1:0.02) to afford amino triazide **9** (Scheme 11) as a yellowish oil (10.5 mg, 35%). *R*_f_ 0.43 (silica gel, EtOAc–MeOH–Et_3_N, 1:1:0.1). ^1^H NMR (800 MHz, DMSO-*d*_6_) δ 7.94 (br s, 2H), 7.67 (t, *J* = 5.9 Hz, 1H), 3.87 (s, 2H), 3.61 (t, *J* = 5.3 Hz, 2H), 3.59–3.52 (m, 12H), 3.41 (t, *J* = 6.0 Hz, 6H), 3.37 (t, *J* = 6.7 Hz, 6H), 3.35 (t, *J* = 6.5 Hz, 2H), 3.30 (s, 6H), 3.29 (s, 2H), 3.16 (q, *J* = 6.7 Hz, 2H), 2.96 (t, *J* = 5.3 Hz, 2H), 1.74 (p, *J* = 6.4 Hz, 6H), 1.65 (p, *J* = 6.5 Hz, 2H). ^13^C NMR (201 MHz, DMSO-*d*_6_) δ 169.0, 70.1, 69.9, 69.7, 69.58, 69.55, 69.53, 69.10, 69.05, 68.7, 67.5, 66.6, 47.8, 44.9, 38.5, 35.7, 29.2, 28.4. HRMS (ESI) calcd for C_42_H_64_N_11_O_11_^+^ [M+H]^+^ 676.4101, found 676.4138.

#### 3.2.10. Fmoc-Gly-Cit-PAB-OH **11**

In a 2 mL polypropylene microcentrifuge tube, Fmoc-Cit-PAB-OH **10** [43] (200 mg, 0.398 mmol, 1 equiv.) was dissolved in dry DMF (0.6 mL), and piperidine (40 μL, 34 mg, 0.405 mmol, 1.02 equiv.) was added. The reaction mixture was agitated on an orbital shaker at 23 °C for 15 min, at which point a considerable amount of white precipitate formed, and TLC (silica gel, DCM–MeOH, 5:1) indicated a full conversion of **10**. The reaction mixture was slightly diluted with DMF (100 μL), and the product was precipitated portionwise in a 2 mL polypropylene microcentrifuge tube by the addition of Et_2_O (1.8 mL) to 100 μL aliquots of the reaction mixture, followed by centrifugation and supernatant removal. The combined precipitate was washed with Et_2_O (3 × 1.8 mL) with sonication and dried under vacuum to afford crude H-Cit-PAB-OH as a tan solid; *R*_f_ 0.23 (silica gel, *n*-BuOH–H_2_O–AcOH, 5:1:1).

The crude H-Cit-PAB-OH was dissolved in dry DMF (0.4 mL), and DIPEA (83 μL, 62 mg, 0.478 mmol, 1.2 equiv.) was added, followed by solid Fmoc-Gly-OPfp [42] (221 mg, 0.478 mmol, 1.2 equiv.) The reaction mixture was agitated on an orbital shaker at 23 °C for 30 min, at which point, TLC (silica gel, *n*-BuOH–H_2_O–AcOH, 5:1:1) indicated a full conversion of H-Cit-PAB-OH. The product was precipitated portionwise in a 2 mL polypropylene microcentrifuge tube by the addition of Et_2_O (1.8 mL) to 100 μL aliquots of the reaction mixture, followed by centrifugation and supernatant removal. The combined precipitate was washed with Et_2_O (3 × 1.8 mL) with sonication and dried under a vacuum to afford crude Fmoc-Gly-Cit-PAB-OH **11** (246 mg) as a tan solid, which was used in the subsequent step without further purification. For characterization, a 50 mg portion of the crude product was purified by flash column chromatography (silica gel, DCM–MeOH, 0–10% linear gradient, Biotage SP4 Flash Purification System) to afford pure **11** (17 mg) as a yellow solid. *R*_f_ 0.33 (silica gel, DCM–MeOH, 5:1). ^1^H NMR (800 MHz, DMSO-*d*_6_) δ 9.96 (s, 1H), 8.13 (d, *J* = 8.0 Hz, 1H), 7.89 (d, *J* = 7.5 Hz, 2H), 7.71 (d, *J* = 7.5 Hz, 2H), 7.56 (d, *J* = 8.2 Hz, 2H), 7.54 (t, *J* = 6.2 Hz, 1H), 7.41 (app td, *J* = 7.4, 3.1 Hz, 2H), 7.33 (t, *J* = 7.4 Hz, 2H), 7.24 (d, *J* = 8.2 Hz, 2H), 5.98 (br t, *J* = 5.9 Hz, 1H), 5.40 (br s, 2H), 5.09 (t, *J* = 5.7 Hz, 1H), 4.45 (m, 1H), 4.43 (d, *J* = 5.7 Hz, 2H), 4.31–4.25 (m, 2H), 4.23 (t, *J* = 7.1 Hz, 1H), 3.70 (d, *J* = 6.2 Hz, 2H), 3.01 (dq, *J* = 13.4, 6.8 Hz, 1H), 2.95 (dq, *J* = 13.1, 6.4 Hz, 1H), 1.76–1.66 (m, 1H), 1.63–1.53 (m, 1H), 1.49–1.40 (m, 1H), 1.40–1.32 (m, 1H). ^13^C NMR (201 MHz, DMSO-*d*_6_) δ 170.4, 169.0, 158.8, 156.5, 143.8, 140.7, 137.5, 137.4, 127.6, 127.1, 126.9, 125.2, 120.1, 119.0, 65.7, 62.6, 52.9, 46.6, 43.3, 29.7, 26.7. HRMS (ESI) calcd for C_30_H_33_N_5_O_6_Na^+^ [M+Na]^+^ 582.2323, found 582.2324.

#### 3.2.11. Fmoc-Glu(*t*-Bu)-Gly-Cit-PAB-OH **12**

In a 2 mL polypropylene microcentrifuge tube, crude Fmoc-Gly-Cit-PAB-OH **11** (192 mg, assumed 0.343 mmol, 1 equiv.) was dissolved in dry DMF (0.4 mL), and piperidine (0.169 mL, 146 mg, 1.72 mmol, 5 equiv.) was added. The reaction mixture was agitated on an orbital shaker at 23 °C for 5 min. A considerable amount of white precipitate formed, so more DMF (200 μL) was added, and the reaction mixture was agitated at 23 °C for 15 more min, at which point, TLC (silica gel, DCM–MeOH, 5:1) indicated a full conversion of **11**. The reaction mixture was further diluted with DMF (100 μL), and the product was precipitated portionwise in a 2 mL polypropylene microcentrifuge tube by the addition of Et_2_O (1.8 mL) to 100 μL aliquots of the reaction mixture, followed by centrifugation and supernatant removal. The combined precipitate was washed with Et_2_O (3 × 1.8 mL) with sonication and dried under vacuum to afford crude H-Gly-Cit-PAB-OH as a white solid; *R*_f_ 0.18 (silica gel, *n*-BuOH–H_2_O–AcOH, 5:1:1).

The crude H-Gly-Cit-PAB-OH was dissolved in dry DMF (0.5 mL), and DIPEA (72 μL, 53 mg, 0.412 mmol, 1.2 equiv.) was added, followed by solid Fmoc-Glu(t-Bu)-OPfp [42] (244 mg, 0.412 mmol, 1.2 equiv.) The reaction mixture was agitated on an orbital shaker at 23 °C for 30 min, at which point TLC (silica gel, *n*-BuOH–H_2_O–AcOH, 5:1:1) indicated a full conversion of H-Gly-Cit-PAB-OH. The product was precipitated portionwise in a 2 mL polypropylene microcentrifuge tube by the addition of Et_2_O (1.8 mL) to 100 μL aliquots of the reaction mixture, followed by centrifugation and supernatant removal. The combined precipitate was washed with Et_2_O (3 × 1.8 mL) with sonication and dried under a vacuum to afford crude Fmoc-Glu(*t*-Bu)-Gly-Cit-PAB-OH **12** (197 mg) as a tan solid, which was used in the next step without further purification. For characterization, the crude product was purified by flash column chromatography (silica gel, DCM–MeOH, 0–10% linear gradient, Biotage SP4 Flash Purification System) to afford pure **12** as an off-white solid. *R*_f_ 0.40 (silica gel, DCM–MeOH, 5:1). ^1^H NMR (800 MHz, DMSO-*d*_6_) δ 9.92 (s, 1H), 8.15 (t, *J* = 5.8 Hz, 1H), 8.05 (d, *J* = 7.9 Hz, 1H), 7.88 (d, *J* = 7.6 Hz, 2H), 7.72 (app dd, *J* = 16.5, 7.5 Hz, 2H), 7.58 (d, *J* = 8.0 Hz, 1H), 7.57 (d, *J* = 8.4 Hz, 2H), 7.41 (t, *J* = 7.4 Hz, 2H), 7.32 (app t, *J* = 7.4 Hz, 2H), 7.23 (d, *J* = 8.3 Hz, 2H), 5.96 (br t, *J* = 5.9 Hz, 1H), 5.40 (br s, 2H), 5.07 (t, *J* = 5.7 Hz, 1H), 4.46–4.42 (m, 1H), 4.43 (d, *J* = 5.8 Hz, 2H), 4.33 (dd, *J* = 10.4, 7.2 Hz, 1H), 4.25 (dd, *J* = 10.4, 7.1 Hz, 1H), 4.22 (t, *J* = 6.9 Hz, 1H), 4.03 (td, *J* = 8.4, 5.3 Hz, 1H), 3.77 (ABqd, Δδ_AB_ = 0.04, *J*_AB_ = 16.6 Hz, *J*_d_ = 5.7 Hz, 2H), 3.02 (dq, *J* = 13.3, 6.8 Hz, 1H), 2.94 (dq, *J* = 13.1, 6.4 Hz, 1H), 2.26 (t, *J* = 7.9 Hz, 2H), 1.97–1.89 (m, 1H), 1.81–1.74 (m, 1H), 1.74–1.68 (m, 1H), 1.58 (dtd, *J* = 13.8, 9.2, 4.8 Hz, 1H), 1.47–1.41 (m, 1H), 1.41–1.33 (m, 1H), 1.39 (s, 9H). ^13^C NMR (201 MHz, DMSO-*d*_6_) δ 171.73, 171.66, 170.3, 168.6, 158.8, 156.0, 143.9, 143.7, 140.7, 137.49, 137.40, 127.6, 127.0, 126.8, 125.3, 120.07, 120.05, 119.0, 79.6, 65.7, 62.6, 53.9, 52.9, 46.7, 42.0, 38.6, 31.3, 29.6, 27.7, 27.1, 26.7. HRMS (ESI) calcd for C_39_H_49_N_6_O_9_^+^ [M+H]^+^ 745.3556, found 745.3596.

#### 3.2.12. FmocNH-PEG_4_-Glu(*t*-Bu)-Gly-Cit-PAB-OH **13**

In a 10 mL round-bottom flask, crude Fmoc-Glu(*t*-Bu)-Gly-Cit-PAB-OH **12** (990 mg, assumed 1.33 mmol, 1 equiv.) was dissolved in dry DMF (4 mL), and piperidine (0.4 mL, 345 mg, 4.05 mmol, 3 equiv.) was added. The reaction mixture was stirred at 23 °C for 30 min, at which point TLC (silica gel, DCM–MeOH, 5:1) indicated a full conversion of **12**. The product was precipitated portionwise in a 50 mL polypropylene centrifuge tube by the addition of Et_2_O (48 mL) to 2 mL aliquots of the reaction mixture, followed by centrifugation and supernatant removal. The combined precipitate was washed with Et_2_O (2 × 30 mL) with sonication and dried under vacuum to afford crude H-Glu(*t*-Bu)-Gly-Cit-PAB-OH as a white solid; *R*_f_ 0.22 (silica gel, *n*-BuOH–H_2_O–AcOH, 5:1:1).

The crude H-Glu(*t*-Bu)-Gly-Cit-PAB-OH was dissolved in dry DMF (3 mL) in a 10 mL round-bottom flask, and DIPEA (0.232 mL,172 mg, 1.33 mmol, 1 equiv.) was added, followed by a solution of FmocNH-PEG_4_-CH_2_CO_2_NSu **6** (1.14 g, 1.99 mmol, 1.5 equiv.) in dry DMF (2 mL). The reaction mixture was stirred at 23 °C for 30 min, at which point TLC (silica gel, *n*-BuOH–H_2_O–AcOH, 5:1:1) indicated a full conversion of H-Glu(*t*-Bu)-Gly-Cit-PAB-OH. The crude product was precipitated portionwise in a 50 mL polypropylene centrifuge tube by the addition of Et_2_O (48 mL) to 2 mL aliquots of the reaction mixture, followed by centrifugation and supernatant removal. The combined precipitate was washed with Et_2_O (2 × 30 mL) with sonication and dried under a vacuum. The resultant yellow oil was purified by flash column chromatography (silica gel, DCM–MeOH, 0–10% linear gradient, Biotage SP4 Flash Purification System) to afford pure FmocNH-PEG_4_-Glu(*t*-Bu)-Gly-Cit-PAB-OH **13** (730 mg, 48% over three steps) as an off-white solid. *R*_f_ 0.40 (silica gel, DCM–MeOH, 5:1). ^1^H NMR (800 MHz, DMSO-*d*_6_, 3.6 mg/mL) δ 9.91 (s, 1H), 8.27 (t, *J* = 5.8 Hz, 1H), 8.07 (d, *J* = 7.9 Hz, 1H), 7.88 (d, *J* = 7.5 Hz, 2H), 7.75 (d, *J* = 7.9 Hz, 1H), 7.69 (d, *J* = 7.5 Hz, 2H), 7.56 (d, *J* = 8.4 Hz, 2H), 7.41 (t, *J* = 7.4 Hz, 2H), 7.32 (t, *J* = 7.4 Hz, 2H), 7.29 (t, *J* = 5.8 Hz, 1H), 7.23 (d, *J* = 8.2 Hz, 2H), 5.96 (br t, *J* = 6.0 Hz, 1H), 5.39 (br s, 2H), 5.07 (t, *J* = 5.7 Hz, 1H), 4.43 (t, *J* = 5.6 Hz, 2H), 4.44–4.40 (m, 1H), 4.33 (td, *J* = 8.1, 5.5 Hz, 1H), 4.29 (d, *J* = 7.0 Hz, 2H), 4.20 (t, *J* = 7.0 Hz, 1H), 3.92 (s, 2H), 3.77 (ABqd, Δδ_AB_ = 0.03, *J*_AB_ = 16.6 Hz, *J*_d_ = 5.8 Hz, 2H), 3.65–3.45 (m, 12H), 3.40 (t, *J* = 6.0 Hz, 2H), 3.12 (t, *J* = 6.0 Hz, 2H), 3.01 (dq, *J* = 13.2, 6.7 Hz, 1H), 2.94 (dq, *J* = 13.0, 6.4 Hz, 1H), 2.28–2.20 (m, 2H), 1.97–1.91 (m, 1H), 1.81–1.75 (m, 1H), 1.73–1.67 (m, 1H), 1.61–1.54 (m, 1H), 1.46–1.39 (m, 1H), 1.39–1.32 (m, 1H), 1.37 (s, 9H). [Note: At a higher concentration of 36 mg/mL, ^1^H and ^13^C NMR spectra are complicated by presumed intermolecular interactions (see the Supplementary Materials File S1 for details)]. ^13^C NMR (201 MHz, DMSO-*d*_6_, 36 mg/mL, multiple forms) δ 171.7, 171.2, 170.42, 170.39, 169.42, 169.40, 168.61, 168.58, 158.90, 158.89, 156.1, 143.9, 142.6, 140.7, 139.4, 137.48, 137.45, 137.41, 128.9, 127.6, 127.3, 127.0, 126.8, 125.1, 121.4, 120.07, 120.00, 119.0, 109.7, 79.7, 70.2, 69.74, 69.73, 69.70, 69.54, 69.51, 69.44, 69.1, 65.3, 62.6, 53.0, 51.4, 46.7, 41.9, 40.1, 38.5, 31.2, 29.5, 27.7, 27.4, 26.7. HRMS (ESI) calcd for C_49_H_68_N_7_O_14_^+^ [M+H]^+^ 978.4819, found 978.4821.

#### 3.2.13. FmocNH-PEG_4_-Glu(*t*-Bu)-Gly-Cit-PABC-pNP **14**

In a 2 mL polypropylene microcentrifuge tube, FmocNH-PEG_4_-Glu(*t*-Bu)-Gly-Cit-PAB-OH **13** (150 mg, 0.153 mmol, 1 equiv.) was dissolved in dry DMF (0.35 mL), and DIPEA (43 μL, 31 mg, 0.307 mmol, 2 equiv.) was added, followed by a solution of bis (4-nitrophenyl) carbonate (93 mg, 0.307 mmol, 2 equiv.) in dry DMF (0.25 mL). The reaction mixture was agitated on an orbital shaker at 23 °C for 1 h, at which point TLC (silica gel, DCM–MeOH, 7:1) indicated a full conversion of **13**. The crude product was precipitated portionwise in a 2 mL polypropylene microcentrifuge tube by the addition of Et_2_O (1.8 mL) to 100 μL aliquots of the reaction mixture, followed by centrifugation and supernatant removal. The combined precipitate was washed with Et_2_O (1.8 mL) and dried under vacuum. The resultant yellow oil was purified by flash column chromatography (silica gel, DCM–MeOH, 0–10%, linear gradient, Biotage SP4 Flash Purification System) to afford pure FmocNH-PEG_4_-Glu(*t*-Bu)-Gly-Cit-PABC-pNP **14** (125 mg, 71%) as a yellowish solid. *R*_f_ 0.62 (silica gel, DCM–MeOH, 5:1). ^1^H NMR (800 MHz, DMSO-*d*_6_) δ 10.08 (s, 1H), 8.31 (app d, *J* = 9.0 Hz, 2H), 8.28 (t, *J* = 5.8 Hz, 1H), 8.11 (d, *J* = 7.8 Hz, 1H), 7.88 (d, *J* = 7.5 Hz, 2H), 7.75 (d, *J* = 7.9 Hz, 1H), 7.68 (d, *J* = 7.8 Hz, 2H), 7.67 (d, *J* = 8.3 Hz, 2H), 7.56 (app d, *J* = 8.9 Hz, 2H), 7.43–7.39 (m, 4H), 7.32 (t, *J* = 7.4 Hz, 2H), 7.29 (t, *J* = 5.7 Hz, 1H), 5.97 (br t, *J* = 5.9 Hz, 1H), 5.40 (br s, 2H), 5.24 (s, 2H), 4.43 (td, *J* = 8.4, 5.2 Hz, 1H), 4.33 (td, *J* = 8.1, 5.5 Hz, 1H), 4.29 (d, *J* = 7.1 Hz, 2H), 4.20 (t, *J* = 7.0 Hz, 1H), 3.92 (s, 2H), 3.77 (ABqd, Δδ_AB_ = 0.03, *J*_AB_ = 16.7 Hz, *J*_d_ = 5.8 Hz, 2H), 3.64–3.46 (m, 12H), 3.40 (t, *J* = 6.0 Hz, 2H), 3.13 (q, *J* = 5.9 Hz, 1H), 3.03 (dq, *J* = 13.4, 6.8 Hz, 1H), 2.94 (dq, *J* = 12.9, 6.4 Hz, 1H), 2.29–2.18 (m, 2H), 2.00–1.90 (m, 1H), 1.83–1.75 (m, 1H), 1.75–1.68 (m, 1H), 1.59 (dtd, *J* = 13.9, 9.3, 4.8 Hz, 1H), 1.48–1.40 (m, 1H), 1.40–1.33 (m, 1H), 1.37 (s, 9H). ^13^C NMR (201 MHz, DMSO-*d*_6_) δ 171.6, 171.2, 170.7, 169.4, 168.6, 158.9, 156.1, 155.3, 151.9, 145.2, 143.9, 140.7, 139.3, 129.40, 129.36, 127.6, 127.0, 125.4, 125.1, 122.6, 120.1, 119.2, 79.7, 70.2, 69.76, 69.73, 69.72, 69.69, 69.5, 69.2, 69.1, 65.3, 53.0, 51.4, 46.7, 41.9, 40.1, 38.5, 31.2, 29.4, 27.7, 27.4, 26.7. HRMS (ESI) calcd for C_56_H_71_N_8_O_18_^+^ [M+H]^+^ 1143.4881, found 1143.4868.

#### 3.2.14. FmocNH-PEG_4_-Glu-Gly-Cit-PABC-pNP **15**

In a 10 mL round-bottom flask, to FmocNH-PEG_4_-Glu(*t*-Bu)-Gly-Cit-PABC-pNP, **14** (100 mg, 0.088 mmol) was added a 20% *v*/*v* solution of TFA in DCM (4.2 mL, prepared under argon). The reaction mixture was stirred at 23 °C for 1.5 h, at which point TLC (silica gel, DCM–MeOH, 5:1) indicated a full conversion of **14**. The mixture was concentrated under reduced pressure, and the resultant yellow residue was coevaporated with DCM (5 mL) and purified by flash column chromatography (silica gel, DCM–MeOH, 0–20%, linear gradient, Biotage SP4 Flash Purification System) to afford pure FmocNH-PEG_4_-Glu-Gly-Cit-PABC-pNP **15** (48 mg, 50%) as a yellowish solid. *R*_f_ 0.51 (silica gel, DCM–MeOH–AcOH, 3:1:0.04). ^1^H NMR (800 MHz, DMSO-*d*_6_) δ 12.08 (br s, 1H), 10.09 (s, 1H), 8.31 (app d, *J* = 9.1 Hz, 2H), 8.29 (t, *J* = 5.8 Hz, 1H), 8.13 (d, *J* = 7.8 Hz, 1H), 7.88 (d, *J* = 7.5 Hz, 2H), 7.77 (d, *J* = 7.8 Hz, 1H), 7.68 (d, *J* = 7.9 Hz, 2H), 7.67 (d, *J* = 8.5 Hz, 2H), 7.56 (app d, *J* = 9.2 Hz, 2H), 7.41 (d, *J* = 6.7 Hz, 2H), 7.40 (m, 1H), 7.32 (t, *J* = 7.4 Hz, 2H), 7.29 (t, *J* = 5.7 Hz, 1H), 6.00 (br s, 1H), 5.41 (br s, 2H), 5.24 (s, 2H), 4.43 (td, *J* = 8.1, 5.4 Hz, 1H), 4.34 (td, *J* = 8.0, 5.6 Hz, 1H), 4.29 (d, *J* = 7.0 Hz, 2H), 4.20 (t, *J* = 7.1 Hz, 1H), 3.92 (s, 2H), 3.77 (ABqd, Δδ_AB_ = 0.05, *J*_AB_ = 16.6 Hz, *J*_d_ = 5.8 Hz, 2H), 3.62–3.43 (m, 12H), 3.40 (t, *J* = 6.0 Hz, 2H), 3.13 (q, *J* = 5.9 Hz, 2H), 3.02 (dq, *J* = 13.4, 6.7 Hz, 1H), 2.94 (dq, *J* = 12.9, 6.4 Hz, 1H), 2.25 (t, *J* = 8.0 Hz, 2H), 1.96 (dq, *J* = 13.4, 7.2 Hz, 1H), 1.82 (dq, *J* = 13.4, 8.0 Hz, 1H), 1.74–1.67 (m, 1H), 1.64–1.56 (m, 1H), 1.47–1.40 (m, 1H), 1.40–1.32 (m, 1H). [Note: The compound is prone to decomposition in DMSO-*d*_6_]. HRMS (ESI) calcd for C_52_H_64_N_8_O_18_^2+^ [M+2H]^2+^ 544.2164, found 544.2166.

#### 3.2.15. FmocNH-PEG_4_-Glu-Gly-Cit-PABC-MMAE **16**

In a 2 mL polypropylene microcentrifuge tube with a screw cap, to monomethyl auristatin E (38.5 mg, 0.054 mmol, 1.1 equiv.) was added a solution of FmocNH-PEG_4_-Glu-Gly-Cit-PABC-pNP **15** (51.6 mg, 0.047 mmol, 1 equiv.) in dry DMF (0.4 mL), followed by dry pyridine (56 μL, 55 mg, 0.70 mmol, 15 equiv.) and a solution of HOAt (10.9 mg, 0.080 mmol, 1.7 equiv.) in dry DMF (50 μL). The microcentrifuge tube was purged with argon and agitated on an orbital shaker at 23 °C for 16 h, at which point TLC (silica gel, DCM–MeOH–AcOH, 5:1:0.06, run twice) indicated a full conversion of **15** (note: due to very similar *R*_f_ values, the starting material and the product could only be differentiated by heating the TLC plate: **15** turned yellow, **16** did not). The crude product was precipitated portionwise in a 2 mL polypropylene microcentrifuge tube by the addition of Et_2_O (1.8 mL) to 80 μL aliquots of the reaction mixture, followed by centrifugation and supernatant removal. The combined precipitate was washed with Et_2_O (1.8 mL) and dried under a vacuum. The resultant white solid was purified by flash column chromatography (silica gel, DCM–MeOH, 9:1→7:1→5:1→4:1→3:1→2:1) to afford pure FmocNH-PEG_4_-Glu-Gly-Cit-PABC-MMAE **16** (43.2 mg, 55%) as a yellowish glassy solid, which turned into white powder upon treatment with Et_2_O and drying under a vacuum. *R*_f_ 0.57 (silica gel, DCM–MeOH–AcOH, 3:1:0.04). ^1^H NMR (800 MHz, DMSO-*d*_6_, two rotamers) 10.26 (s, 0.5H), 10.22 (s, 0.5H), 8.60 (br s, 1H), 8.46 (br s, 1H), 8.30– 8.24 (m, 1H), 8.01 (br s, 1H), 7.89 (d, *J* = 7.5 Hz, 2H), 7.69 (d, *J* = 7.5 Hz, 2H), 7.66–7.60 (m, 2H), 7.41 (t, *J* = 7.4 Hz, 2H), 7.36–7.24 (m, 9H), 7.21–7.14 (m, 1H), 6.61 (br s, 1H), 5.60 (br s, 2H), 5.43 (br s, 0.5H), 5.35 (br s, 0.5H), 5.15–4.91 (m, 2H), 4.74 (br s, 1H), 4.63 (br s, 1H), 4.50 (d, *J* = 6.0 Hz, 1H), 4.47–4.39 (m, 1H), 4.39–4.31 (m, 2H), 4.31–4.23 (m, 3H), 4.21 (t, *J* = 7.1 Hz, 1H), 4.07–3.88 (m, 4H), 3.88–3.81 (m, 1H), 3.79 (dd, *J* = 9.4, 2.4 Hz, 0.5H), 3.67–3.44 (m, 15H), 3.42–3.39 (m, 2H), 3.25 (s, 1.5H), 3.24 (s, 1.5H), 3.21 (s, 1.5H), 3.19 (s, 1.5H), 3.14 (q, *J* = 5.9 Hz, 2H), 3.14–3.09 (m, 1H), 3.06 (dt, *J* = 11.7, 7.1 Hz, 0.5H), 3.00–2.93 (m, 3H), 2.88 (br s, 1.5H), 2.86–2.83 (m, 1H), 2.43–2.38 (m, 1H), 2.32–2.25 (m, 1H), 2.18–2.03 (m, 4H), 2.03–1.93 (m, 1H), 1.93–1.64 (m, 7H), 1.60–1.43 (m, 3H), 1.43–1.35 (m, 1H), 1.35–1.29 (m, 1H), 1.29–1.21 (m, 1H), 1.05 (d, *J* = 6.6 Hz, 1.5H), 1.016 (d, *J* = 6.9 Hz, 1.5H), 1.014 (d, *J* = 6.8 Hz, 1.5H), 0.99 (d, *J* = 6.7 Hz, 1.5H), 0.90–0.72 (m, 18H). HRMS (ESI) calcd for C_85_H_126_N_12_O_22_^2+^ [M+2H]^2+^ 833.4549, found 833.4521.

#### 3.2.16. BCN-PEG_4_-Glu-Gly-Cit-PABC-MMAE **19**

In a 2 mL polypropylene microcentrifuge tube, FmocNH-PEG_4_-Glu-Gly-Cit-PABC-MMAE **16** (20 mg, 0.012 mmol, 1 equiv.) was dissolved in dry DMF (0.2 mL), and piperidine (22 μL, 19 mg, 0.22 mmol, 18 equiv.) was added. The reaction mixture was stirred at 23 °C for 30 min, at which point TLC (silica gel, DCM–MeOH–AcOH, 3:1:0.04) indicated a full conversion of **16**. The product was precipitated portionwise in a 2 mL polypropylene microcentrifuge tube by the addition of Et_2_O (1.8 mL) to 80 μL aliquots of the reaction mixture, followed by centrifugation and supernatant removal. The combined precipitate was washed with Et_2_O (2 × 1.8 mL) with sonication and dried under vacuum to afford crude NH_2_-PEG_4_-Glu-Gly-Cit-PABC-MMAE as a white solid; *R*_f_ 0.37 (silica gel, *n*-BuOH–H_2_O–AcOH, 3:1:1).

The crude NH_2_-PEG_4_-Glu-Gly-Cit-PABC-MMAE was dissolved in dry DMF (0.15 mL), and DIPEA (3.1 μL, 2.3 mg, 0.018 mmol, 1.5 equiv.) was added, followed by a solution of *endo*-BCN pNP carbonate **17** [39] (5.4 mg, 0.018 mmol, 1.5 equiv.) in dry DMF (50 μL). The reaction mixture was agitated on an orbital shaker at 23 °C for 2 h, at which point, TLC (silica gel, *n*-BuOH–H_2_O–AcOH, 3:1:1) indicated the full conversion of NH_2_-PEG_4_-Glu-Gly-Cit-PABC-MMAE. The crude product was precipitated portionwise in a 2 mL polypropylene microcentrifuge tube by the addition of Et_2_O (1.8 mL) to 80 μL aliquots of the reaction mixture, followed by centrifugation and supernatant removal. The combined precipitate was washed with Et_2_O (1.8 mL) and dried under a vacuum. The resultant residue was purified by flash column chromatography (silica gel, DCM–MeOH, 9:1→7:1→5:1→4:1→3:1→2:1) to afford pure BCN-PEG_4_-Glu-Gly-Cit-PABC-MMAE **19** (15.6 mg, 80%) as a yellowish glassy solid, which turned into off-white powder upon treatment with Et_2_O and drying under a vacuum. *R*_f_ 0.52 (silica gel, DCM–MeOH–AcOH, 3:1:0.04). ^1^H NMR (800 MHz, DMSO-*d*_6_, two rotamers) δ 10.13 (br s, 1H), 8.37 (br s, 1H), 8.32–8.22 (m, 1H), 8.04–7.94 (m, 1H), 7.91 (br s, 1H), 7.88 (d, *J* = 8.8 Hz, 1H), 7.62 (br d, *J* = 7.9 Hz, 2H), 7.37–7.23 (m, 6H), 7.20–7.13 (m, 1H), 7.06 (br t, *J* = 6.0 Hz, 1H), 6.31 (br s, 1H), 5.50 (br s, 2H), 5.41 (br s, 0.5H), 5.34 (br s, 0.5H), 5.17–4.91 (m, 2H), 4.74 (br s, 0.5H), 4.63 (s, 0.5H), 4.49 (br d, *J* = 5.4 Hz, 1H), 4.46–4.36 (m, 2H), 4.33 (q, *J* = 7.2 Hz, 1H), 4.28 (d, *J* = 10.7 Hz, 0.6H), 4.25 (d, *J* = 10.9 Hz, 0.4H), 4.10–3.88 (m, 5.5H), 3.82 (dd, *J* = 16.8, 5.8 Hz, 1H), 3.78 (dd, *J* = 9.4, 2.3 Hz, 0.5H), 3.72–3.65 (m, 1H), 3.65–3.44 (m, 14.5H), 3.39 (t, *J* = 5.9 Hz, 2H), 3.32 (m, 1H), 3.28 (m, 1H), 3.25 (s, 1.5H), 3.23 (s, 1.5H), 3.20 (s, 1.5H), 3.18 (s, 1.5H), 3.14–3.08 (m, 3H), 3.07–3.02 (m, 0.5H), 3.02–2.92 (m, 3H), 2.88 (app d, *J* = 9.2 Hz, 1.5H), 2.84 (app d, *J* = 7.6 Hz, 1.5H), 2.43–2.38 (m, 1H), 2.31–2.25 (m, 1H), 2.21 (t, *J* = 13.8 Hz, 2H), 2.19–2.03 (m, 6H), 2.03–1.88 (m, 2H), 1.88–1.62 (m, 6H), 1.60–1.40 (m, 5H), 1.40–1.34 (m, 1H), 1.34–1.28 (m, 1H), 1.28–1.22 (m, 1H), 1.05 (d, *J* = 6.6 Hz, 1.5H), 1.01 (d, *J* = 6.7 Hz, 1.5H), 1.01 (d, *J* = 6.7 Hz, 1.5H) (two overlapping doublets), 0.98 (d, *J* = 6.7 Hz, 1.5H), 0.96 (d, *J* = 6.6 Hz, 1H), 0.90–0.72 (m, 18H). HRMS (ESI) calcd for C_81_H_125_N_12_O_22_^−^ [M-H]^−^ 1617.9037, found 1617.9048.

#### 3.2.17. Maleimide-PEG_4_-Glu-Gly-Cit-PABC-MMAE **20**

In a 2 mL polypropylene microcentrifuge tube, FmocNH-PEG_4_-Glu-Gly-Cit-PABC-MMAE 16 (25.5 mg, 0.015 mmol, 1 equiv.) was dissolved in dry DMF (0.2 mL), and piperidine (22 μL, 19 mg, 0.22 mmol, 15 equiv.) was added. The reaction mixture was stirred at 23 °C for 30 min, at which point TLC (silica gel, DCM–MeOH–AcOH, 3:1:0.04) indicated a full conversion of **16**. The product was precipitated portionwise in a 2 mL polypropylene microcentrifuge tube by the addition of Et_2_O (1.8 mL) to 80 μL aliquots of the reaction mixture, followed by centrifugation and supernatant removal. The combined precipitate was washed with Et_2_O (2 × 1.8 mL) with sonication and dried under a vacuum to afford crude NH_2_-PEG_4_-Glu-Gly-Cit-PABC-MMAE as a white solid; *R*_f_ 0.37 (silica gel, *n*-BuOH–H_2_O–AcOH, 3:1:1).

The crude NH_2_-PEG_4_-Glu-Gly-Cit-PABC-MMAE was dissolved in dry DMF (0.15 mL), and DIPEA (5.3 μL, 3.9 mg, 0.030 mmol, 2 equiv.) was added, followed by a solution of 3-maleimidopropionic acid Pfp ester **18** [40] (20.2 mg, 0.060 mmol, 4 equiv.) in dry DMF (0.1 mL). The reaction mixture was agitated on an orbital shaker at 23 °C for 30 min, at which point TLC (silica gel, *n*-BuOH–H_2_O–AcOH, 3:1:1) indicated a full conversion of NH_2_-PEG_4_-Glu-Gly-Cit-PABC-MMAE. The crude product was precipitated portionwise in a 2 mL polypropylene microcentrifuge tube by the addition of Et_2_O (1.8 mL) to 80 μL aliquots of the reaction mixture, followed by centrifugation and supernatant removal. The combined precipitate was washed with Et_2_O (1.8 mL) and dried under a vacuum. The resultant residue was purified by preparative TLC (silica gel, DCM–MeOH–AcOH, 3:1:0.04) to afford pure maleimide-PEG_4_-Glu-Gly-Cit-PABC-MMAE **20** (12.9 mg, 53%) as a yellowish glassy solid, which turned into off-white powder upon treatment with Et_2_O and drying under a vacuum. *R*_f_ 0.27 (silica gel, DCM–MeOH–AcOH, 3:1:0.04). ^1^H NMR (800 MHz, DMSO-*d*_6_, two rotamers) 10.47 (s, 0.5H), 10.43 (s, 0.5H), 8.96–8.87 (m, 1H), 8.64 (br t, *J* = 5.3 Hz, 1H), 8.33–8.23 (m, 2H), 8.10 (t, *J* = 5.7 Hz, 1H), 8.07–7.95 (m, 1H), 7.81 (d, *J* = 8.5 Hz, 0.5H), 7.65 (br s, 2H), 7.34–7.23 (m, 6H), 7.34–7.23 (m, 6H), 7.19–7.12 (m, 1H), 7.03 (br s, 1H), 6.99 (s, 2H), 5.73 (br s, 2H), 5.16–4.90 (m, 2H), 4.74 (s, 0.5H), 4.63 (s, 0.5H), 4.50 (d, *J* = 5.6 Hz, 0.5H), 4.50–4.47 (s, 0.5H), 4.46 (d, *J* = 6.1 Hz, 0.5H), 4.43–4.38 (m, 0.5H), 4.34–4.29 (m, 2H), 4.27 (d, *J* = 10.8 Hz, 0.5H), 4.25 (d, *J* = 10.9 Hz, 0.5H), 4.04–3.87 (m, 5H), 3.84 (dm, *J* = 16.2 Hz, 1H), 3.78 (dd, *J* = 9.3, 2.3 Hz, 0.5H), 3.65–3.45 (m, 15H), 3.36 (t, *J* = 5.9 Hz, 2H), 3.34 (dd, *J* = 5.4, 3.8 Hz, 0.5H), 3.25 (s, 1.5H), 3.23 (s, 1.5H), 3.20 (s, 1.5H), 3.18 (s, 1.5H), 3.14 (q, *J* = 5.8 Hz, 2H), 3.11 (br s, 2H), 3.08–3.03 (m, 0.5H), 3.00–2.94 (s, 3H), 2.93–2.89 (m, 1H), 2.87 (br s, 1.5H), 2.84 (br s, 1.5H), 2.43–2.38 (m, 1H), 2.31–2.24 (m, 1H), 2.29–2.13 (m, 1H), 2.13–2.07 (m, 1H), 2.07–2.02 (m, 2H), 2.02–1.92 (m, 1H), 1.92–1.65 (m, 6H), 1.60–1.45 (m, 3H), 1.45–1.35 (m, 1H), 1.35–1.28 (m, 1H), 1.28–1.22 (m, 1H), 1.05 (d, *J* = 6.7 Hz, 1.5H), 1.01 (d, *J* = 6.7 Hz, 1.5H), 0.99 (d, *J* = 6.6 Hz, 1.5H), 0.96 (d, *J* = 6.6 Hz, 1.5H), 0.90–0.72 (m, 18H). HRMS (ESI) calcd for C_77_H_121_N_13_O_23_^2+^ [M+2H]^2+^ 797.9344, found 797.9344.

### 3.3. Enzymatic Reactions

#### 3.3.1. Trastuzumab Deglycosylation

To a solution of trastuzumab (12.7 mg, 480 μL, 26.3 mg/mL) in PBS (pH 8.4, 150 mM NaCl) was added PNGase F (1000 U, 2 μL, 500,000 U/mL). The resultant mixture was gently agitated on an orbital shaker at 37 °C for 48 h. As PAGE indicated the complete removal of glycans, the mixture was concentrated to 180 μL using a 50,000 MWCO centrifugal concentrator. The mixture was then loaded onto a Bio-Gel P-100 column (V_total_ = 5 mL) equilibrated in a PBS (pH 7.4, 150 mM NaCl). Elution with the PBS yielded deglycosylated trastuzumab (7 mg, 310 μL, 22.5 mg/mL, main fraction).

#### 3.3.2. Trastuzumab-N_3_-I

To deglycosylated trastuzumab (300 μg, 13.3 μL, C = 22.5 mg/mL) in a PBS buffer of pH 7.4, 2 μL (200 equiv.) of azido linker **5** (200 mM solution in water) were added. The resulting solution was gently mixed. To it, 12.5 μL of a 40% (*w*/*v*) solution of an MTGase formulation (1% *w*/*w* in dextran) in a PBS buffer of pH 7.4 (final enzyme concentration 0.08% *w*/*v*) and 34.7 μL of a PBS buffer of pH 7.4 were added. The resulting solution was gently mixed and left for 48 h in a dark place at room temperature without stirring. After the time elapsed, white precipitation was observed. The reaction mixture was centrifuged, and the supernatant was collected and purified by gel filtration on Bio-Gel P-100. The resulting antibody solution was concentrated to the desired concentration using centrifuge concentrators.

#### 3.3.3. Trastuzumab-N_3_-II

To deglycosylated trastuzumab (300 μg, 20.7 μL, C = 14.4 mg/mL) in a PBS buffer of pH 7.4, 4 μL (400 equiv.) of azido linker **7** (200 mM solution in water) were added. The resulting solution was gently mixed. To it, 10 μL of a 40% (*w*/*v*) solution of an MTGase formulation (1% *w*/*w* in dextran) in a PBS buffer of pH 7.4 (final enzyme concentration 0.08% *w*/*v*) and 15.3 μL of a PBS buffer of pH 7.4 were added. The resulting solution was gently mixed and left for 48 h in a dark place at room temperature without stirring. After the time elapsed, white precipitation was observed. The reaction mixture was centrifuged, and the supernatant was collected and purified by gel filtration on Bio-Gel P-100. The resulting antibody solution was concentrated to the desired concentration using centrifuge concentrators.

#### 3.3.4. Trastuzumab-N_3_-III

To deglycosylated trastuzumab (300 μg, 13.3 μL, C = 22.5 mg/mL) in a PBS buffer of pH 7.4, 57.4 μL of a PBS buffer of pH 7.4 and 25 μL (400 equiv.) of azido linker **9** (32 mM solution in water/DMSO 1:1 *v*/*v*) were added. The resulting solution was gently mixed. To it, 23.9 μL of a 40% (*w*/*v*) solution of an MTGase formulation (1% *w*/*w* in dextran) in a PBS buffer of pH 7.4 (final enzyme concentration 0.08% *w*/*v*) were added. The resulting solution was gently mixed and left for 48 h in a dark place at room temperature without stirring. After the time elapsed, white precipitation was observed. The reaction mixture was centrifuged, and the supernatant was collected and purified by gel filtration on Bio-Gel P-100. The resulting antibody solution was concentrated to the desired concentration using centrifuge concentrators.

### 3.4. Synthesis of ADCs

#### 3.4.1. ADC **I**

To trastuzumab-N_3_-I (240 μg, 120 μL, C = 2 mg/mL) in a PBS buffer of pH 7.4, 1.35 μL (10 equiv.) of BCN-PEG_4_-Glu-Gly-Cit-PABC-MMAE **19** (12.2 mM solution in DMSO) were added. The resulting solution was gently mixed and left for 3 h at room temperature with gentle agitation on an orbital shaker. After the time elapsed, white precipitation was observed. The reaction mixture was centrifuged, and the supernatant was collected and purified by gel filtration on Bio-Gel P-100.

#### 3.4.2. ADC **II**

To trastuzumab-N_3_-II (200 μg, 116 μL, C = 1.72 mg/mL) in a PBS buffer of pH 7.4, 3.4 μL (30 equiv.) of BCN-PEG_4_-Glu-Gly-Cit-PABC-MMAE **19** (12.2 mM solution in DMSO) was added. The resulting solution was gently mixed and left for 3 h at room temperature with gentle agitation on an orbital shaker. After the time elapsed, white precipitation was observed. The reaction mixture was centrifuged, and the supernatant was collected and purified by gel filtration on Bio-Gel P-100.

#### 3.4.3. ADC **III**

To trastuzumab-N_3_-III (193 μg, 470 μL, C = 0.41 mg/mL) in a PBS buffer of pH 7.4, 3.3 μL (30 equiv.) of BCN-PEG_4_-Glu-Gly-Cit-PABC-MMAE **19** (12.2 mM solution in DMSO) were added. The resulting solution was gently mixed and left for 3 h at room temperature with gentle agitation on an orbital shaker. After the time elapsed, white precipitation was observed. The reaction mixture was centrifuged, and the supernatant was collected and purified by gel filtration on Bio-Gel P-100.

#### 3.4.4. ADC **IV**

In a 500 μL microcentrifuge tube, to a solution of trastuzumab (408 μg, 100 μL, 4.08 mg/mL) in a PBS/EDTA (pH 6.4, 150 mM NaCl, 10 mM EDTA) was added a solution of tris (2-carboxyethyl) phosphine hydrochloride (TCEP-HCl) in a PBS/EDTA, pH 6.4 (5.63 μL, 2.5 mM, 5.0 equiv.). The resulting mixture was vortexed and briefly centrifuged at 1000 g, after which the microcentrifuge tube was sealed with Parafilm and gently agitated on an orbital shaker at 37 °C for 3 h. The mixture was then cooled to 23 °C over the course of 7 min, and a solution of maleimide-PEG_4_-Glu-Gly-Cit-PABC-MMAE **20** in DMSO (11.8 μL, 3.56 mM, 15 equiv., 10% *v*/*v* DMSO in the final mixture) was added. The resulting mixture was vortexed and briefly centrifuged at 1000 g, and the tube was resealed with Parafilm and gently agitated on an orbital shaker at 23 °C for 1 h. The mixture was then loaded onto a column packed with Sephadex G-50 Fine (V_total_ = 5 mL) equilibrated in a PBS (pH 7.4, 150 mM NaCl). Elution with PBS yielded ADC **IV** (main fraction, 0.349 mg, 410 μL, 0.85 mg/mL, 86%, average DAR = 6.1 by UV, 5.4 by HIC). The next two 200 μL fractions were checked spectrophotometrically for the absence of a free payload.

### 3.5. PAGE Analysis

Denaturing gel electrophoresis for the conjugate analysis was performed using the standard Laemmli SDS-PAGE procedure in a Tris-glycine buffer [44]. A 15% density separating gel was used. The staining was performed with the Coumassie Blue dye, followed by washing the gel with 10% aqueous acetic acid.

### 3.6. Hydrophobic Interaction Chromatography (HIC)

The analysis was performed on an Agilent 1100 instrument (Agilent Technologies) equipped with a quaternary pump and a diode array detector using a Thermo MabPac^TM^ HIC-Butyl 5 μm 4.6 × 100 mm column (Thermo Fisher Scientific) thermostatted at 35 °C. Eluent A comprised 2 M aqueous ammonium sulfate containing 50 mM of a sodium phosphate buffer (pH 7.0), and eluent B comprised 50 mM of a sodium phosphate buffer (pH 7.0) containing 20% (*v*/*v*) of HPLC grade *i*-PrOH (Merck). Solutions of ADCs **I**–**IV** in PBS (pH 7.4) were concentrated using 50,000 MWCO centrifugal concentrators (ADC **II** was supplemented with 0.01% *v*/*v* Tween 80 before concentration) at 4 °C and injected for analysis (injection volume: 100 µL; ADCs **I**–**III**: 70 μg, ADC **IV**: 100 μg). The sample was eluted with a linear gradient from 0% B to 100% B over 20 min, followed by 5 min of 100% B (flow rate: 1 mL/min). UV absorbance was detected at 280 nm.

### 3.7. DAR Determination

The drug-to-antibody ratio of the ADCs **I**–**IV** was determined spectrophotometrically as reported previously [45,46,47]. The formula for the DAR calculation is as follows:

$$DAR=\frac{\varepsilon_{248}^{Ab}-F\varepsilon_{280}^{Ab}}{F\varepsilon_{280}^{D}-\varepsilon_{248}^{D}}$$

where *F* = *A*_248_/*A*_280_ (values derived from the UV spectrum of the conjugate), *Ab* = trastuzumab, *D* = R-Glu-Gly-Cit-PABC-MMAE, and the extinction coefficients employed are listed in Table 2.

### 3.8. ELISA

The recombinant extracellular domain of human HER2 (amino acid residues 23–649) was produced in HEK293F cells. The binding of trastuzumab and trastuzumab-based ADCs to HER2 was estimated by ELISA. Briefly, 96-well MaxiSorp plates (Nunc, Thermo Fisher Scientific) were coated with 100 µL of HER2-ECD protein or polyclonal goat anti-human IgG (whole molecule) antibodies (Cat# I1886, Sigma Aldrich, Merck) at a concentration of 1 µg/mL in a PBS overnight at 4 °C. Subsequently, the plates were washed five times with 300 µL of the wash buffer (phosphate buffered saline (PBS) with 0.05% Tween-20 [PBST]) and blocked with 250 µL of PBST containing 0.1% sodium caseinate for 1 h at room temperature to avoid non-specific binding. Trastuzumab and ADCs were diluted with the conjugate buffer (PBST with 0.225% sodium caseinate) to the concentration of 1000 ng/mL, followed by seven 1:3 serial dilutions. Then, 100 μL of samples were added into the plate both to HER2-ECD or whole anti-human IgG in duplicate and incubated for 1 h at 37 °C. Next, the plates were washed with PBST five times. Polyclonal goat anti-human Fc horseradish peroxidase (HRP)-conjugated antibodies (Cat# A0170, Sigma Aldrich, Merck) were diluted 1:60,000 with the conjugate buffer, and 100 µL was added to each well, followed by incubation for 1 h at 37 °C. After washing, 100 µL tetramethyl benzidine (TMB) was added into the plate and incubated for 15 min in the dark. The reaction was stopped with 100 µL of 10% phosphate acid. Absorbance at 450 nm was measured by a Varioscan LUX plate reader (Thermo Fisher Scientific). The trastuzumab and trastuzumab-based ADC concentrations were adjusted according to the values obtained by using an anti-human IgG concentration control. All experiments were performed in triplicate. The concentration dependence of the binding of trastuzumab and the conjugates was estimated by OD_450_ values using GraphPad Prism 10 software.

### 3.9. Cell Viability Assay

#### 3.9.1. Preparation of Solutions for Colorimetric Assessment of Metabolic Activity of Cells (MTT)

To prepare the MTT solution, a dry sample of thiazolyl blue tetrazolium bromide was dissolved in phosphate-buffered saline, pH = 7.4 (DPBS) to a concentration of 5 mg/mL. The resulting solution was sterile filtered through a filter with a pore diameter of 0.2 μm, and the filtrate was collected in a sterile container protected from light.

To prepare the solubilizing solution, 40% *v*/*v* dimethylformamide (DMF) was diluted in 2% (vol.) glacial acetic acid. To the resulting solution, 16% *w*/*v* sodium dodecyl sulfate (SDS) was added, and the pH of the solution was adjusted to 4.7 using 2% *v*/*v* glacial acetic acid.

#### 3.9.2. Viability Analysis of MDA-MB-231 and BT-474 Cell Lines (Cytotoxicity Assessment)

Cytotoxicity was assessed on MDA-MB-231 and BT-474 cells. Cell line BT474 was cultured in an Advanced RPMI-1640 (Gibco, Thermo Fisher Scientific) medium with 10% FBS, 1% Glutamax, and 1× Antibiotic/Antimycotic Solution (Capricorn Scientific, Ebsdorfergrund, Germany). Cell line MDA-MB-231 was cultured in an Advanced DMEM (Gibco, Thermo Fisher Scientific) medium with 10% FBS, 1% Glutamax, and 1× Antibiotic/Antimycotic Solution (Capricorn Scientific). The cell cultures were obtained from ATCC and passaged 7 times. Cell confluency was 85% before the MTT test.

ADCs **I**–**IV** were stored in PBS (pH 7.4) at 4 °C. Their concentrations were determined spectrophotometrically by measuring absorbance at 280 nm and using the molar absorption coefficients listed in Table 2. Twofold dilutions of the test compounds in DPBS were prepared as a negative control in the culture medium. A series of final concentrations of twenty twofold dilutions began at 100 nM. Incubation was carried out in a humidified atmosphere at 37 °C with 8% CO_2_ for 72 h.

Cell viability was determined by the colorimetric assessment of the metabolic activity of the cells (MTT). The analysis was carried out after incubation for 72 h with 85% confluency. All the measurements were carried out in three biological replicates. Then, 10 μL of MTT solution were added to the test samples per well to a final concentration of 0.45 mg/mL. Incubation was carried out for 1 h at 37 °C. Then, 100 μL of solubilizing solution were added to each well to dissolve formazan crystals. Optical absorption was measured using a Varioskan multimodal reader (Thermo Fisher Scientific) at 570 nm. The obtained results were analyzed using MS Excel 2013 software. Based on the MTT test results, a graph of the dependence of the average survival rate on the concentration of the test substance was constructed on a logarithmic scale. A well with cells that did not contain the test compounds was taken as 100% viability. Relative survival was calculated using the following formula: (absorption for a given well)/(absorption for a well without adding the drug). The cytotoxic concentration for each compound (IC_50_) was determined from the obtained inverse sigmoid as the concentration value at which a cytotoxic effect on 50% of the cells in the monolayer was induced.

## 4. Conclusions

We synthesized two branched linkers with spacers of different lengths, differentiated by a PEG_4_ fragment. These linkers were conjugated to trastuzumab in a site-specific MTGase-catalyzed reaction, and the resulting azide-bearing antibodies were further functionalized with the EGCit cleavable linker and monomethyl auristatin E to yield homogeneous DAR 6 ADCs. These conjugates, along with a homogeneous DAR 2 ADC and a heterogeneous DAR 6 conjugate with the same cleavable linker that were obtained by the modification of the reduced interchain disulfide bonds with maleimide, were tested for HER2 binding affinity and cytotoxicity. The conjugates showed an unusual difference in cytotoxicity, with complete preservation of their affinity to the target. In particular, the conjugate with the short, branched linker exhibited an approximately tenfold lower activity (0.68 nM) than the conjugate with the long, branched linker (0.074 nM), whose potency was identical to that of the DAR-matched heterogeneous conjugate (0.071 nM).

Presumably, the reason for the low in vitro efficacy of the “short” DAR 6 conjugate is poor drug release from the cleavable linker by lysosomal enzymes, since the branched core with the short spacer creates steric hindrance. Therefore, choosing the correct spacer length for a branched linker is extremely important. To date, no such studies have been performed for branched ADC linkers. The structure–activity relationship pattern we have found is of interest and will be further investigated.

## Figures and Tables

**Table 1:** IC_50_ of conjugates on BT-474 cells.

Conjugate	IC_50,_ nM
ADC **I**	0.35 ± 0.03
ADC **II**	0.68 ± 0.09
ADC **III**	0.074 ± 0.013
ADC **IV**	0.071 ± 0.011

**Table 2:** Molar absorbance coefficients of the conjugate components.

	ε_280,_ M^−1^⋅cm^−1^	ε_248,_ M^−1^⋅cm^−1^
Trastuzumab	225,000	90,800
R-Glu-Gly-Cit-PABC-MMAE	1500	15,900

## Data Availability

Data contained within the article and Supplementary Materials.

## Supplementary Materials

The following supporting information can be downloaded at https://www.mdpi.com/article/10.3390/ijms252413356/s1.
