# Supplementary material for: Branched Linkers for Homogeneous Antibody-Drug Conjugates: How Long Is Long Enough?
Source: Int J Mol Sci. 2024 Dec 12;25(24):13356. doi: 10.3390/ijms252413356 (PMC11678271; doi:10.3390/ijms252413356)

Supporting Information

Table of Contents

Full structures of amino linkers and payloads reported in the literature S2

NMR spectra S5

HRMS spectra S54

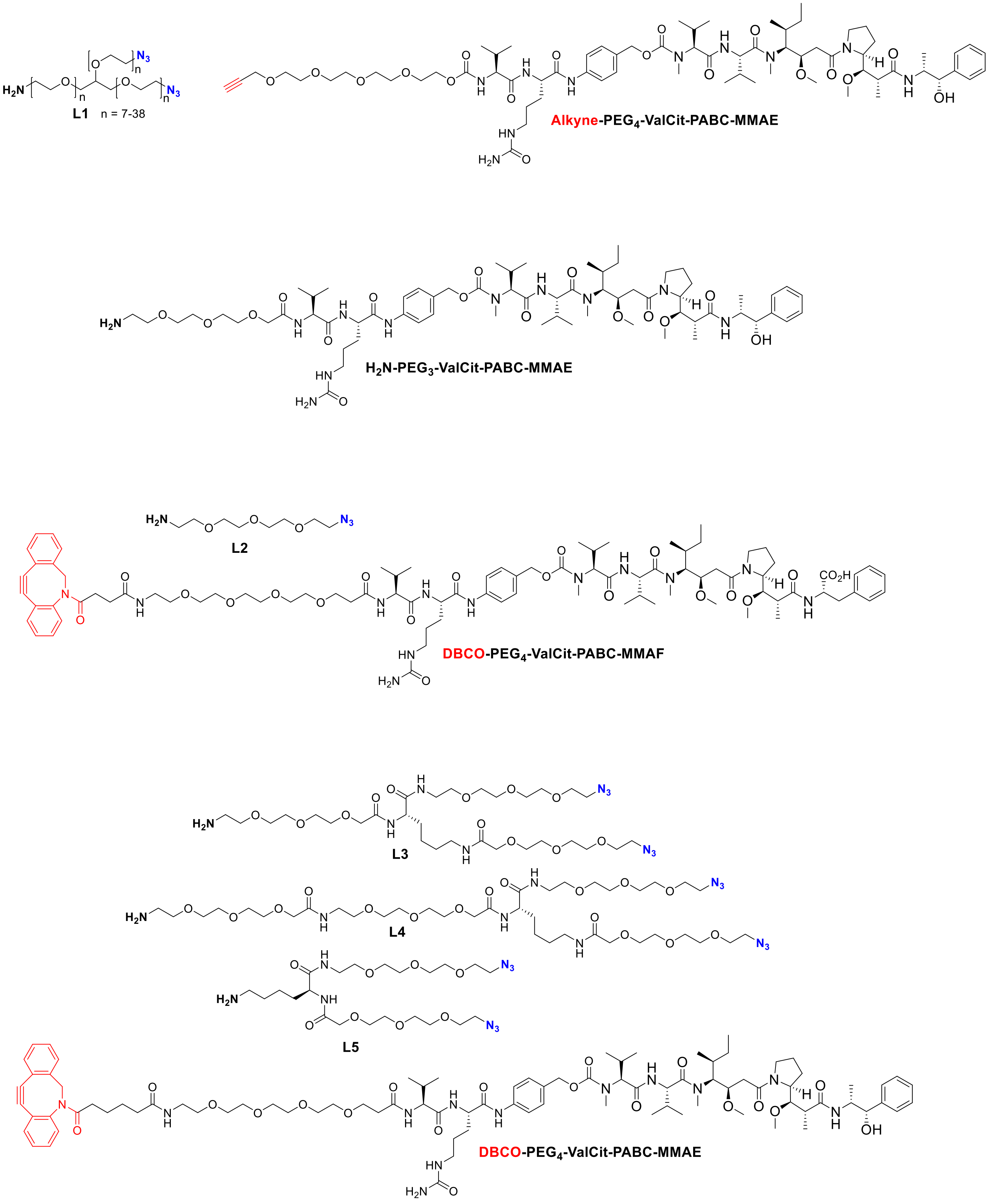

Figure S1. *Cont.*

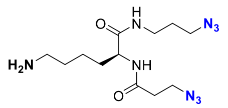

L6

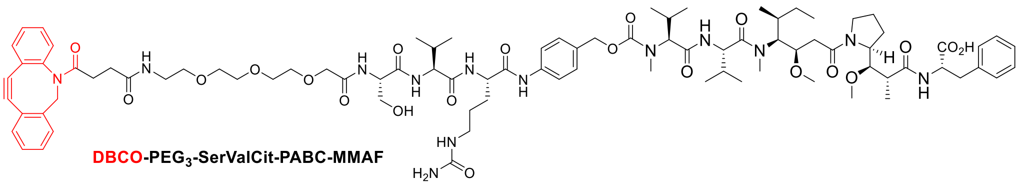

DBCO-PEG$_3$-SerValCit-PABC-MMAF

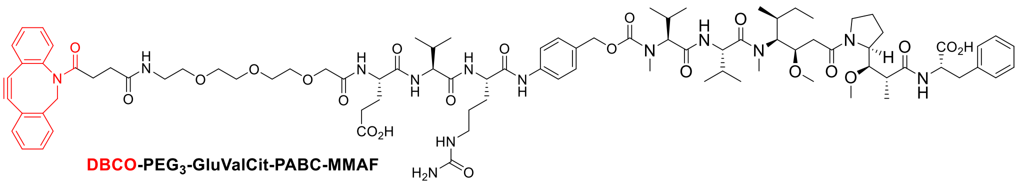

DBCO-PEG$_3$-GluValCit-PABC-MMAF

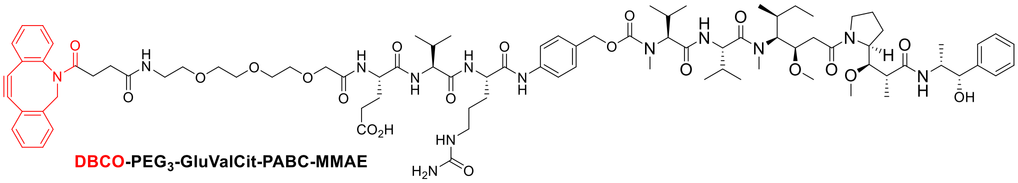

DBCO-PEG$_3$-GluValCit-PABC-MMAE

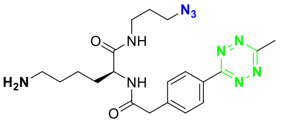

L7

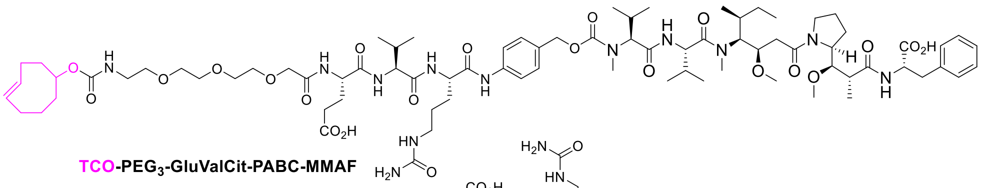

TCO-PEG$_3$-GluValCit-PABC-MMAF

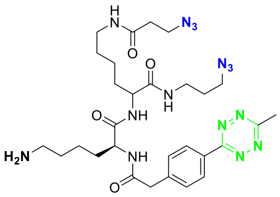

L8

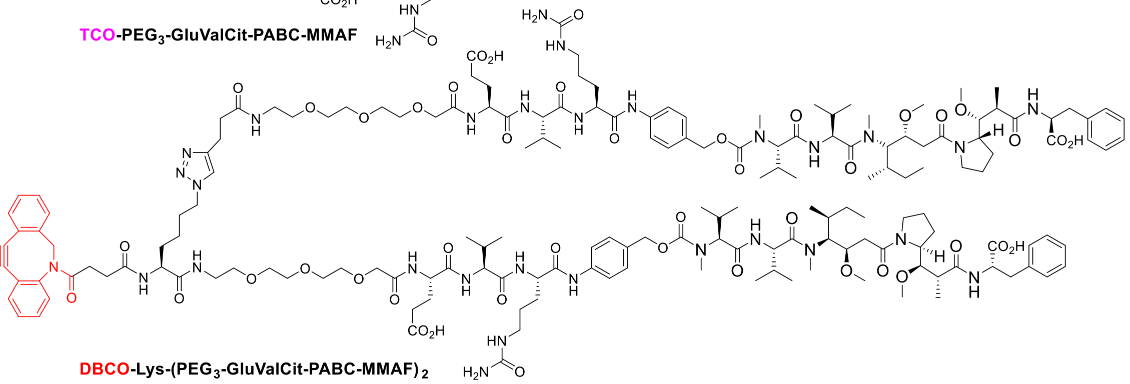

DBCO-Lys-(PEG$_3$-GluValCit-PABC-MMAF)$_2$

Figure S1. *Cont.*

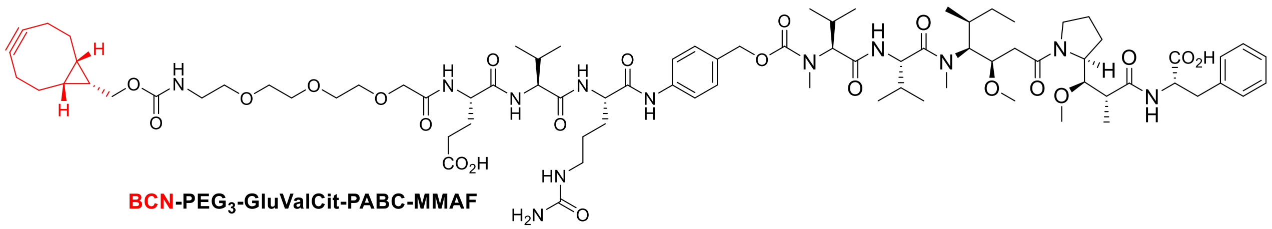

BCN-PEG$_3$-GluValCit-PABC-MMAF

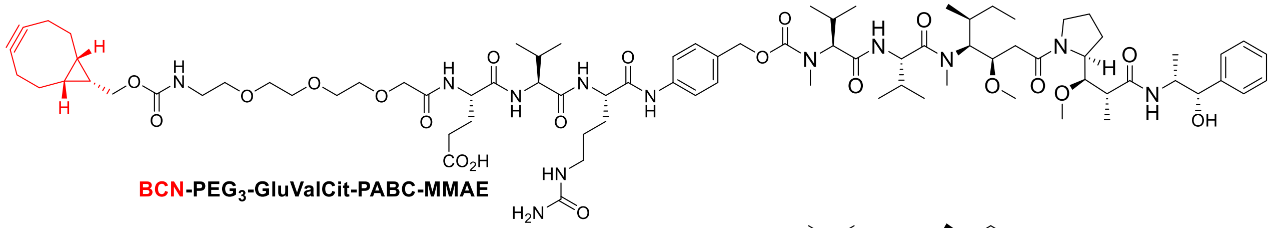

BCN-PEG$_3$-GluValCit-PABC-MMAE

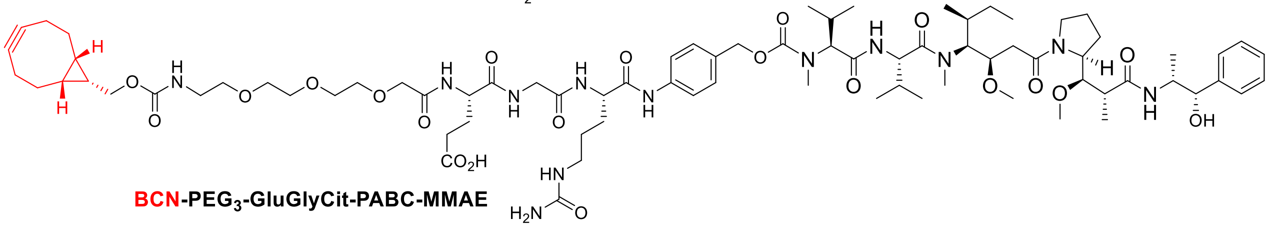

BCN-PEG$_3$-GluGlyCit-PABC-MMAE

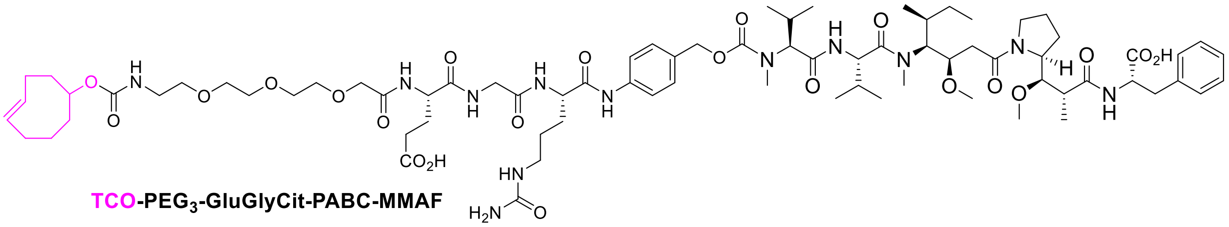

TCO-PEG$_3$-GluGlyCit-PABC-MMAF

Figure S1. Full structures of amino linkers and functionalized payload derivatives as reported in the literature on ADC synthesis MTGase-mediated antibody modification (see Figure 5 in the main text).

File S1.

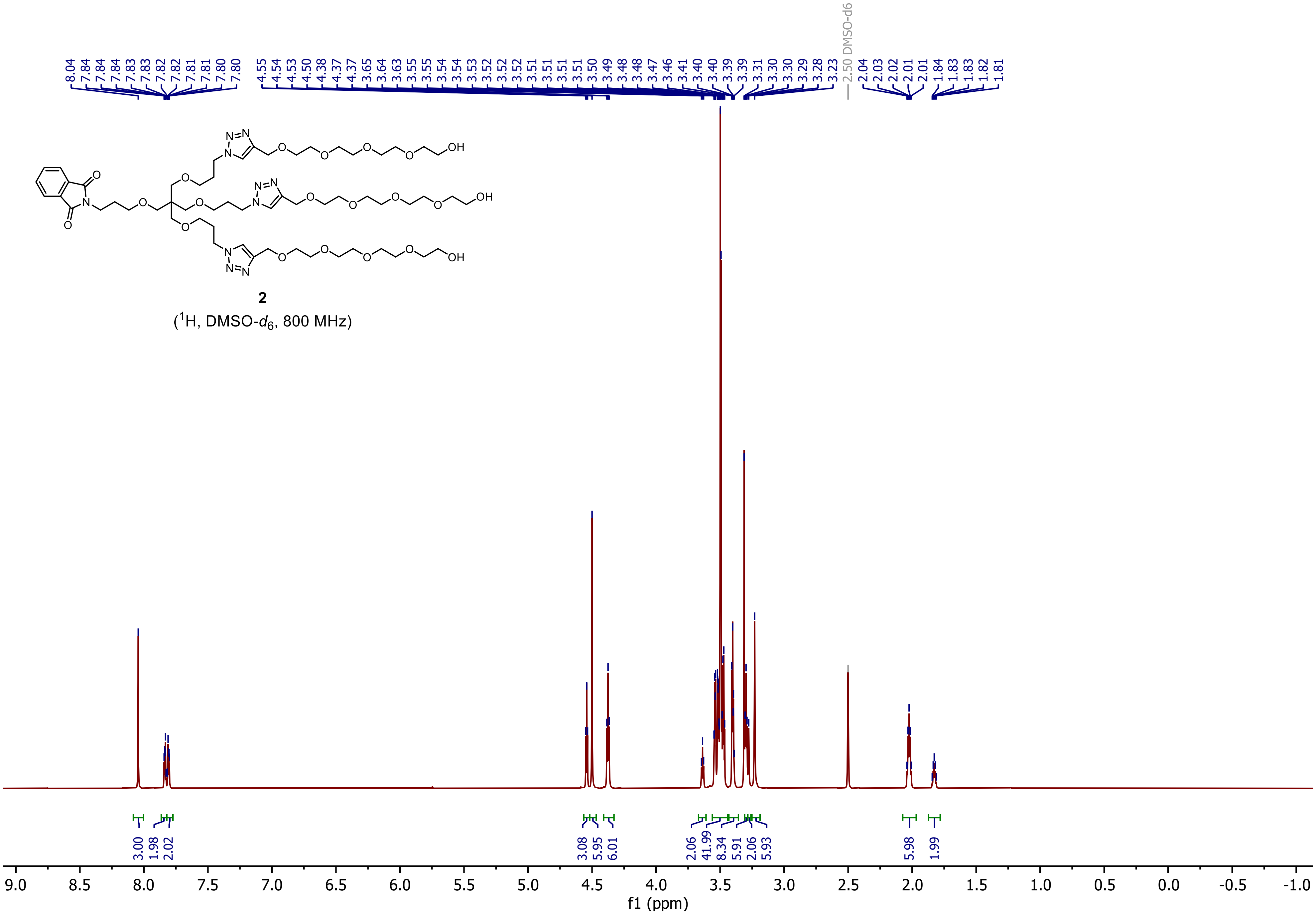

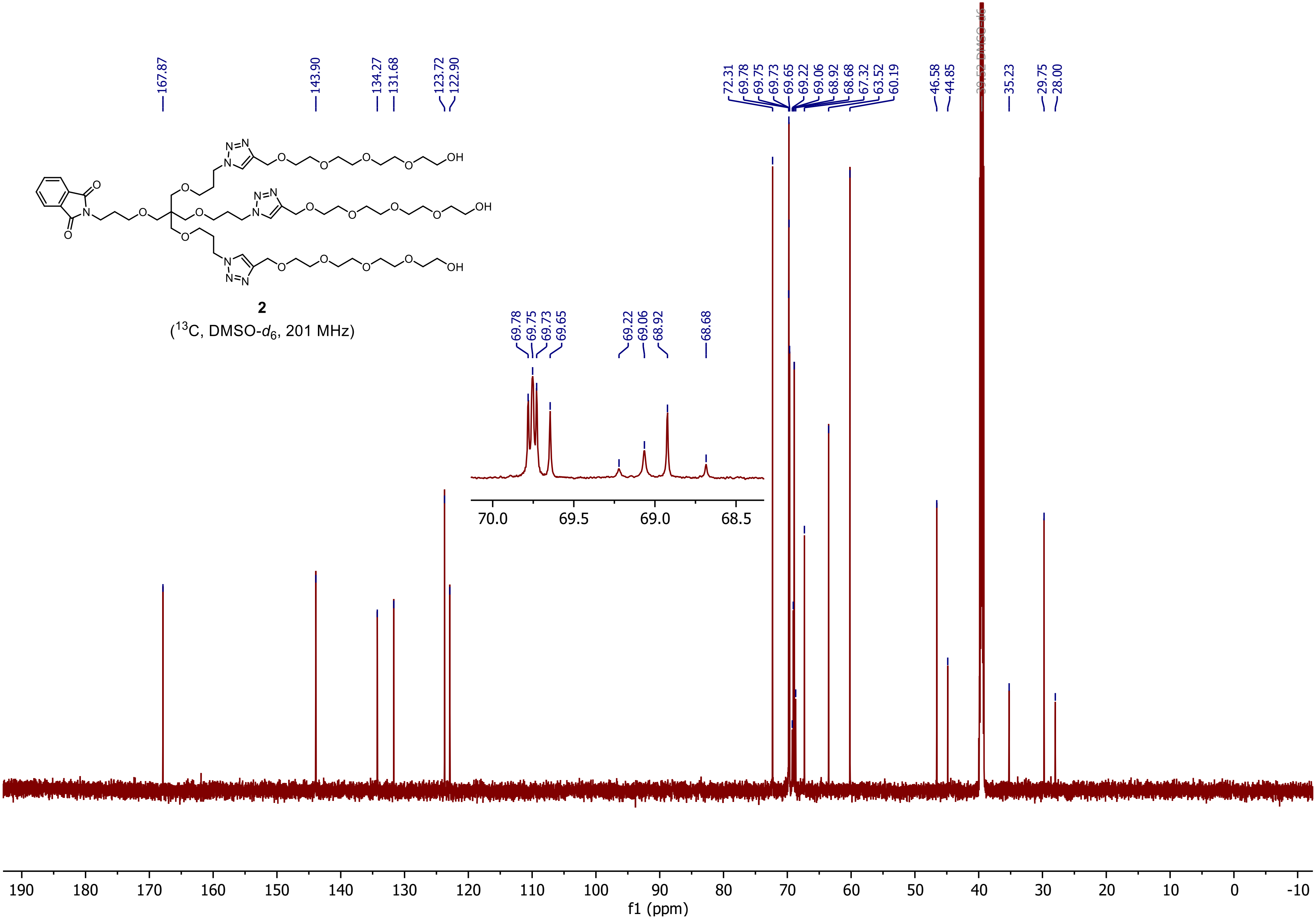

167.87
143.90
134.27
131.68
123.72
122.90
72.31
69.78
69.75
69.73
69.65
69.22
69.06
68.92
68.68
67.32
63.52
60.19
46.58
44.85
39.52 DMSO-d6
35.23
29.75
28.00
OH
OH
OH
2
(^{13}C, DMSO-d_6, 201 MHz)
70.0
69.5
69.0
68.5
190 180 170 160 150 140 130 120 110 100 90 80 70 60 50 40 30 20 10 0 -10
f1 (ppm)

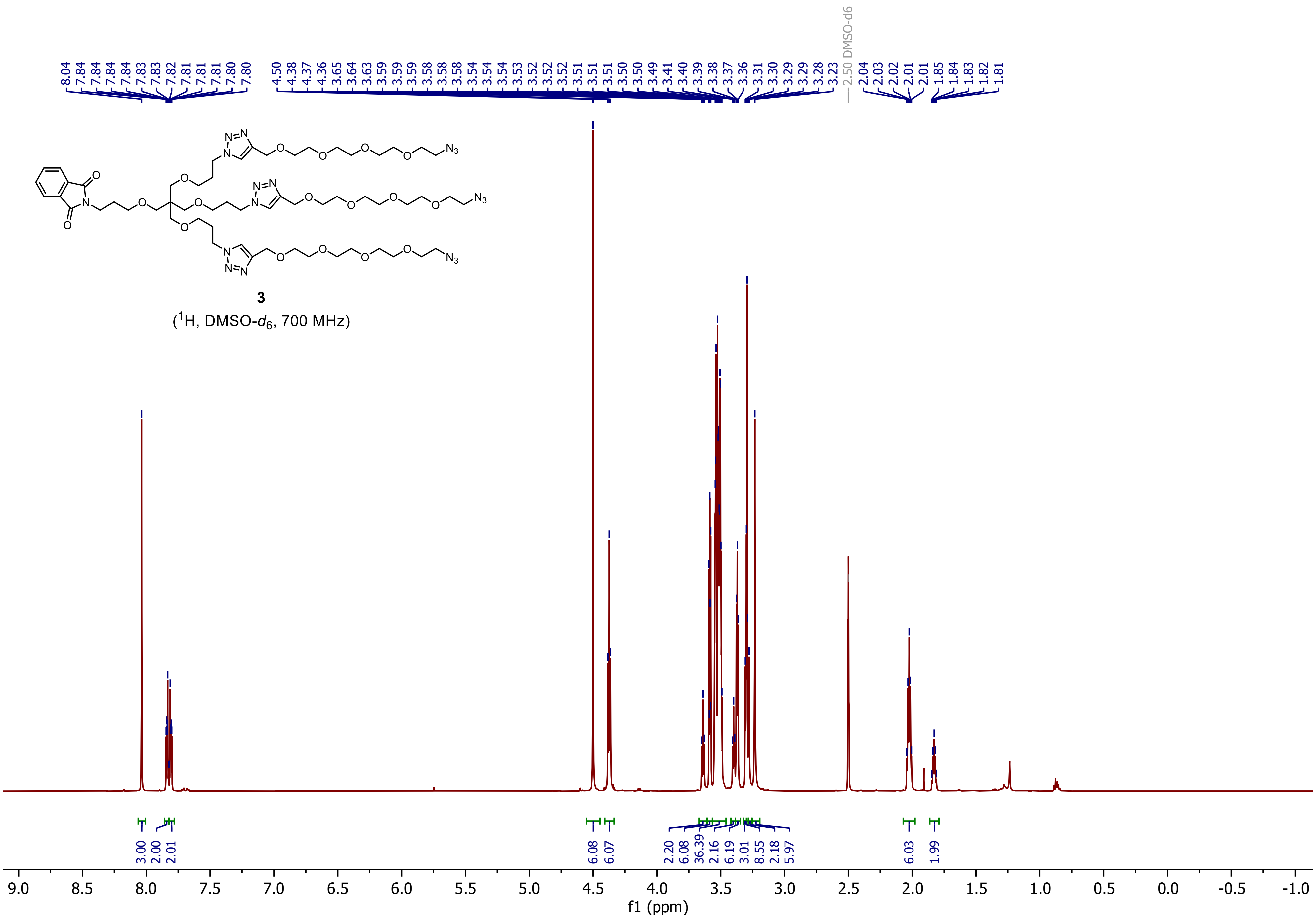

3

(^{1}H, DMSO-d_6, 700 MHz)

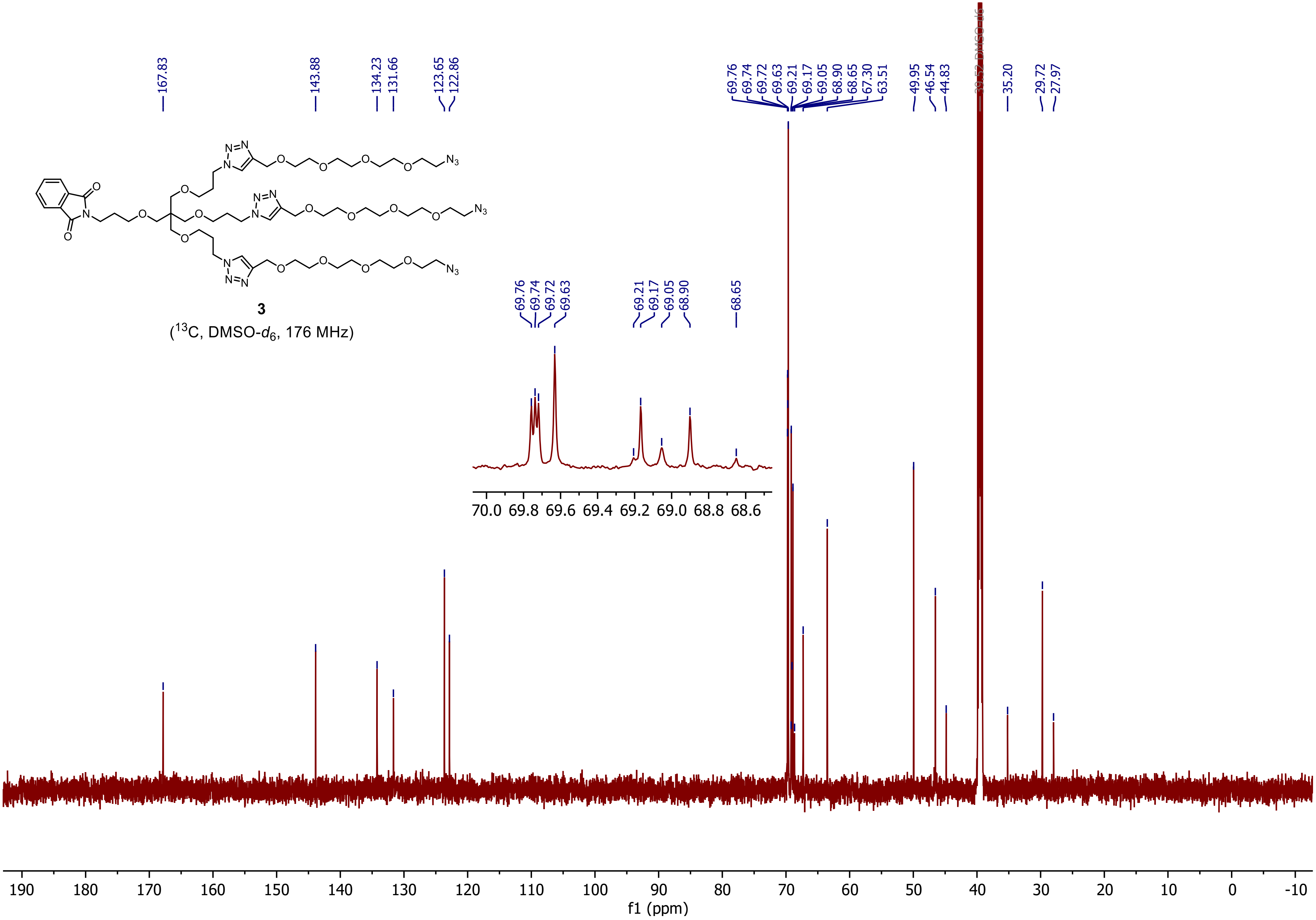

167.83
143.88
134.23
131.66
123.65
122.86
69.76
69.74
69.72
69.63
69.21
69.17
69.05
68.90
68.65
67.30
63.51
49.95
46.54
44.83
39.52 DMSO-d6
35.20
29.72
27.97
N3
3
(^{13}C, DMSO-d_6, 176 MHz)
70.0 69.8 69.6 69.4 69.2 69.0 68.8 68.6
190 180 170 160 150 140 130 120 110 100 90 80 70 60 50 40 30 20 10 0 -10
f1 (ppm)

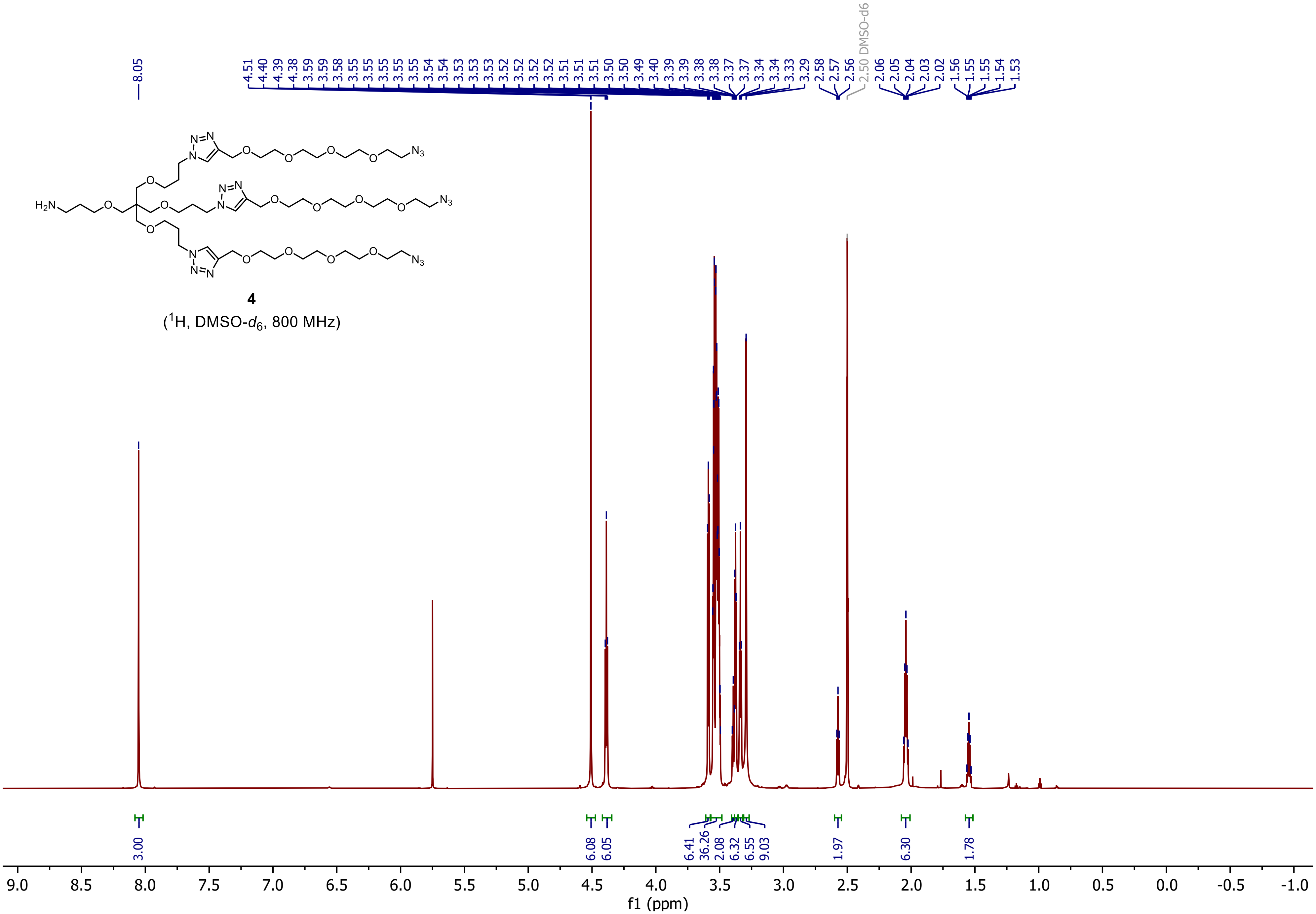

4

(^{1}H, DMSO-d_6, 800 MHz)

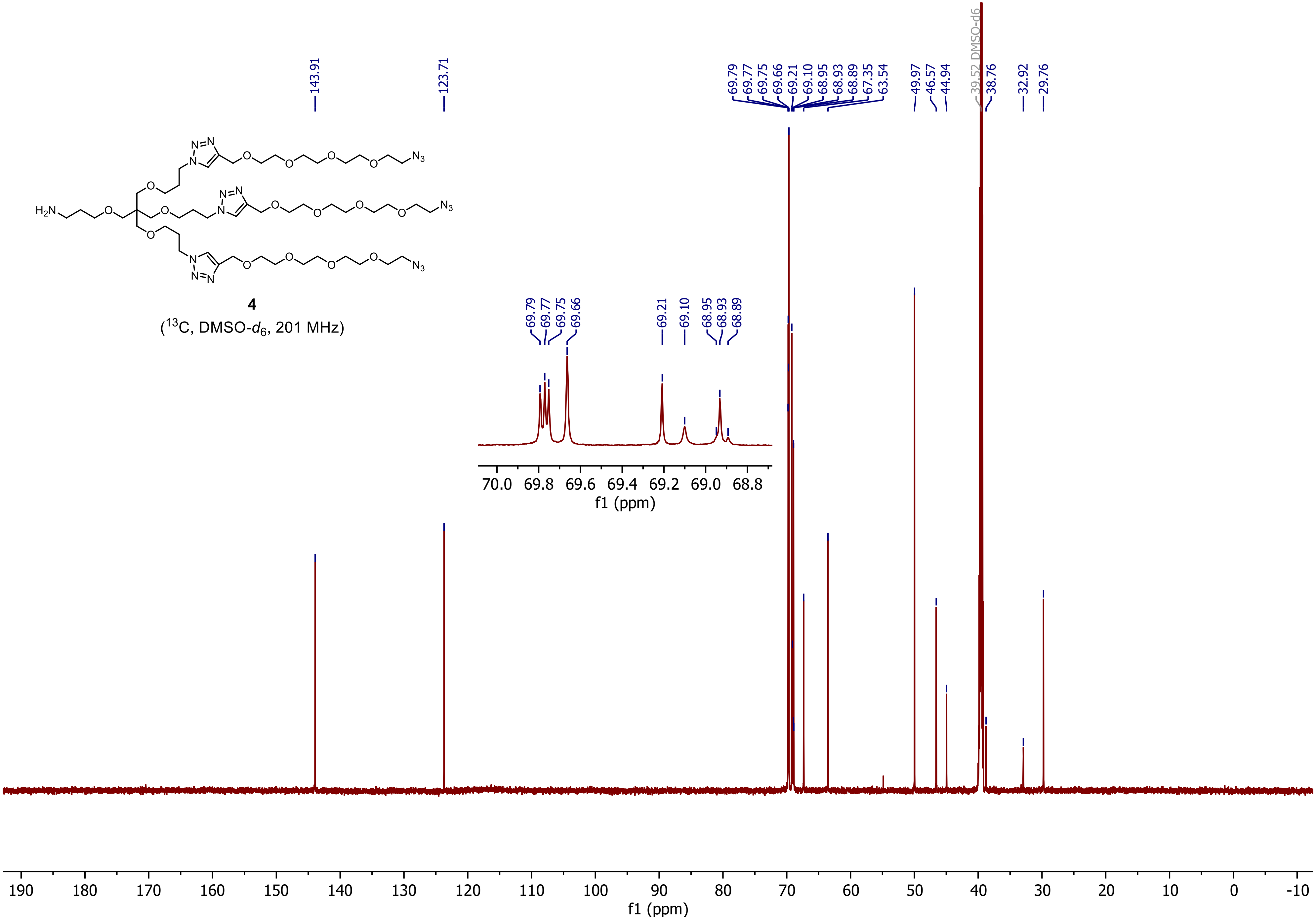

4

(^{13}C, DMSO-d_6, 201 MHz)

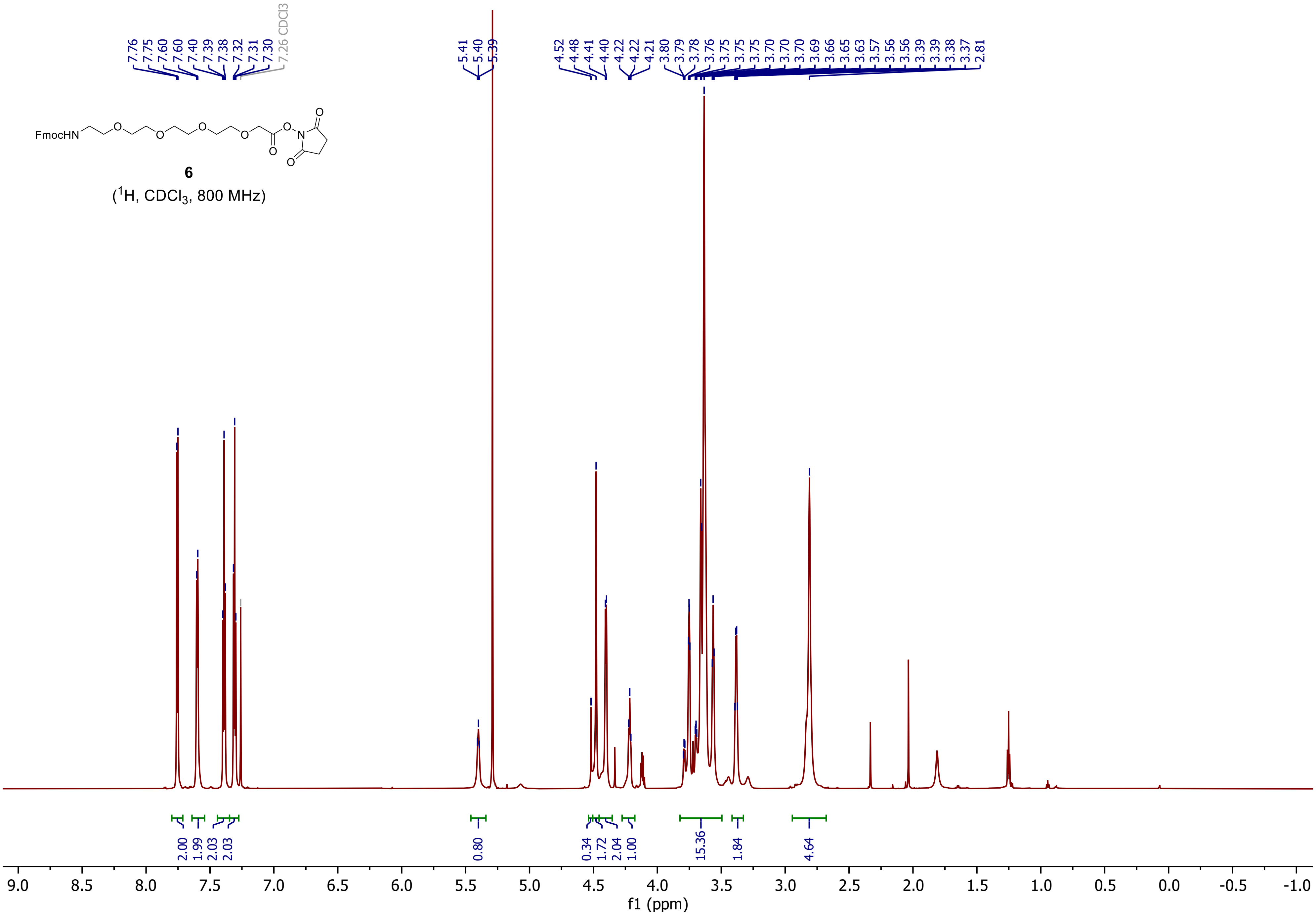
FmocHN
O
6
(1H, CDCl3, 800 MHz)
7.26 CDCl3
f1 (ppm)

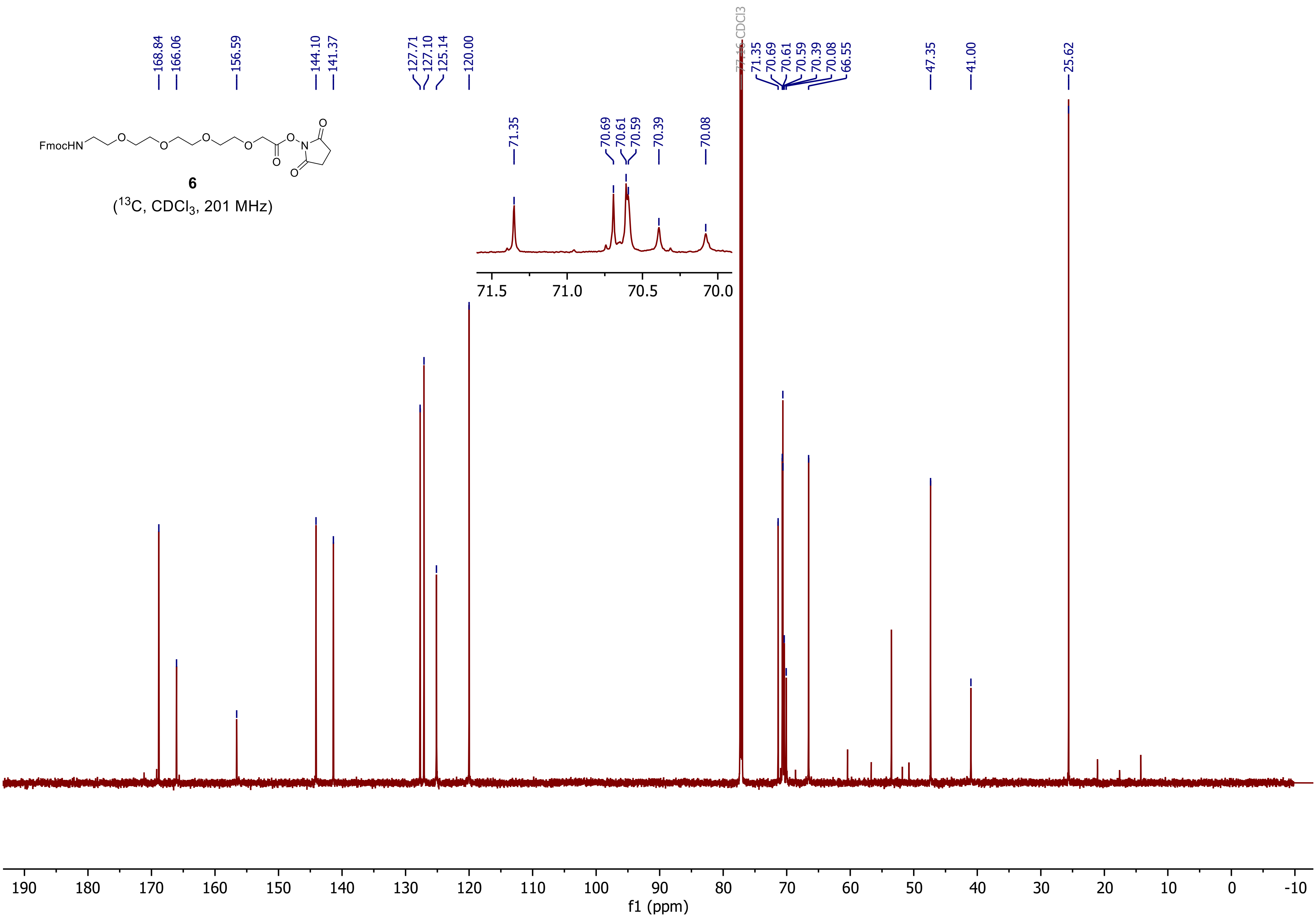

FmocHN
6
(^{13}C, CDCl$_3$, 201 MHz)
168.84
166.06
156.59
144.10
141.37
127.71
127.10
125.14
120.00
77.16 CDCl3
71.35
70.69
70.61
70.59
70.39
70.08
66.55
47.35
41.00
25.62
71.5
71.0
70.5
70.0
f1 (ppm)

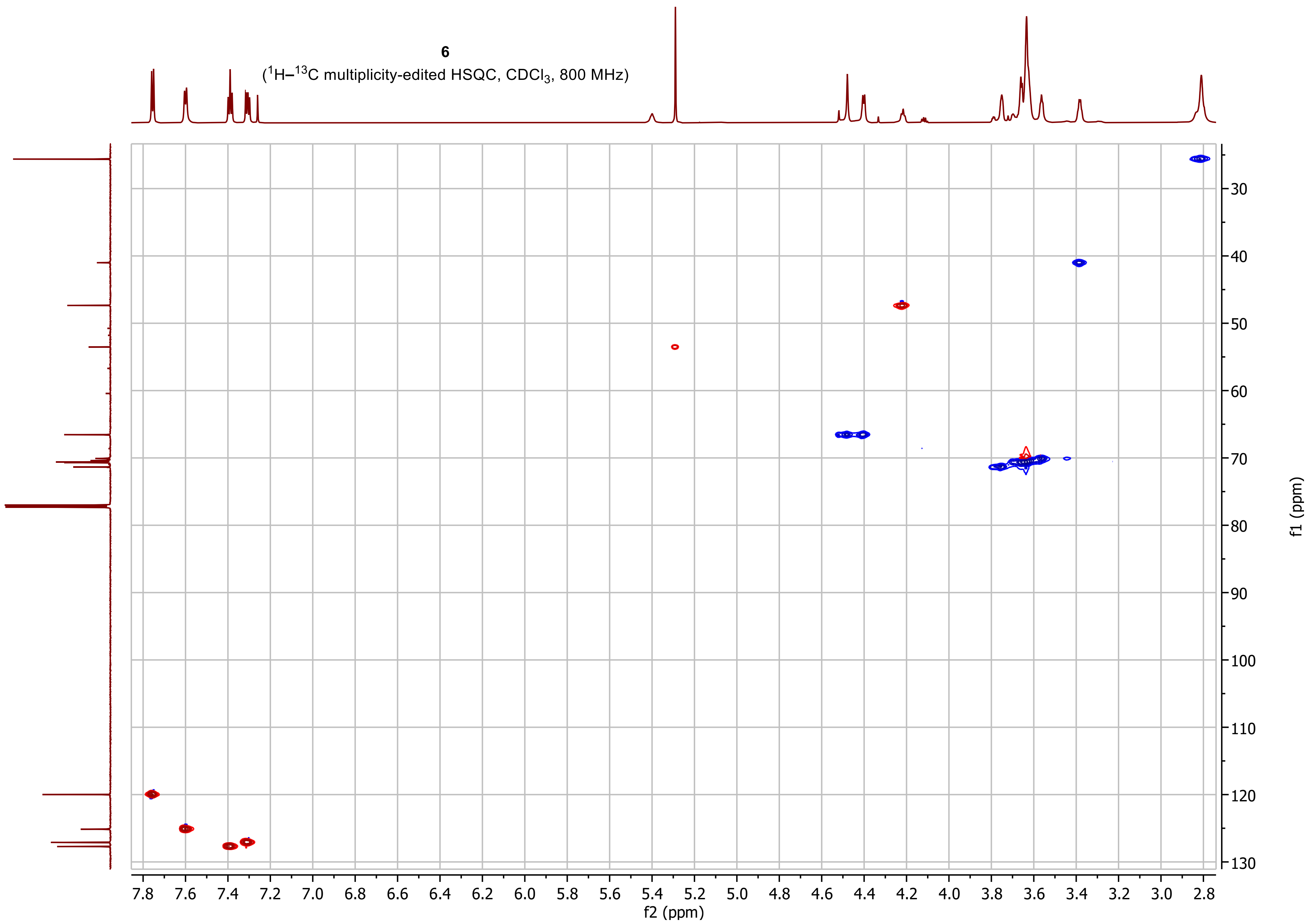

6
(^{1}H–^{13}C multiplicity-edited HSQC, CDCl$_3$, 800 MHz)
f2 (ppm)
f1 (ppm)

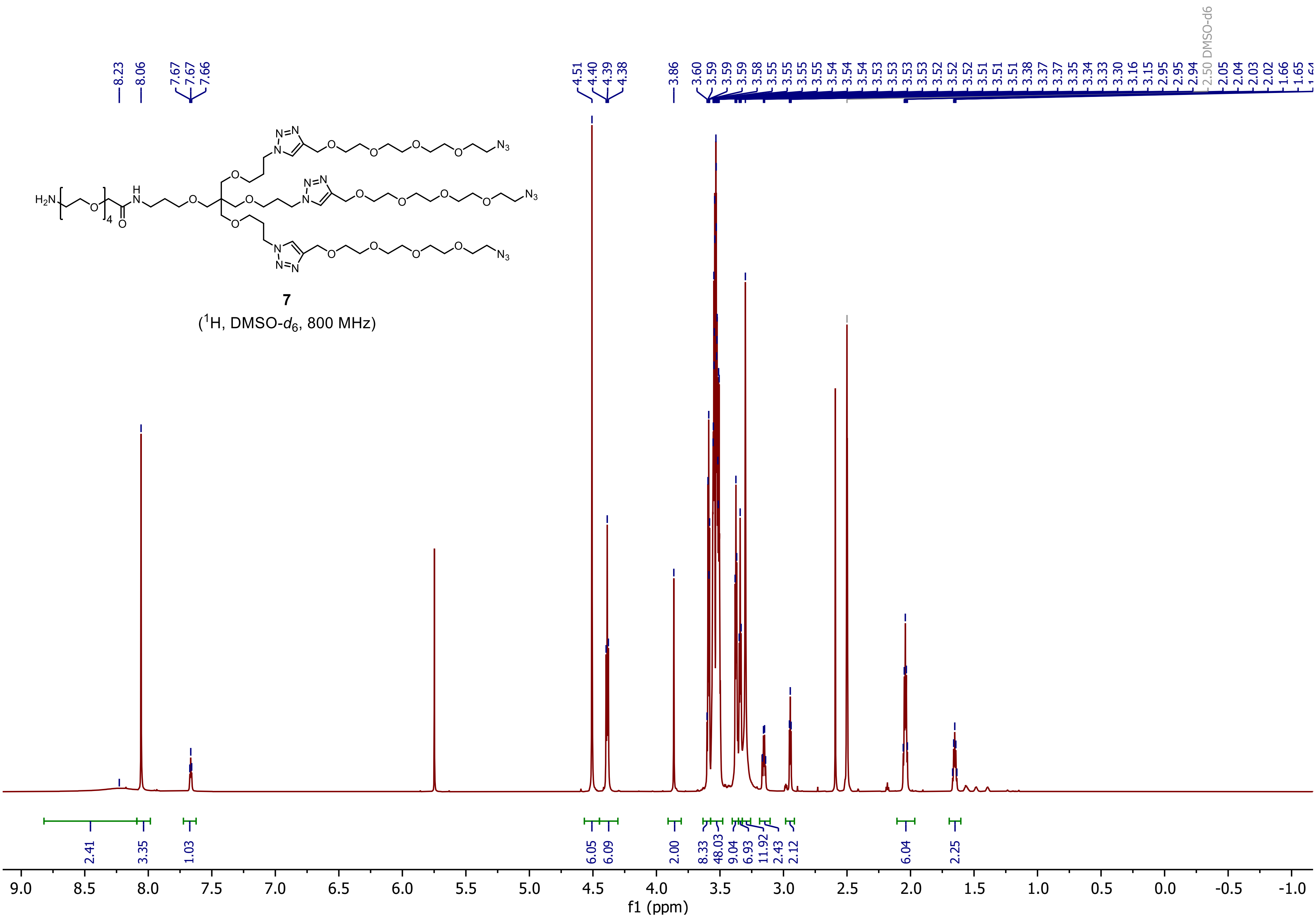

7

(^{1}H, DMSO-d_6, 800 MHz)

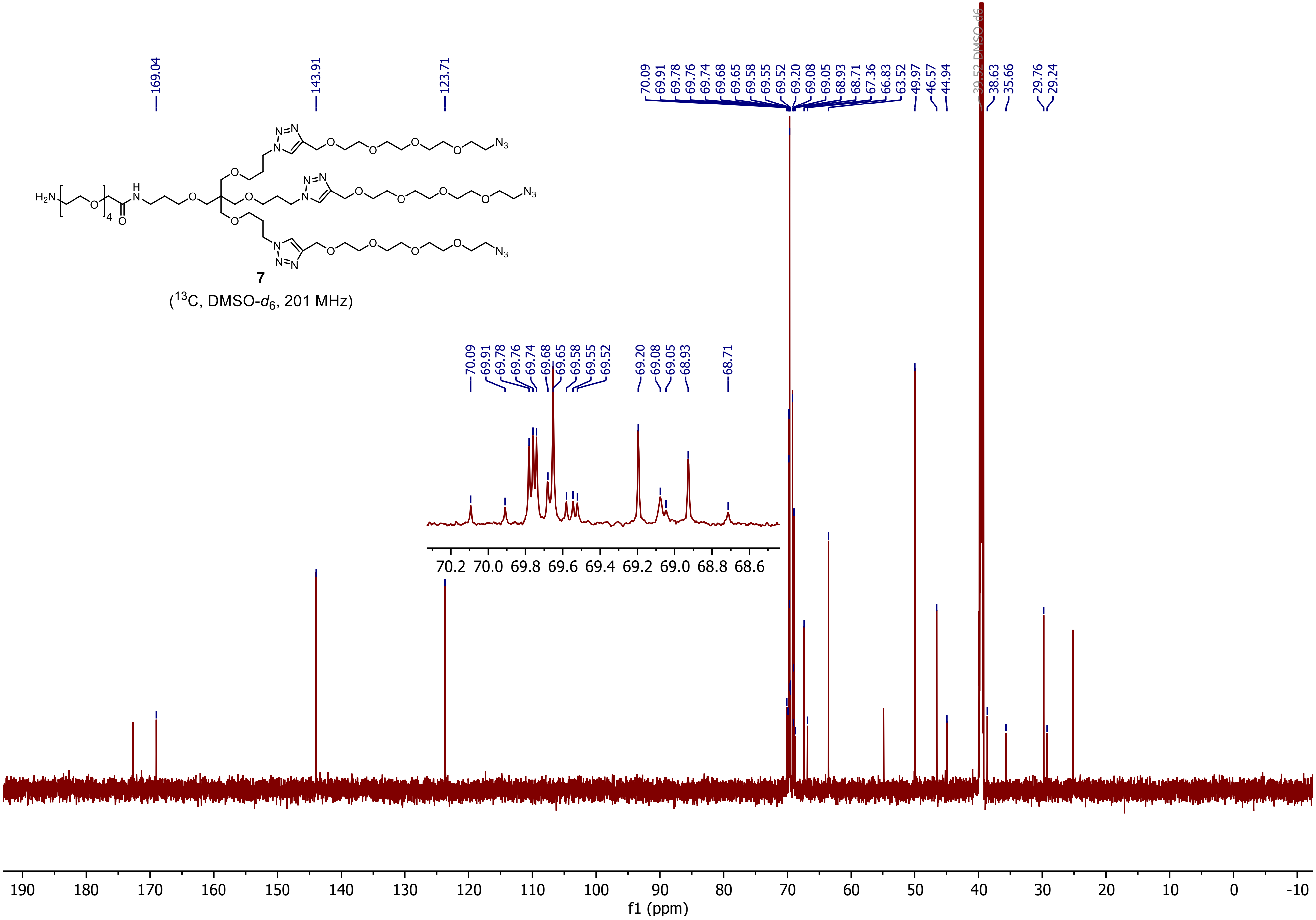

7

(^{13}C, DMSO-d_6, 201 MHz)

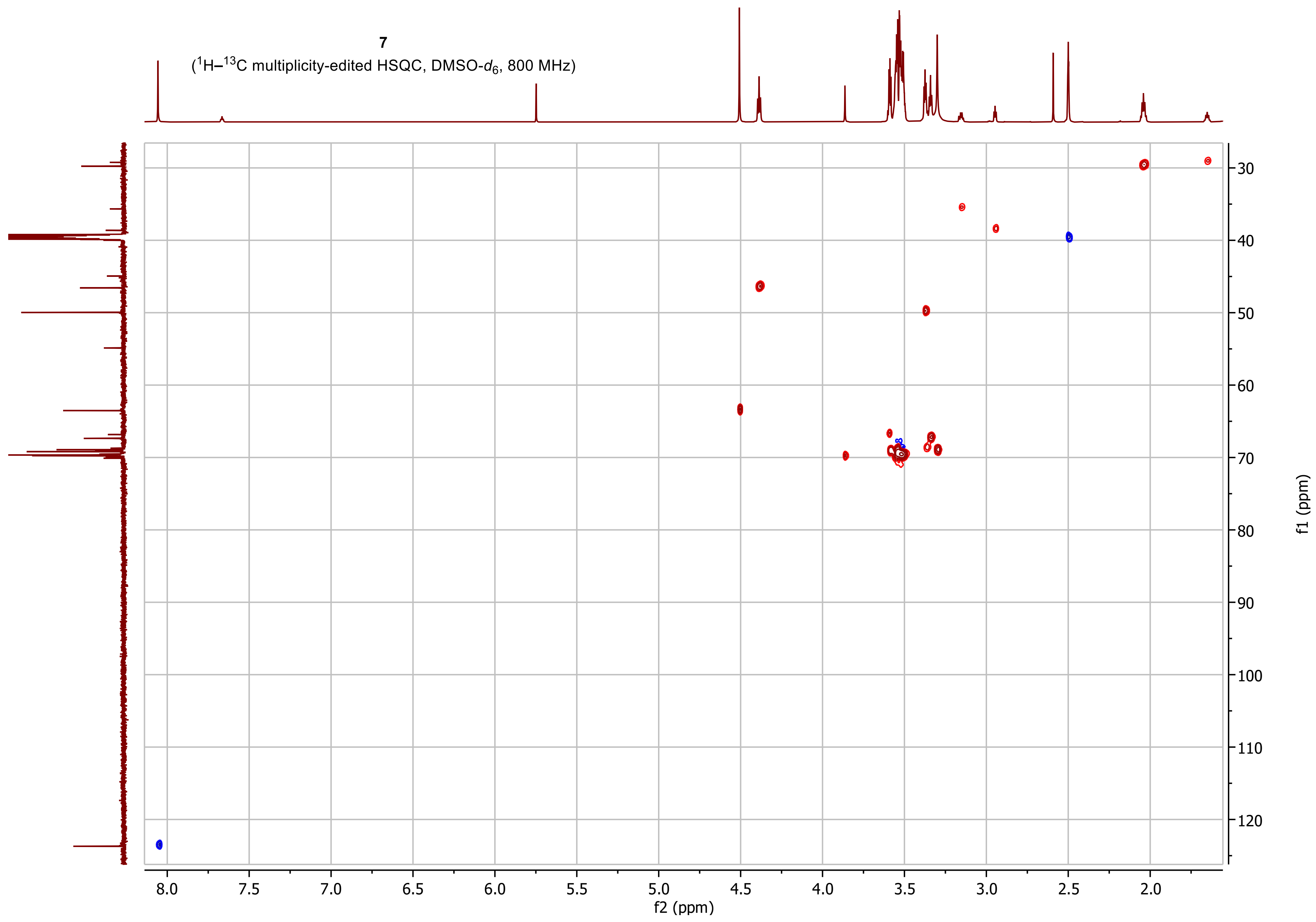
7
(^{1}H–^{13}C multiplicity-edited HSQC, DMSO-d_6, 800 MHz)
8.0
7.5
7.0
6.5
6.0
5.5
5.0
4.5
4.0
3.5
3.0
2.5
2.0
f2 (ppm)
30
40
50
60
70
80
90
100
110
120
f1 (ppm)

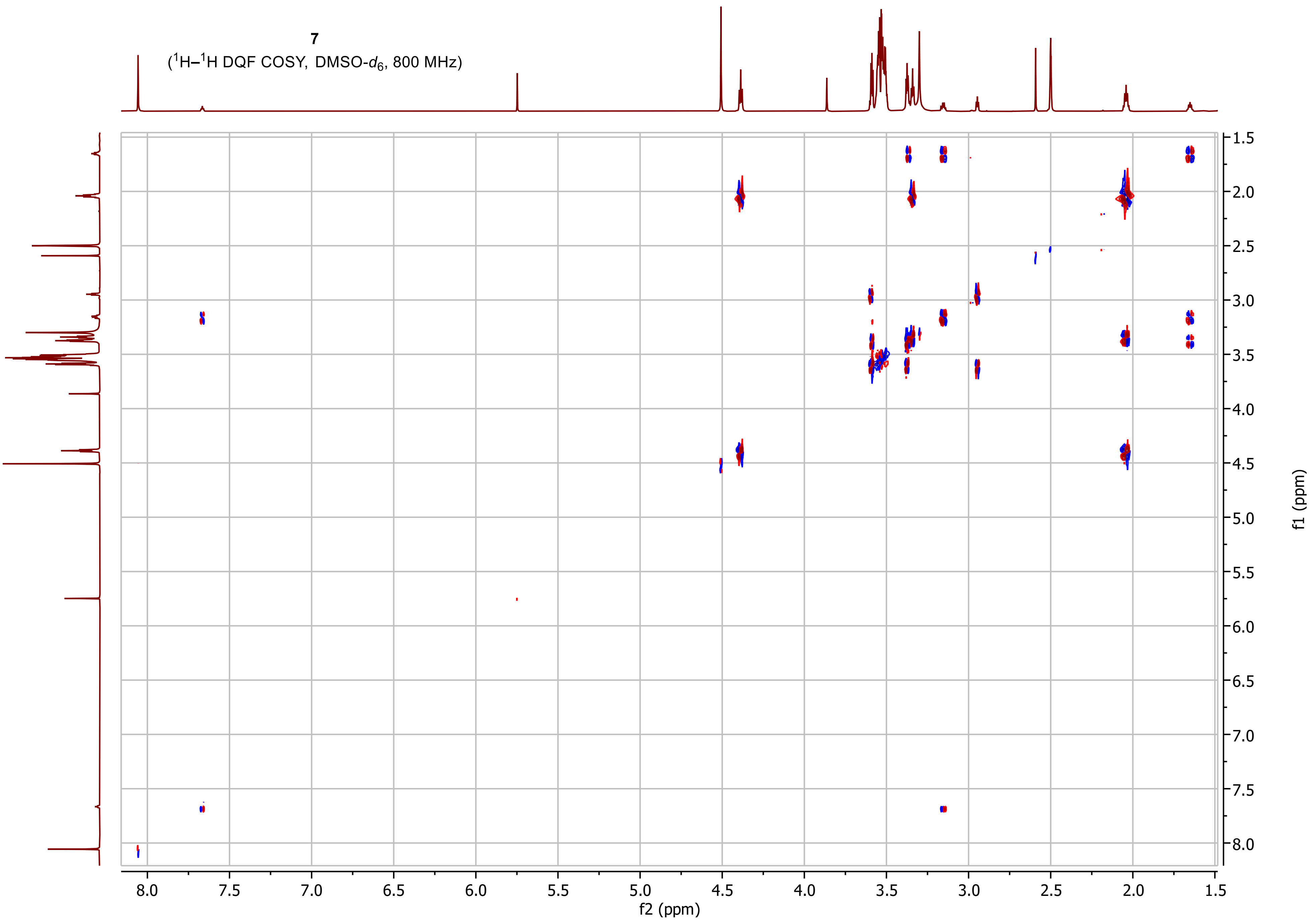
7
(^{1}H–^{1}H DQF COSY, DMSO-d_6, 800 MHz)
f2 (ppm)
f1 (ppm)

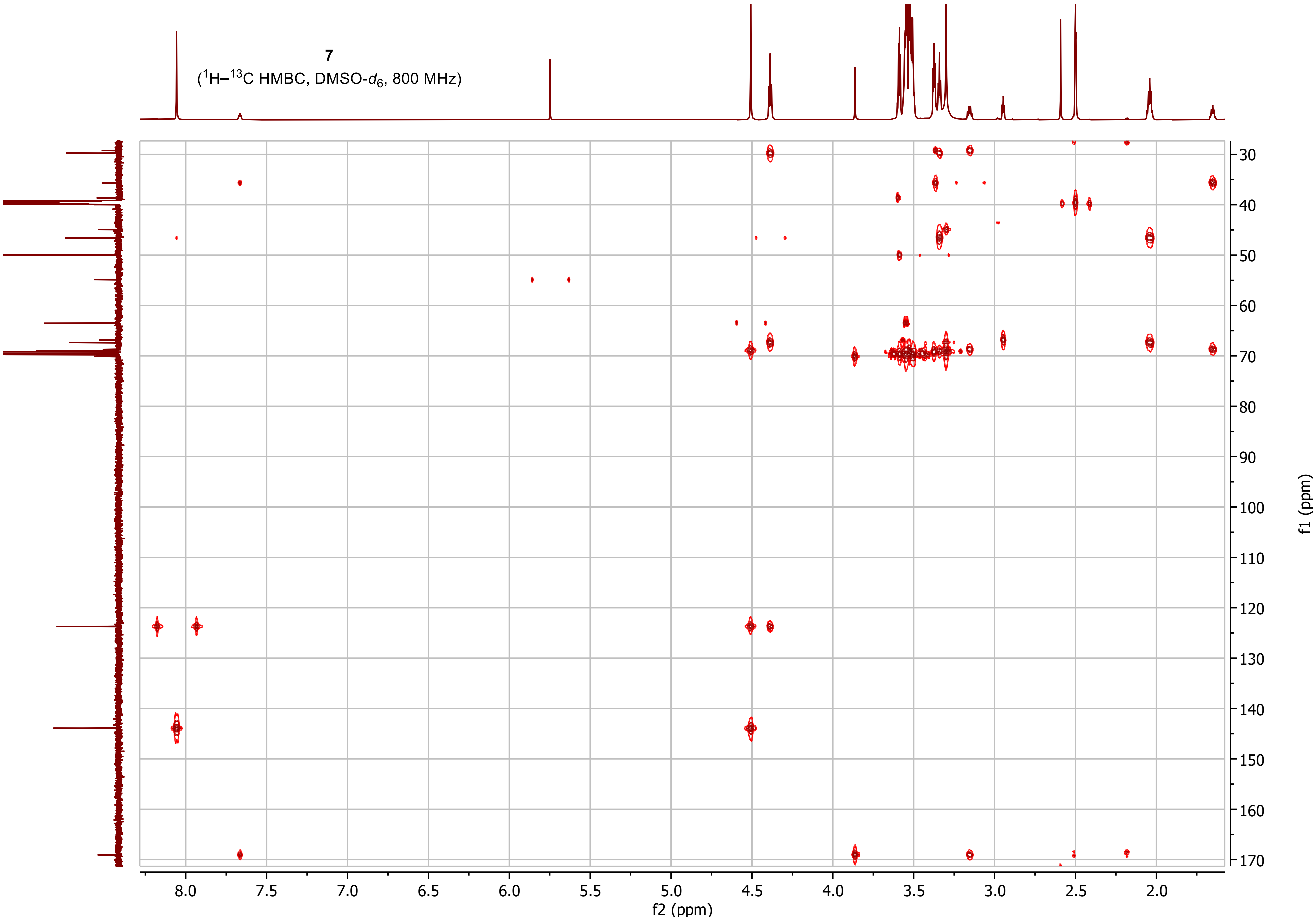
7
(^{1}H–^{13}C HMBC, DMSO-d_6, 800 MHz)
f2 (ppm)
f1 (ppm)

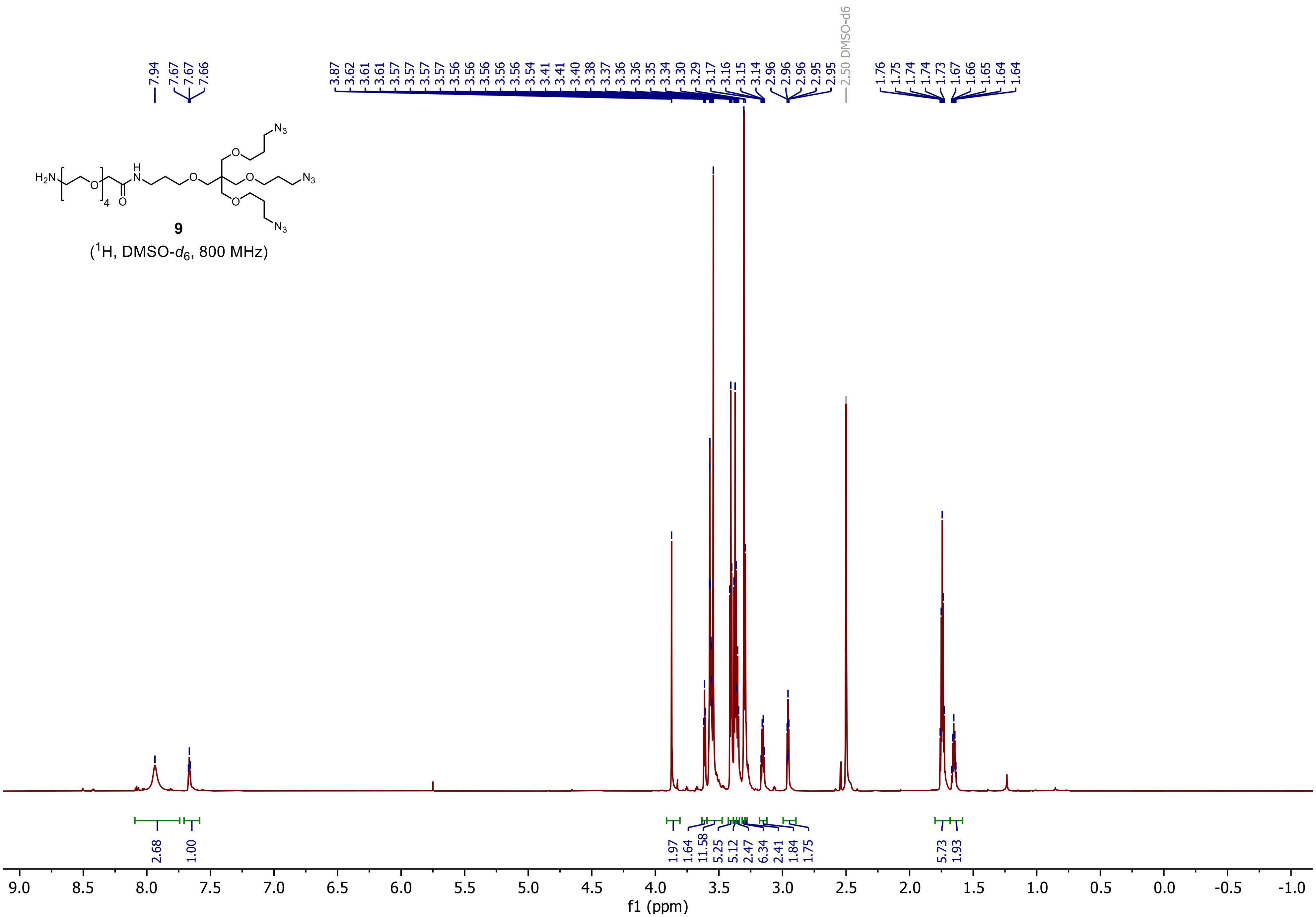
7.94
7.67
7.67
7.66
3.87
3.62
3.61
3.61
3.57
3.57
3.57
3.57
3.56
3.56
3.56
3.56
3.56
3.54
3.41
3.41
3.40
3.38
3.37
3.36
3.36
3.35
3.34
3.30
3.29
3.17
3.16
3.15
3.14
2.96
2.96
2.96
2.95
2.95
2.50 DMSO-d6
1.76
1.75
1.74
1.74
1.73
1.67
1.66
1.65
1.64
1.64
9
(1H, DMSO-d6, 800 MHz)
2.68
1.00
1.97
1.64
11.58
5.25
5.12
2.47
6.34
2.41
1.84
1.75
5.73
1.93
f1 (ppm)

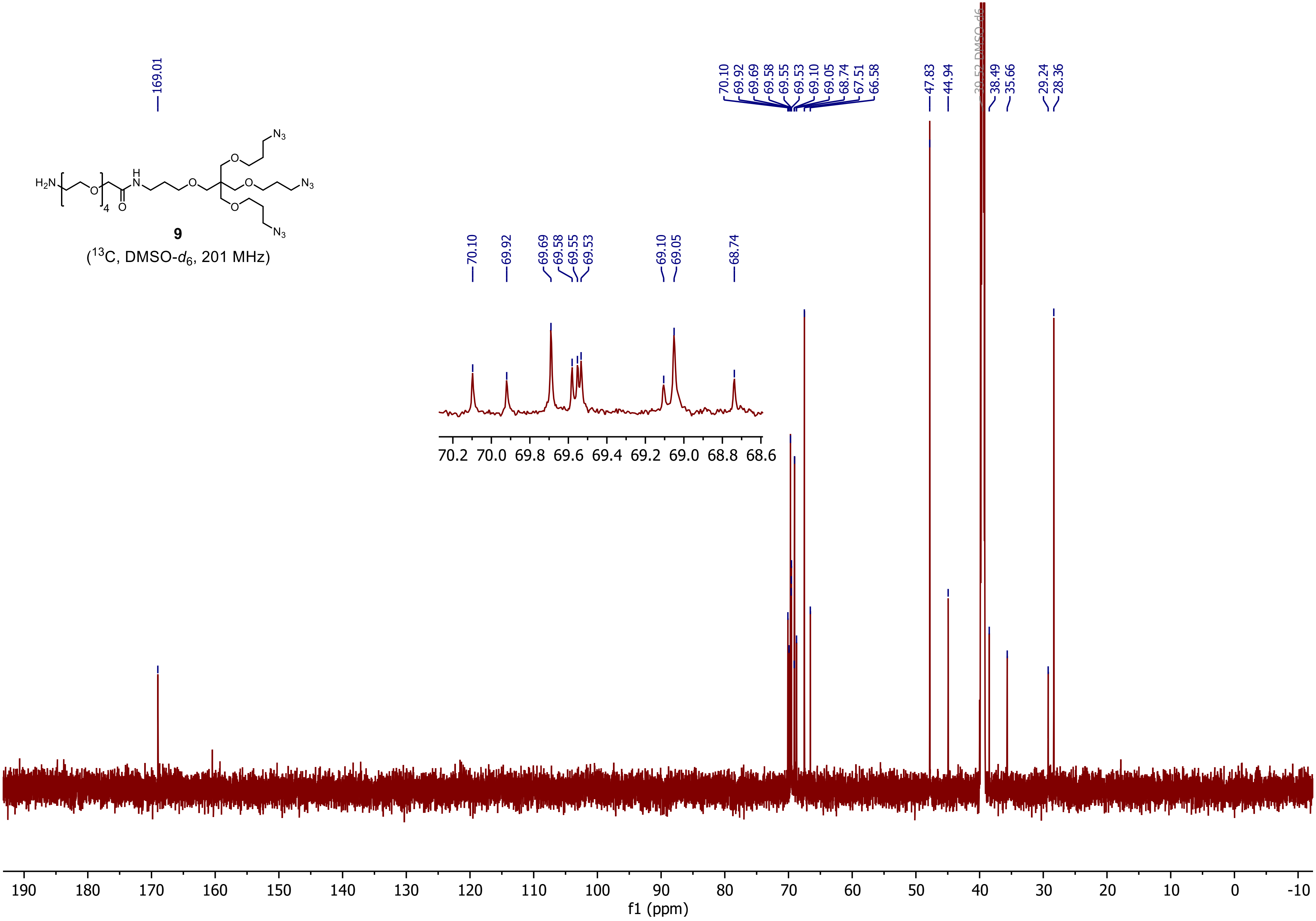
H2N
O
4
O
H
N
O
O
N3
O
N3
O
N3
9
(13C, DMSO-d6, 201 MHz)
169.01
70.10
69.92
69.69
69.58
69.55
69.53
69.10
69.05
68.74
67.51
66.58
47.83
44.94
39.52 DMSO-d6
38.49
35.66
29.24
28.36
70.2 70.0 69.8 69.6 69.4 69.2 69.0 68.8 68.6
190 180 170 160 150 140 130 120 110 100 90 80 70 60 50 40 30 20 10 0 -10
f1 (ppm)

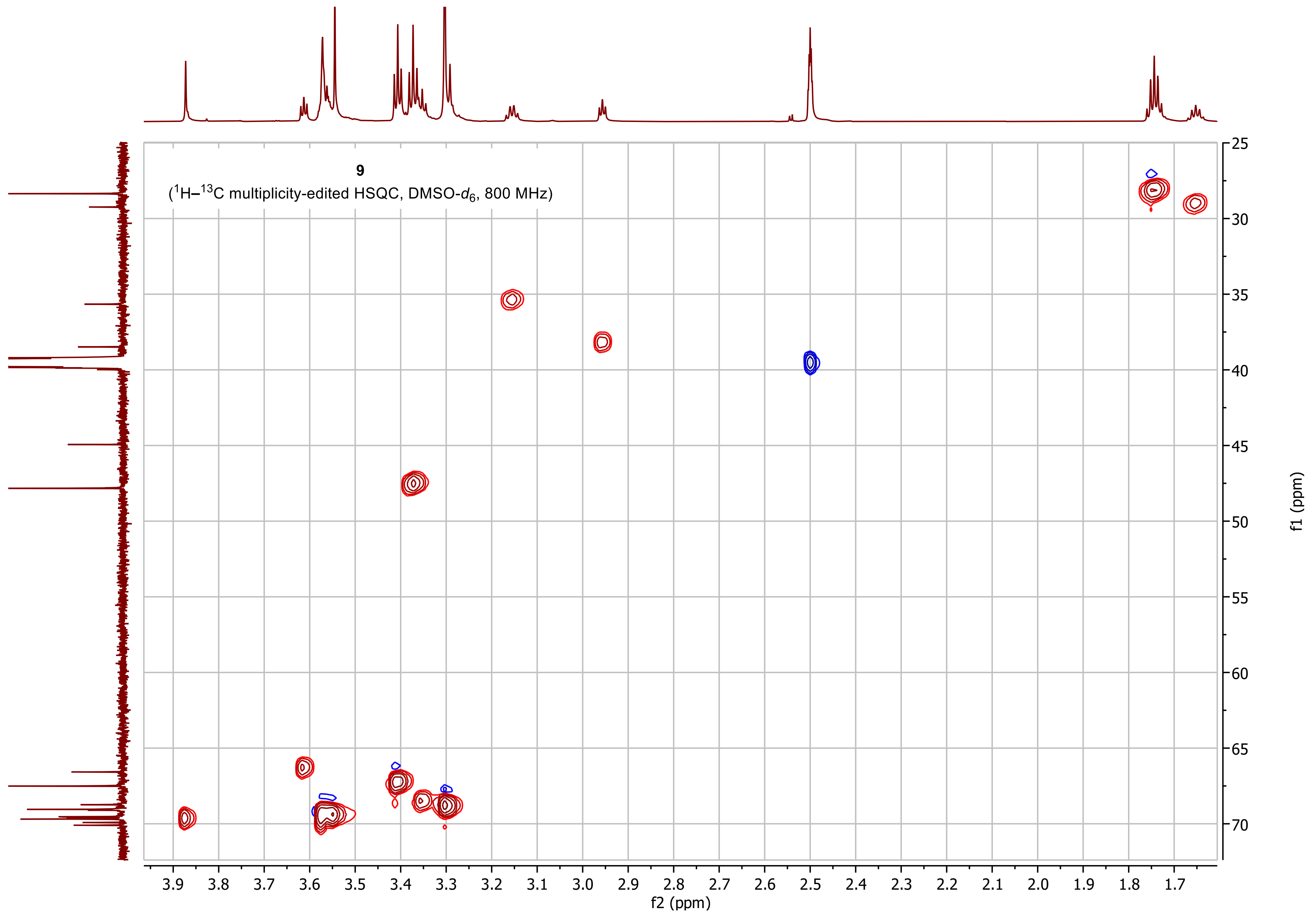
9
(^{1}H–^{13}C multiplicity-edited HSQC, DMSO-d_6, 800 MHz)
3.9 3.8 3.7 3.6 3.5 3.4 3.3 3.2 3.1 3.0 2.9 2.8 2.7 2.6 2.5 2.4 2.3 2.2 2.1 2.0 1.9 1.8 1.7
f2 (ppm)
25 30 35 40 45 50 55 60 65 70
f1 (ppm)

9

(^{1}H–^{13}C HMBC, DMSO-d_6, 800 MHz)

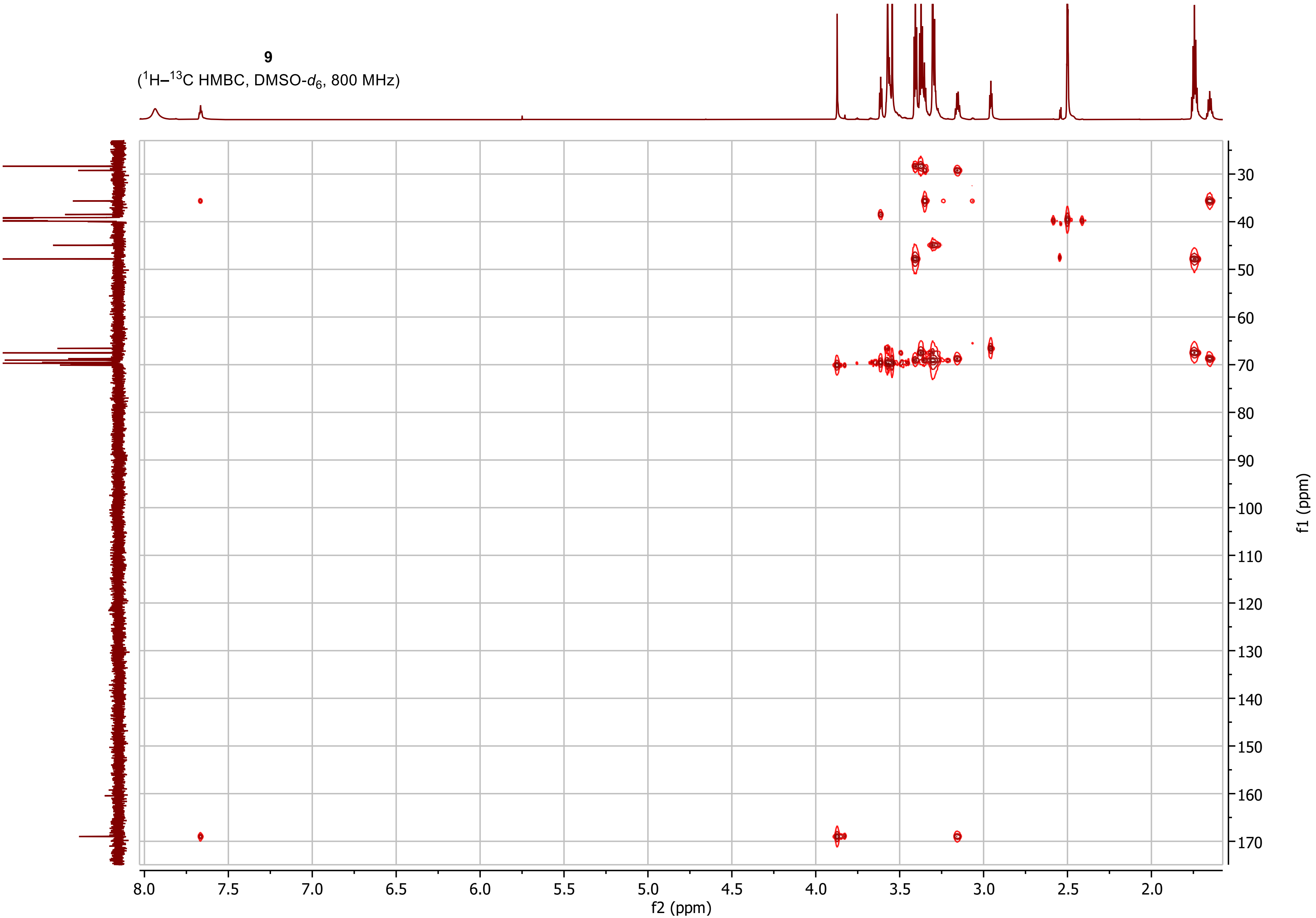

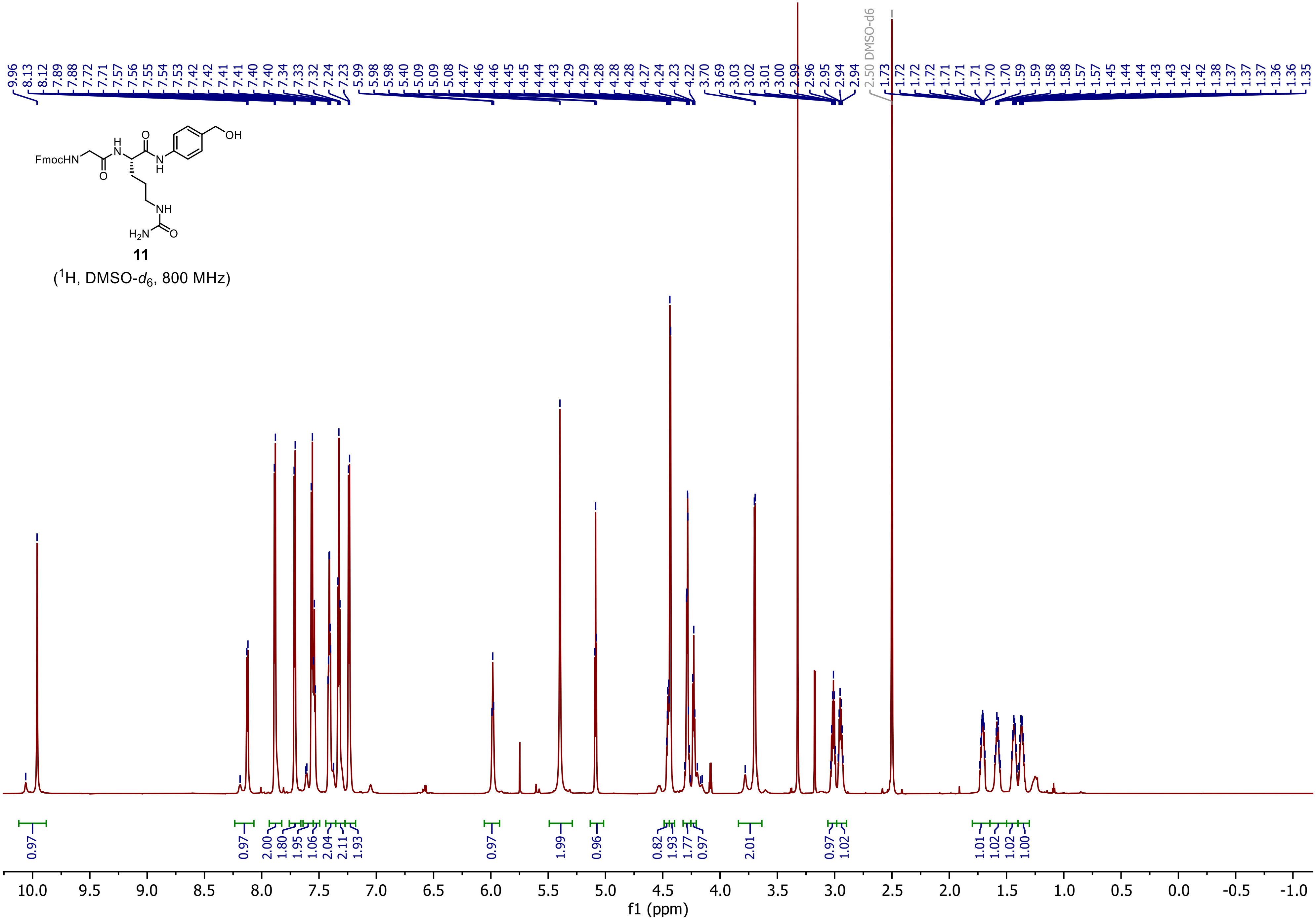
FmocHN
OH
11
(¹H, DMSO-d6, 800 MHz)
f1 (ppm)
2.50 DMSO-d6

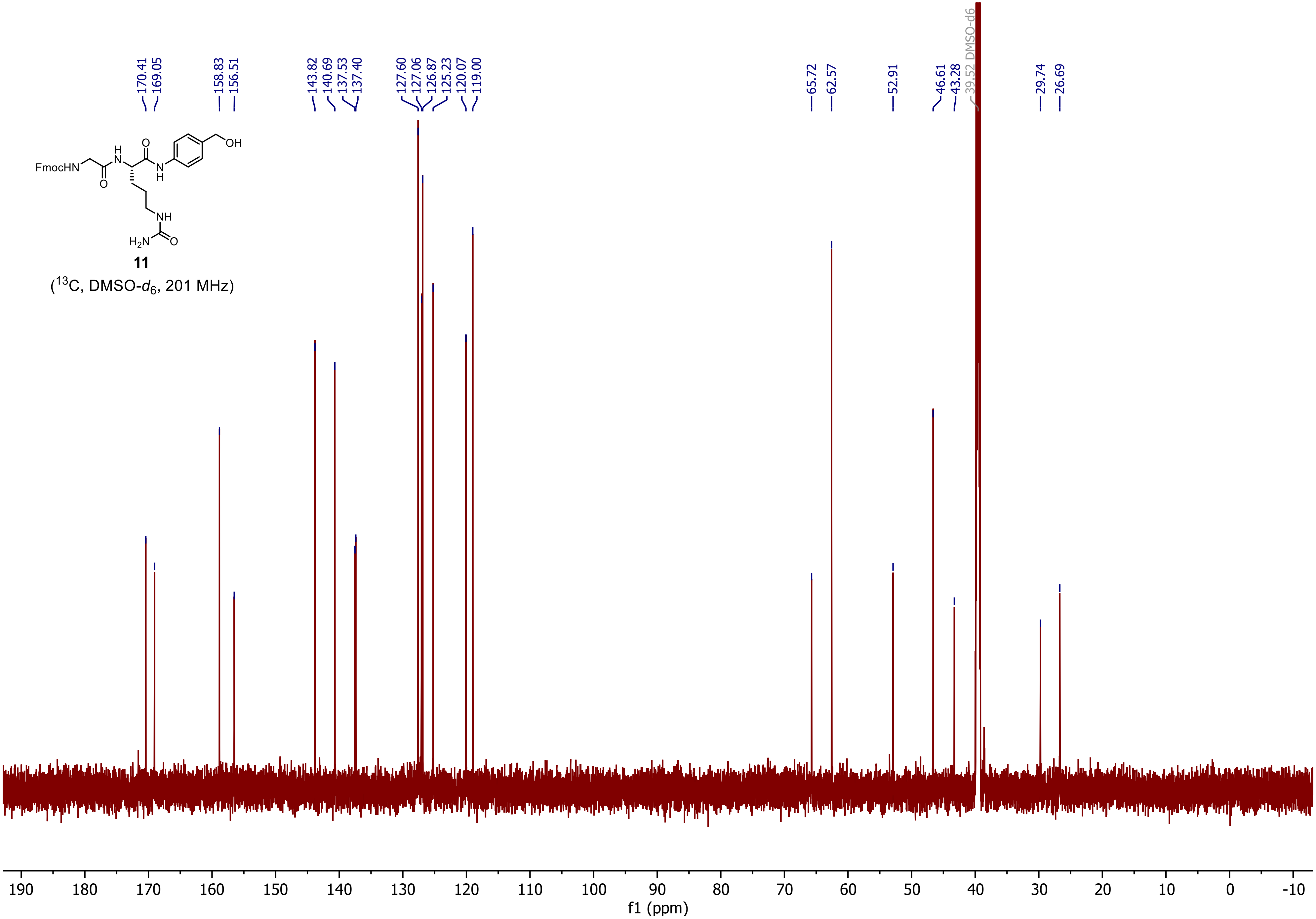
170.41
169.05
158.83
156.51
143.82
140.69
137.53
137.40
127.60
127.06
126.87
125.23
120.07
119.00
65.72
62.57
52.91
46.61
43.28
39.52 DMSO-d6
29.74
26.69
FmocHN
H
N
O
N
H
OH
NH
H2N
O
11
(13C, DMSO-d6, 201 MHz)
190
180
170
160
150
140
130
120
110
100
90
80
70
60
50
40
30
20
10
0
-10
f1 (ppm)

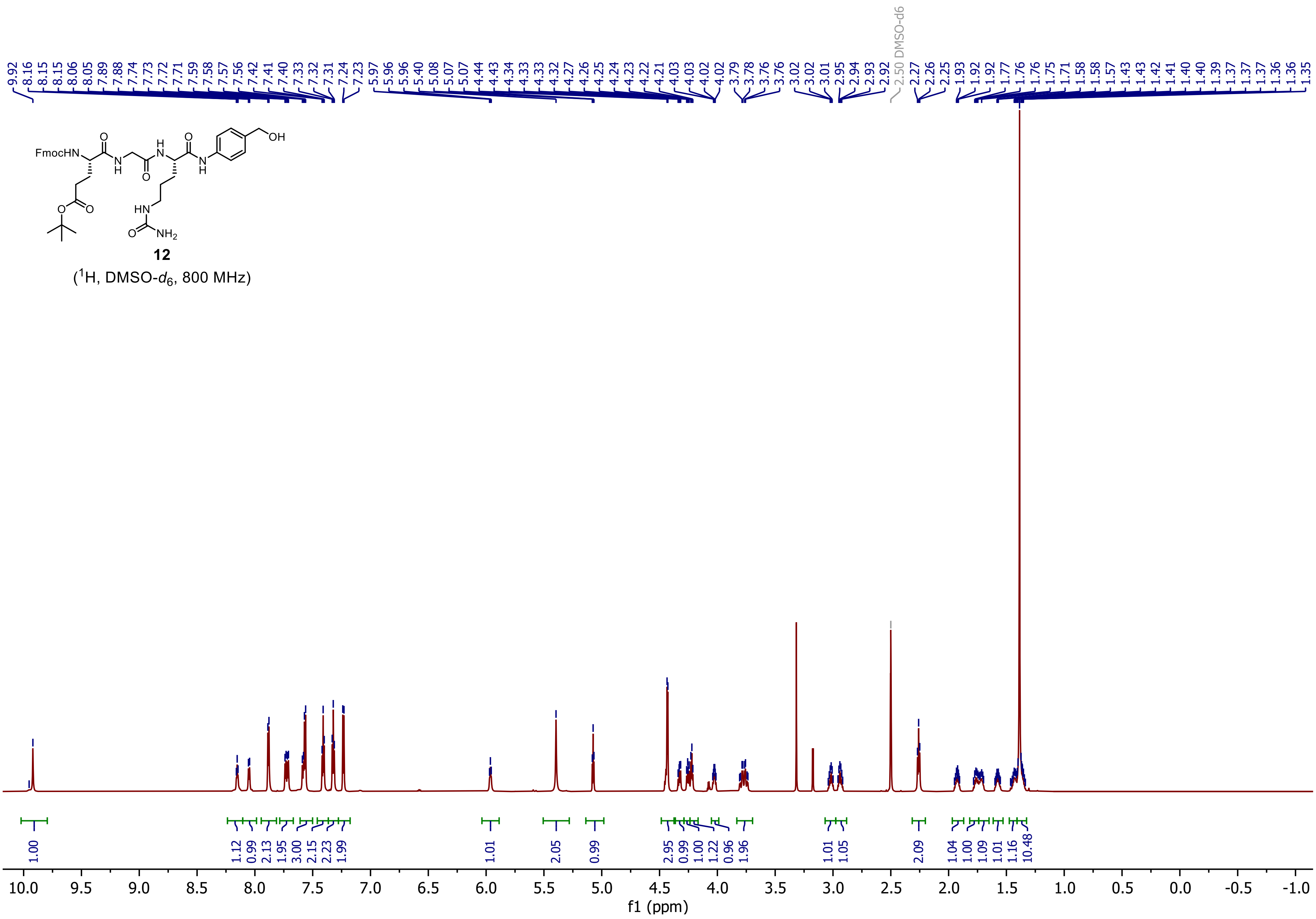
FmocHN
OH
12
(1H, DMSO-d6, 800 MHz)
DMSO-d6
f1 (ppm)

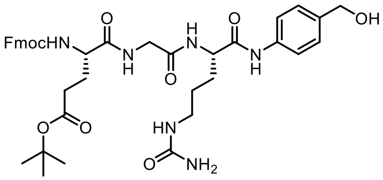

12

(^{13}C, DMSO-d_6, 201 MHz)

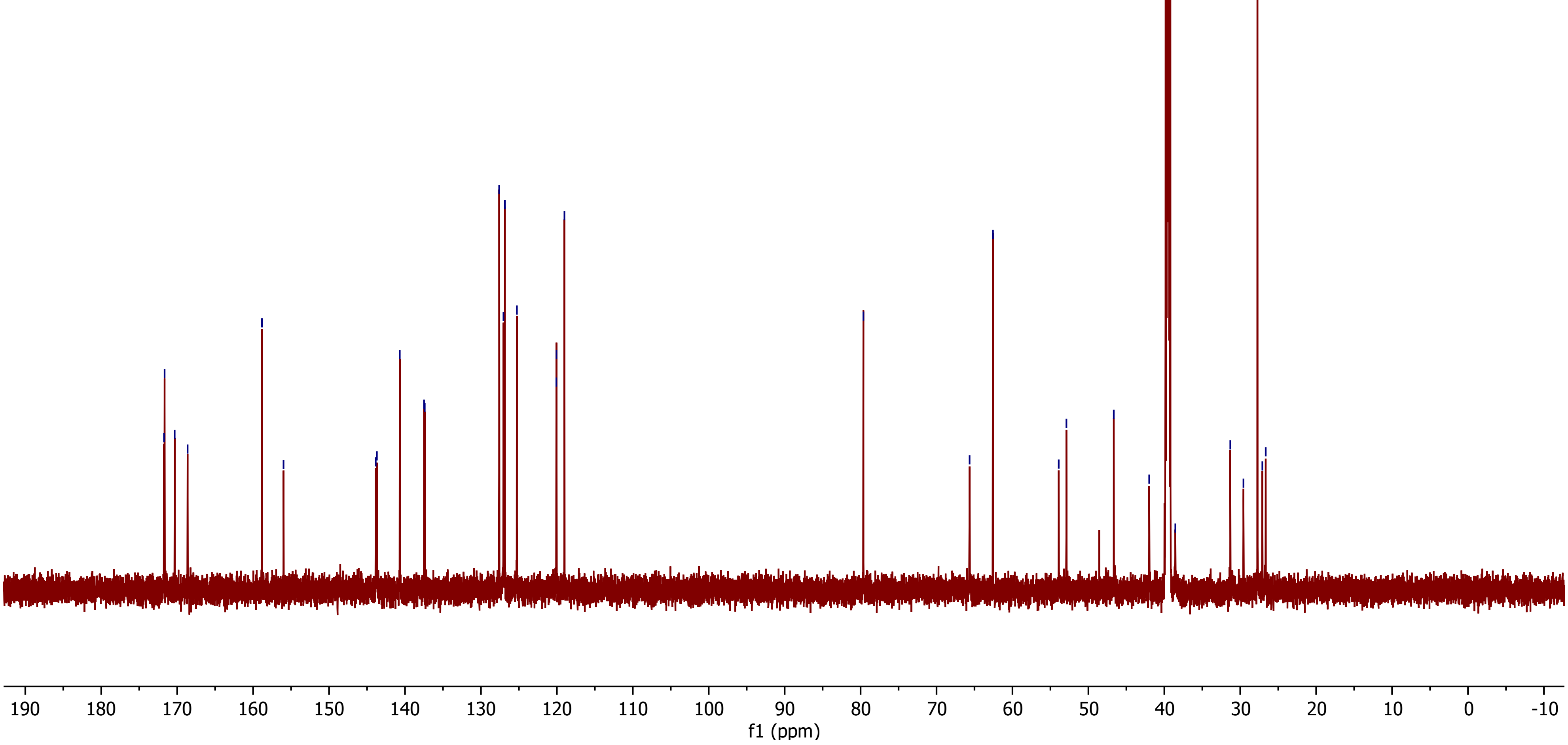

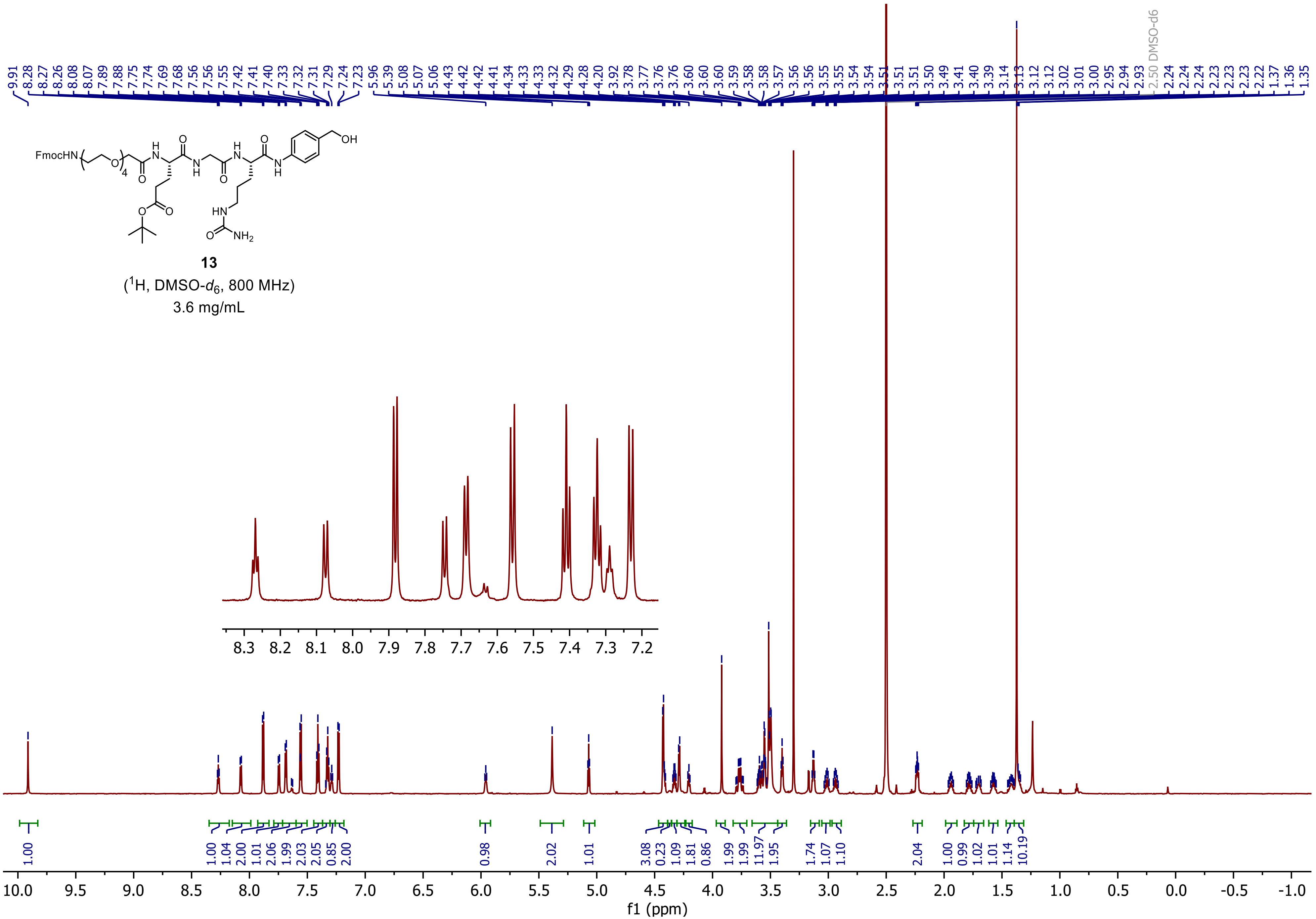
9.91 8.28 8.27 8.26 8.08 8.07 7.89 7.88 7.75 7.74 7.69 7.68 7.56 7.55 7.42 7.41 7.40 7.33 7.32 7.31 7.29 7.24 7.23 5.96 5.39 5.08 5.07 5.06 4.43 4.42 4.42 4.41 4.34 4.33 4.33 4.32 4.29 4.28 4.20 3.92 3.78 3.77 3.76 3.76 3.60 3.60 3.59 3.58 3.58 3.57 3.56 3.56 3.55 3.55 3.54 3.51 3.51 3.51 3.50 3.49 3.41 3.40 3.39 3.14 3.13 3.12 3.12 3.02 3.01 3.00 2.95 2.94 2.93 2.50 DMSO-d6 2.24 2.24 2.24 2.23 2.23 2.23 2.22 1.37 1.36 1.35
FmocHN
O
4
H
N
O
O
OH
NH
HN
NH2
13
(1H, DMSO-d6, 800 MHz)
3.6 mg/mL
8.3 8.2 8.1 8.0 7.9 7.8 7.7 7.6 7.5 7.4 7.3 7.2
1.00 1.00 1.04 2.00 1.01 2.06 1.99 2.03 2.05 0.85 2.00 0.98 2.02 1.01 3.08 0.23 1.09 1.81 0.86 1.99 1.99 11.97 1.95 1.74 1.07 1.10 2.04 1.00 0.99 1.02 1.01 1.14 10.19
10.0 9.5 9.0 8.5 8.0 7.5 7.0 6.5 6.0 5.5 5.0 4.5 4.0 3.5 3.0 2.5 2.0 1.5 1.0 0.5 0.0 -0.5 -1.0
f1 (ppm)

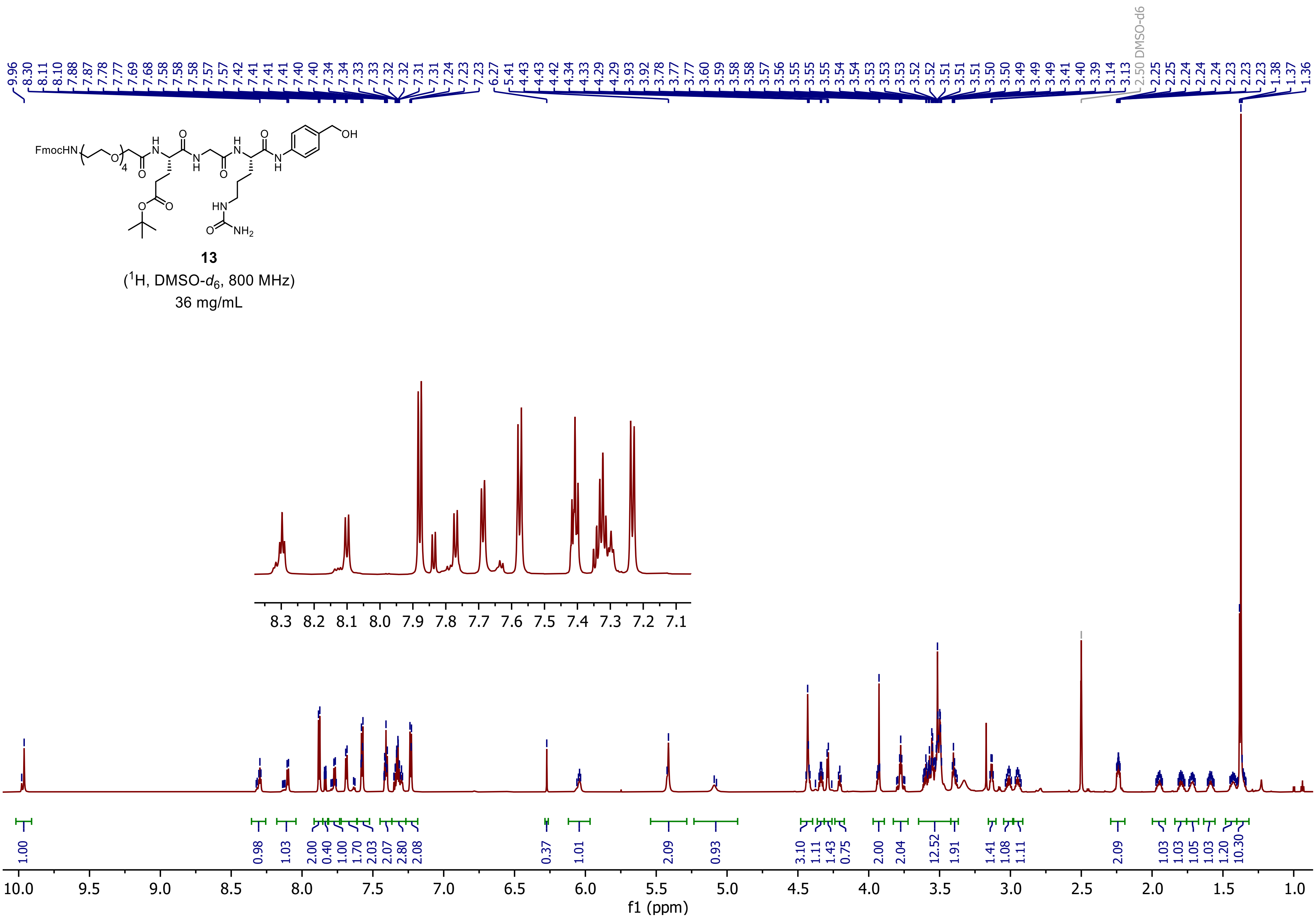

FmocHN
OH
NH2
13
(1H, DMSO-d6, 800 MHz)
36 mg/mL
2.50 DMSO-d6
f1 (ppm)

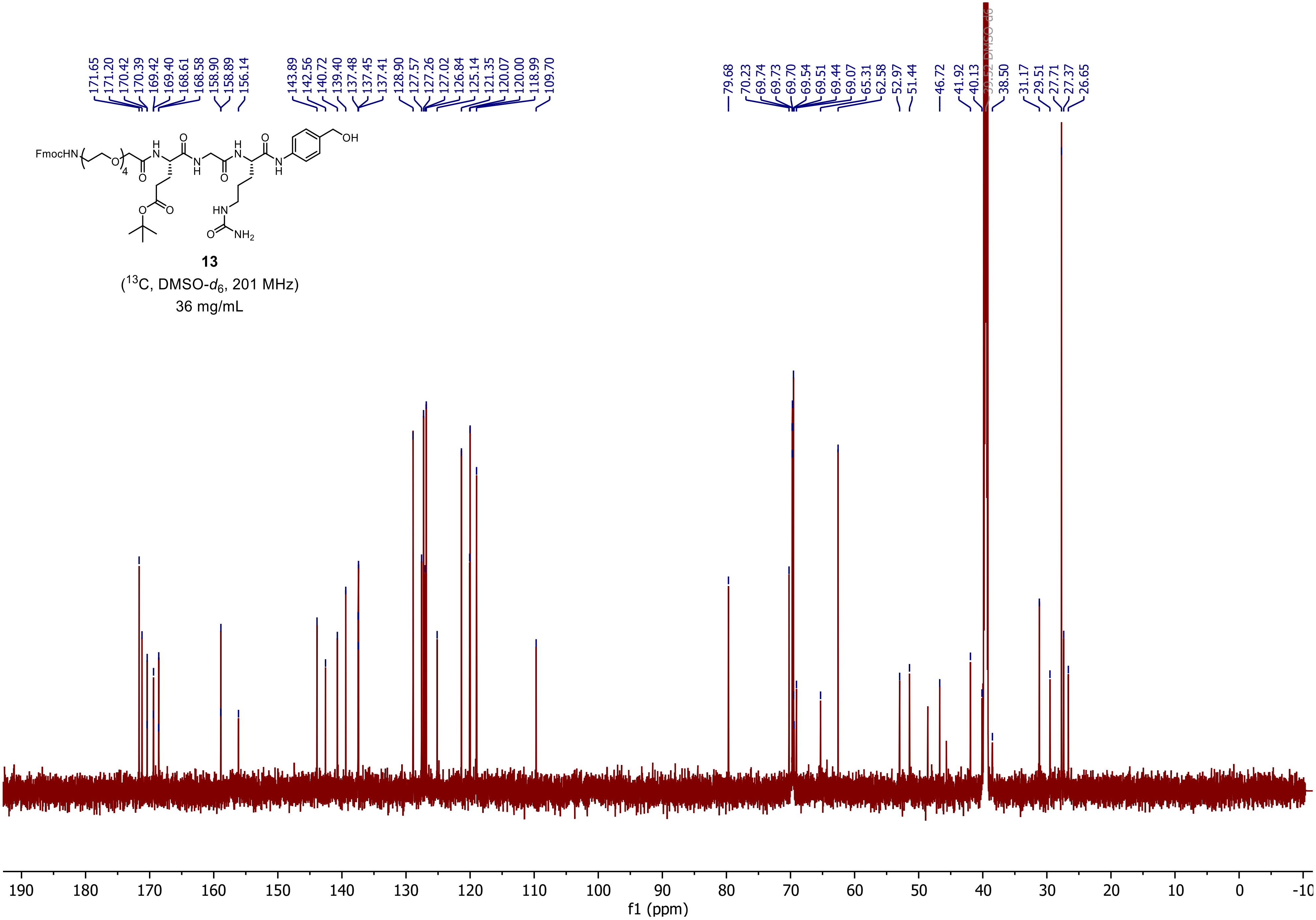

13

(^{13}C, DMSO-d_6, 201 MHz)

36 mg/mL

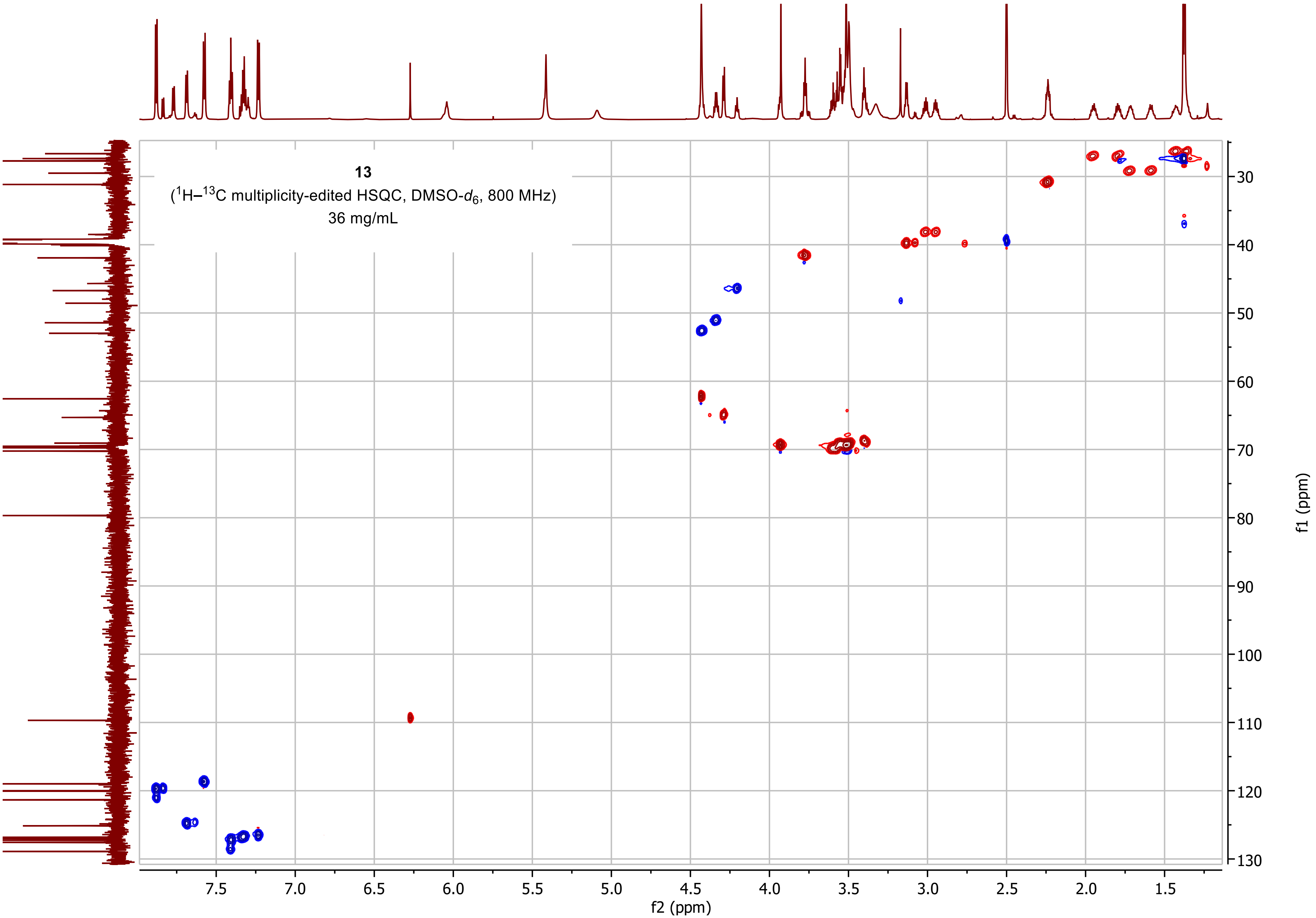

13
(1H–13C multiplicity-edited HSQC, DMSO-d6, 800 MHz)
36 mg/mL
f2 (ppm)
f1 (ppm)

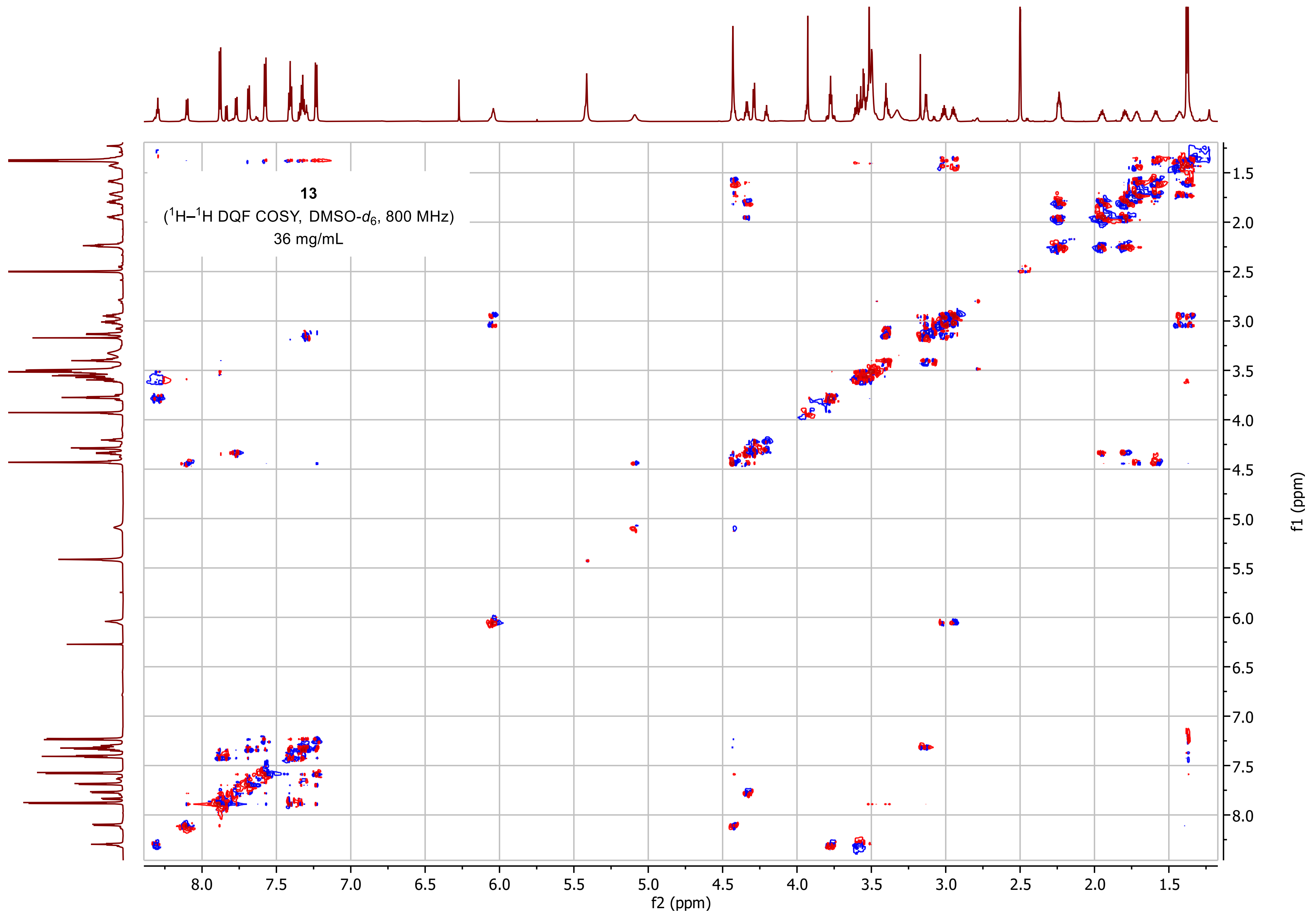

13
(^{1}H–^{1}H DQF COSY, DMSO-d_6, 800 MHz)
36 mg/mL
f2 (ppm)
f1 (ppm)

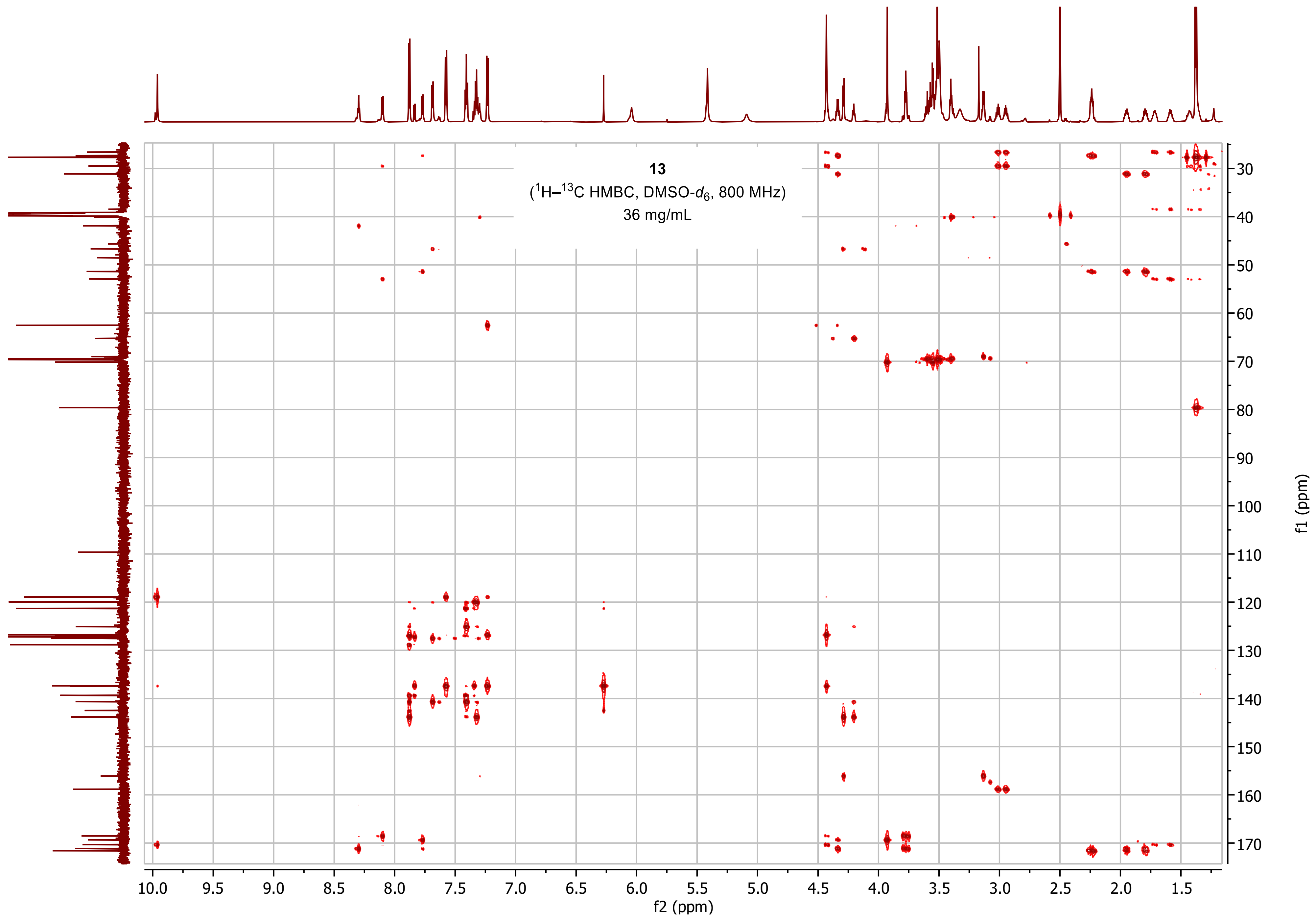

13
(^{1}H–^{13}C HMBC, DMSO-d_6, 800 MHz)
36 mg/mL
f2 (ppm)
f1 (ppm)

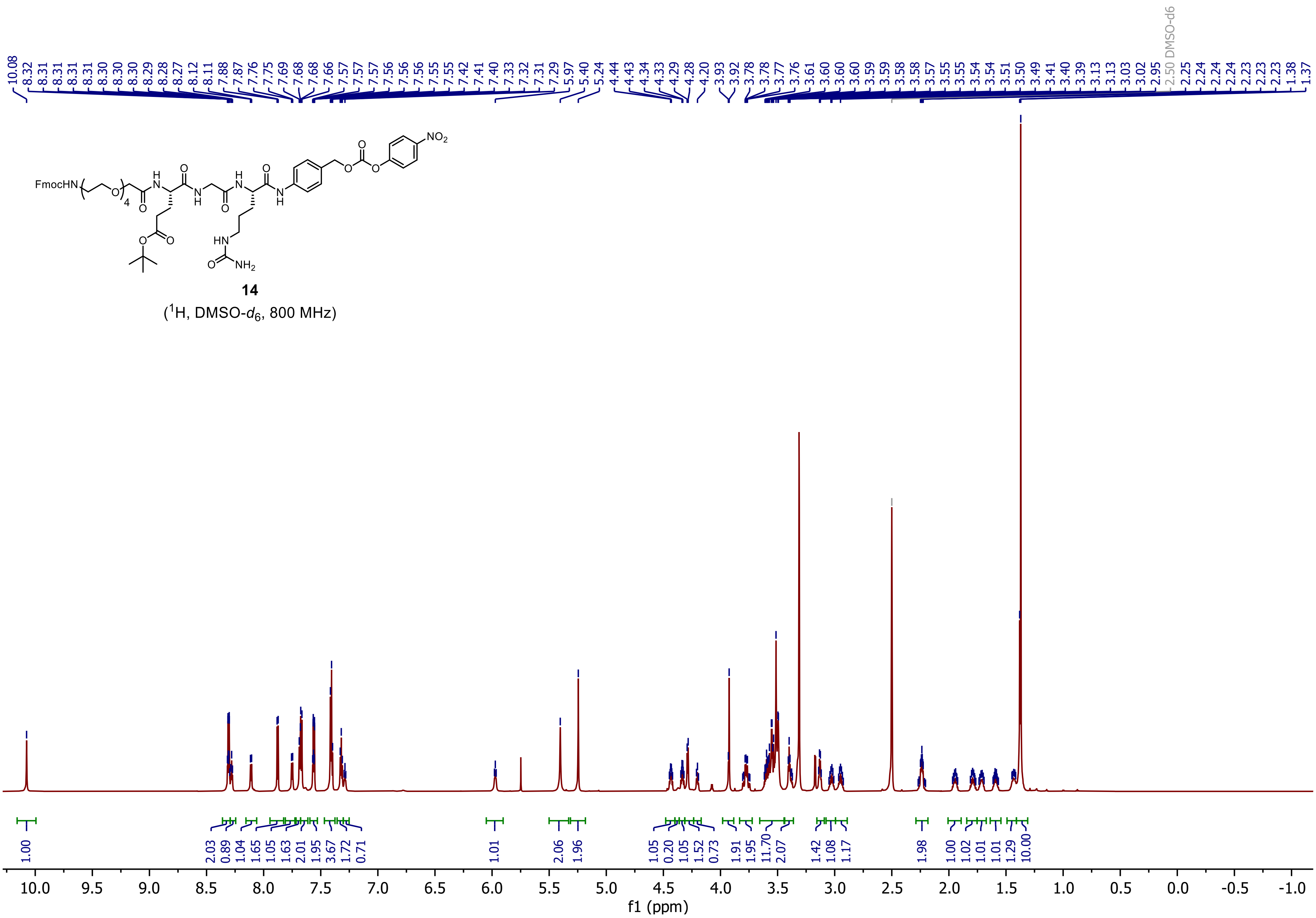
FmocHN
NO2
14
(1H, DMSO-d6, 800 MHz)
2.50 DMSO-d6
f1 (ppm)

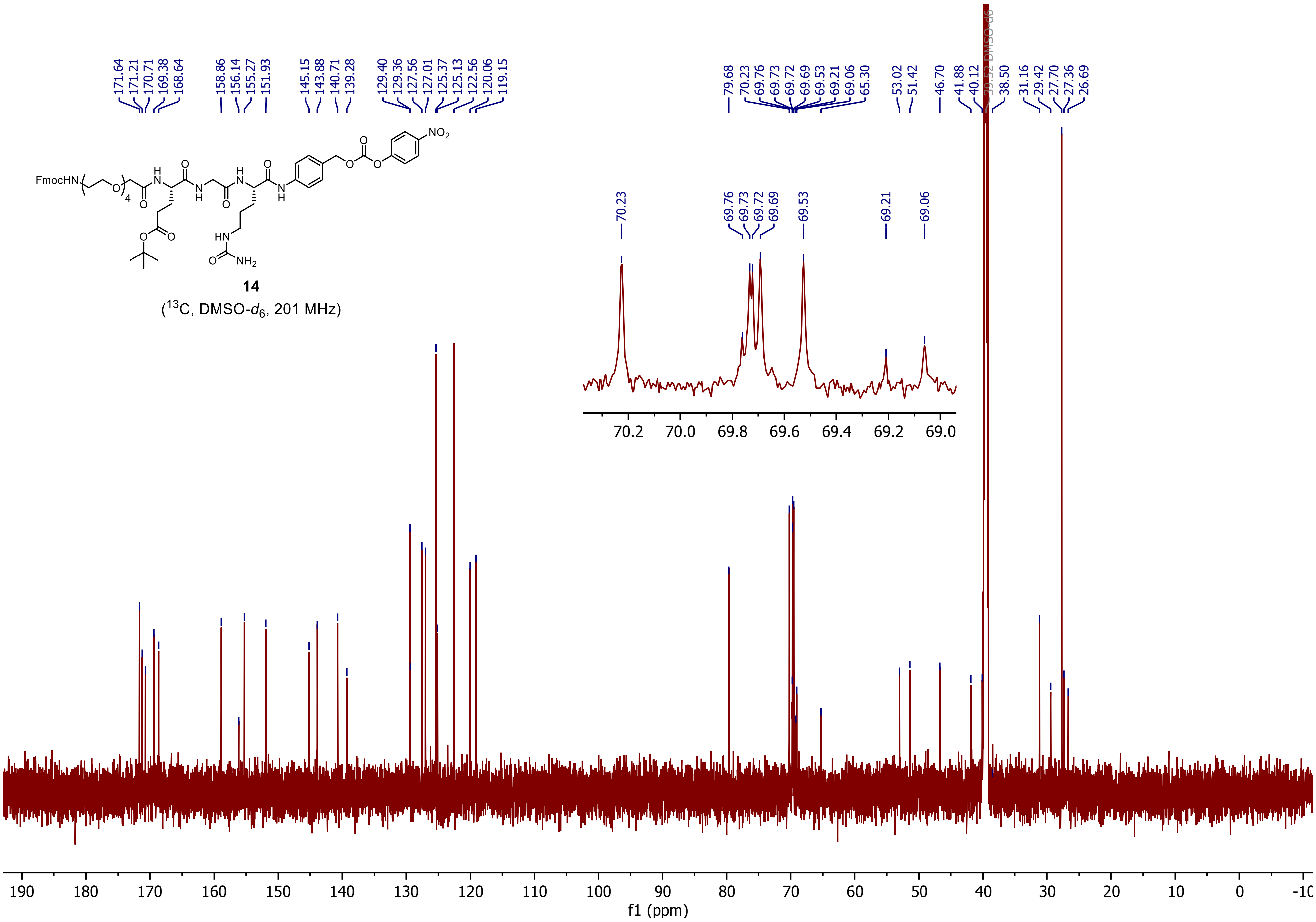
171.64
171.21
170.71
169.38
168.64
158.86
156.14
155.27
151.93
145.15
143.88
140.71
139.28
129.40
129.36
127.56
127.01
125.37
125.13
122.56
120.06
119.15
79.68
70.23
69.76
69.73
69.72
69.69
69.53
69.21
69.06
65.30
53.02
51.42
46.70
41.88
40.12
39.52 DMSO-d6
38.50
31.16
29.42
27.70
27.36
26.69
FmocHN
O
4
H
N
O
O
N
H
N
H
O
N
H
O
NO2
O
O
O
HN
O
NH2
14
(13C, DMSO-d6, 201 MHz)
70.2
70.0
69.8
69.6
69.4
69.2
69.0
190
180
170
160
150
140
130
120
110
100
90
80
70
60
50
40
30
20
10
0
-10
f1 (ppm)

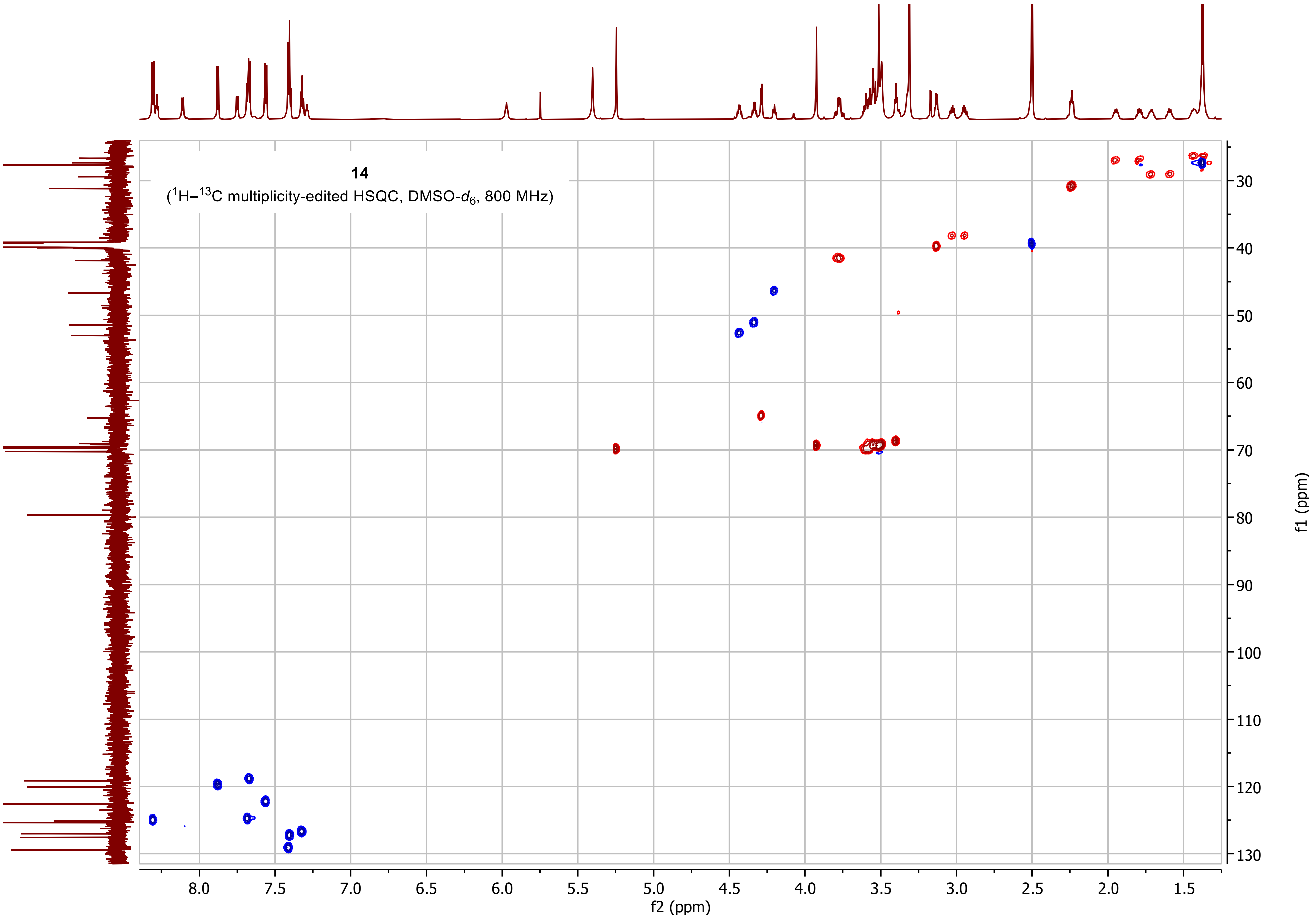

14
(^{1}H–^{13}C multiplicity-edited HSQC, DMSO-d_6, 800 MHz)
f2 (ppm)
f1 (ppm)

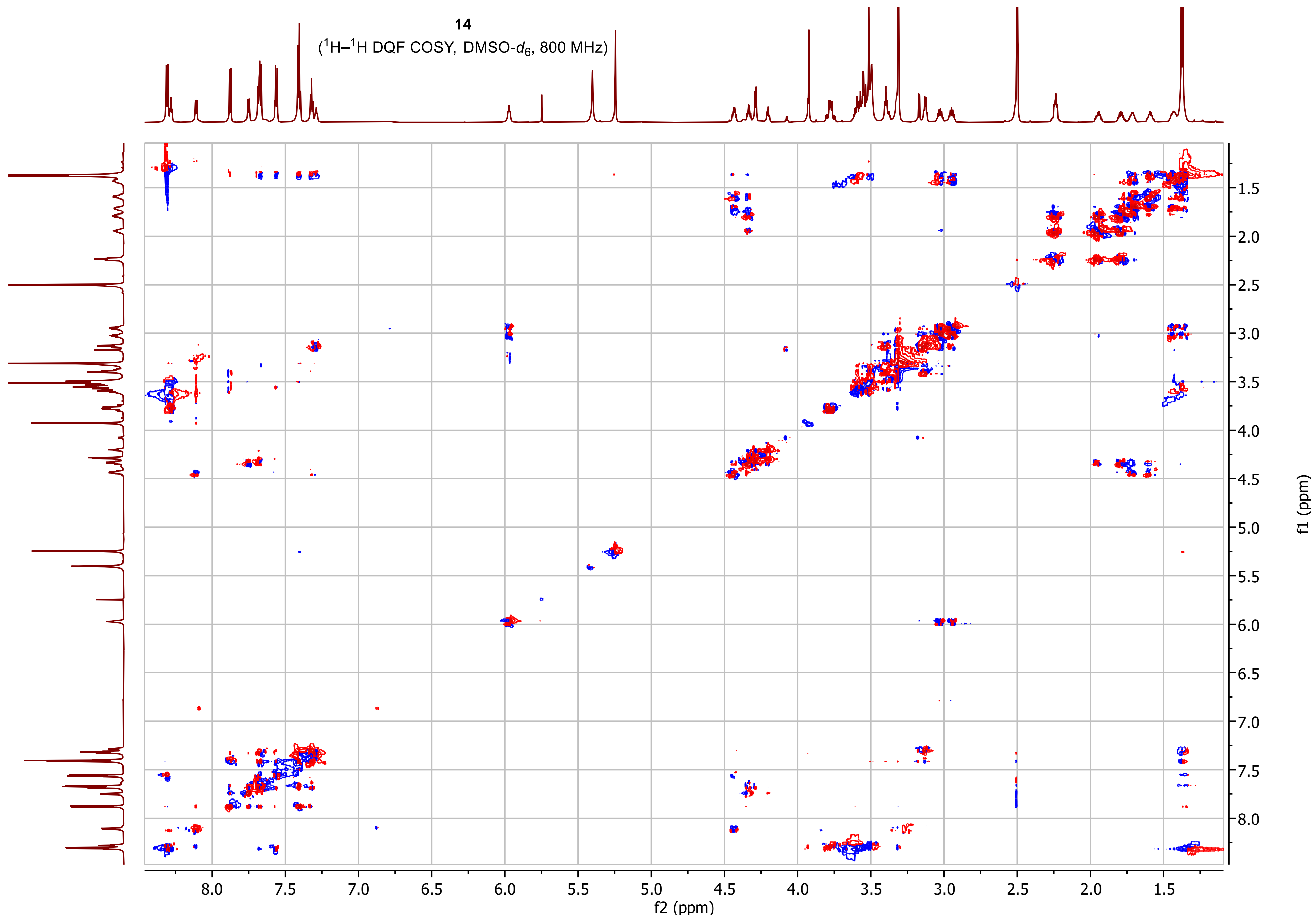
14
(1H–1H DQF COSY, DMSO-d6, 800 MHz)
f2 (ppm)
f1 (ppm)

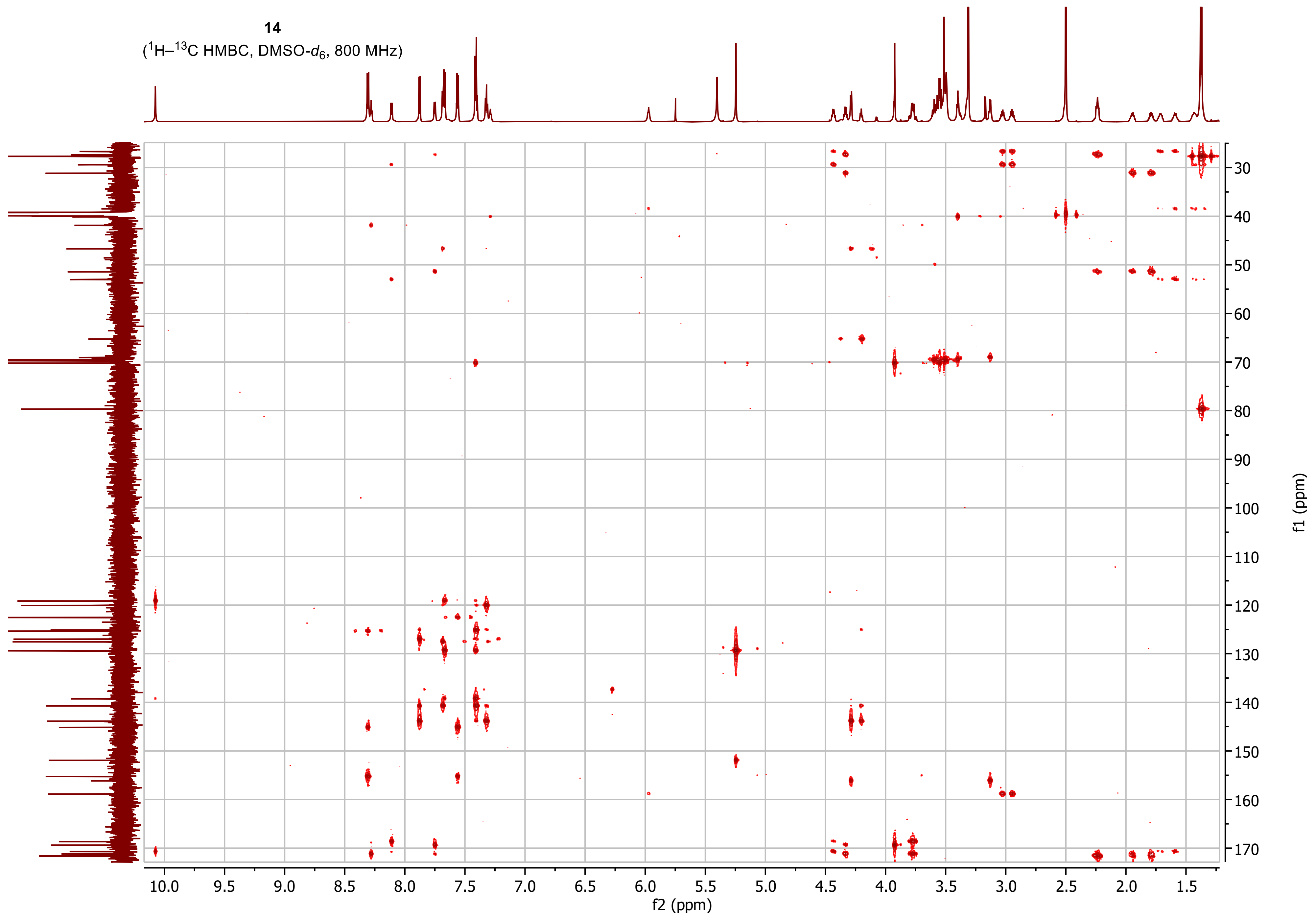
14
(1H–13C HMBC, DMSO-d6, 800 MHz)
f2 (ppm)
f1 (ppm)

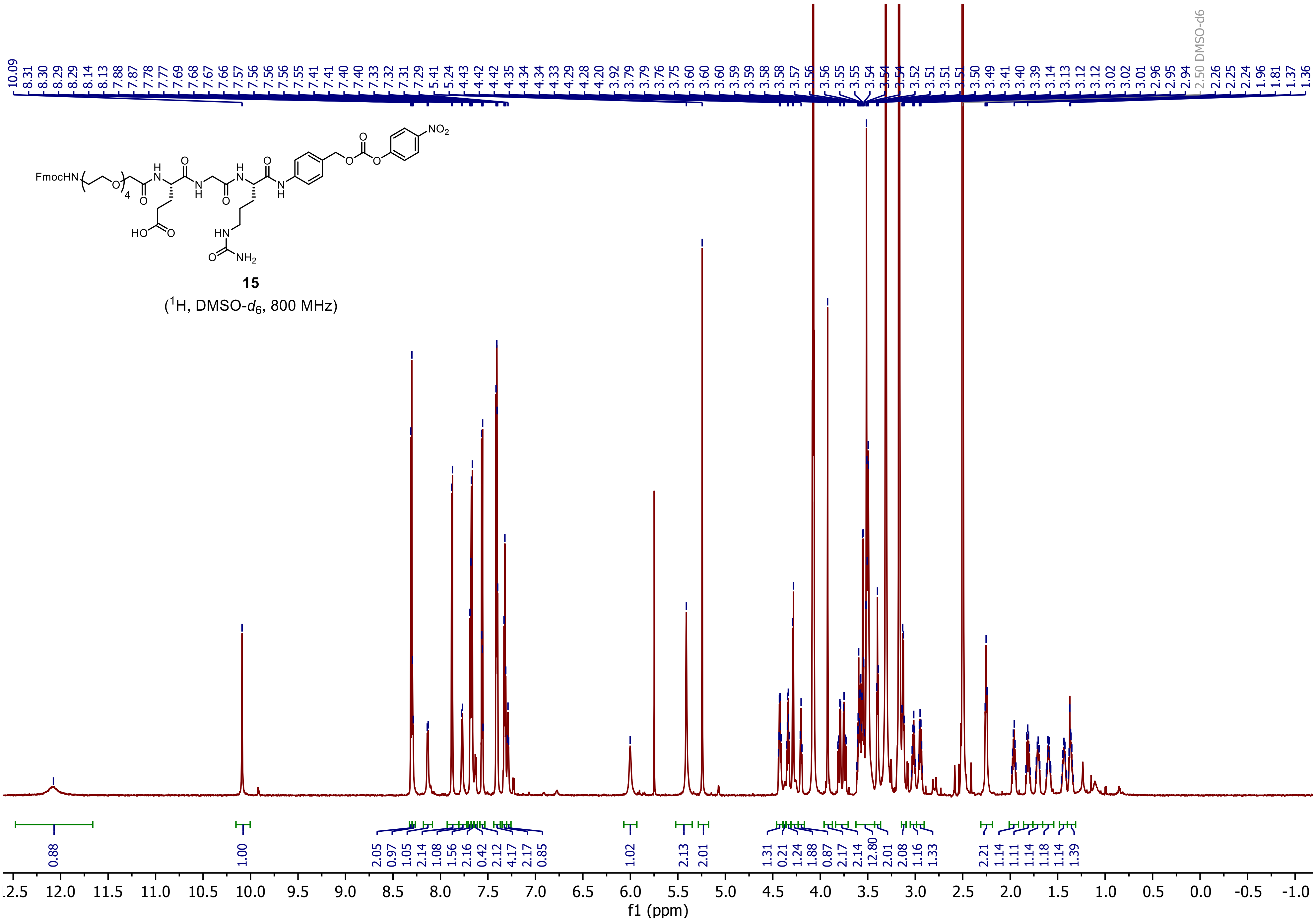

FmocHN
NO2
HO
HN
NH2
15
(1H, DMSO-d6, 800 MHz)
DMSO-d6
f1 (ppm)

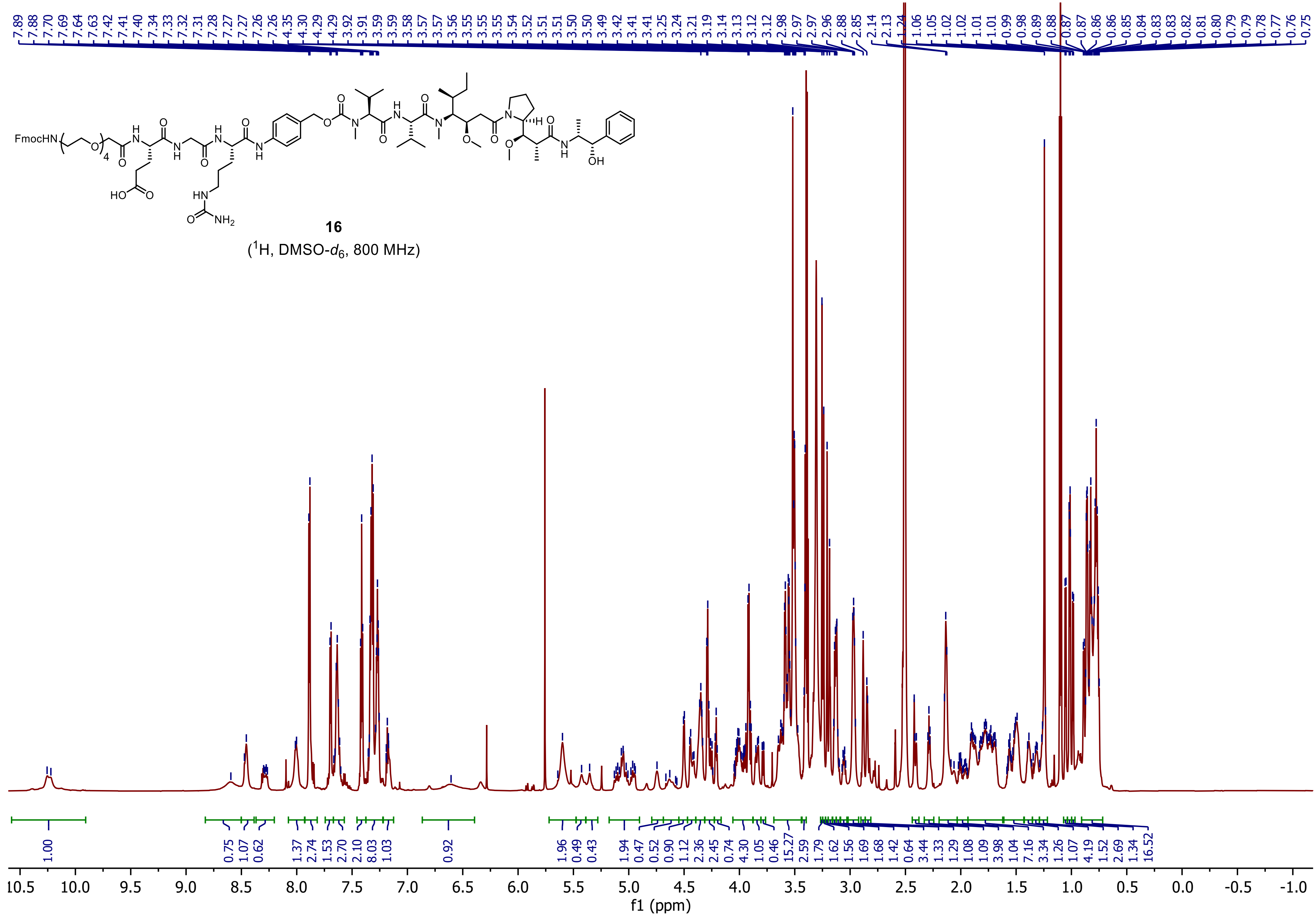

16
(^{1}H, DMSO-d_6, 800 MHz)

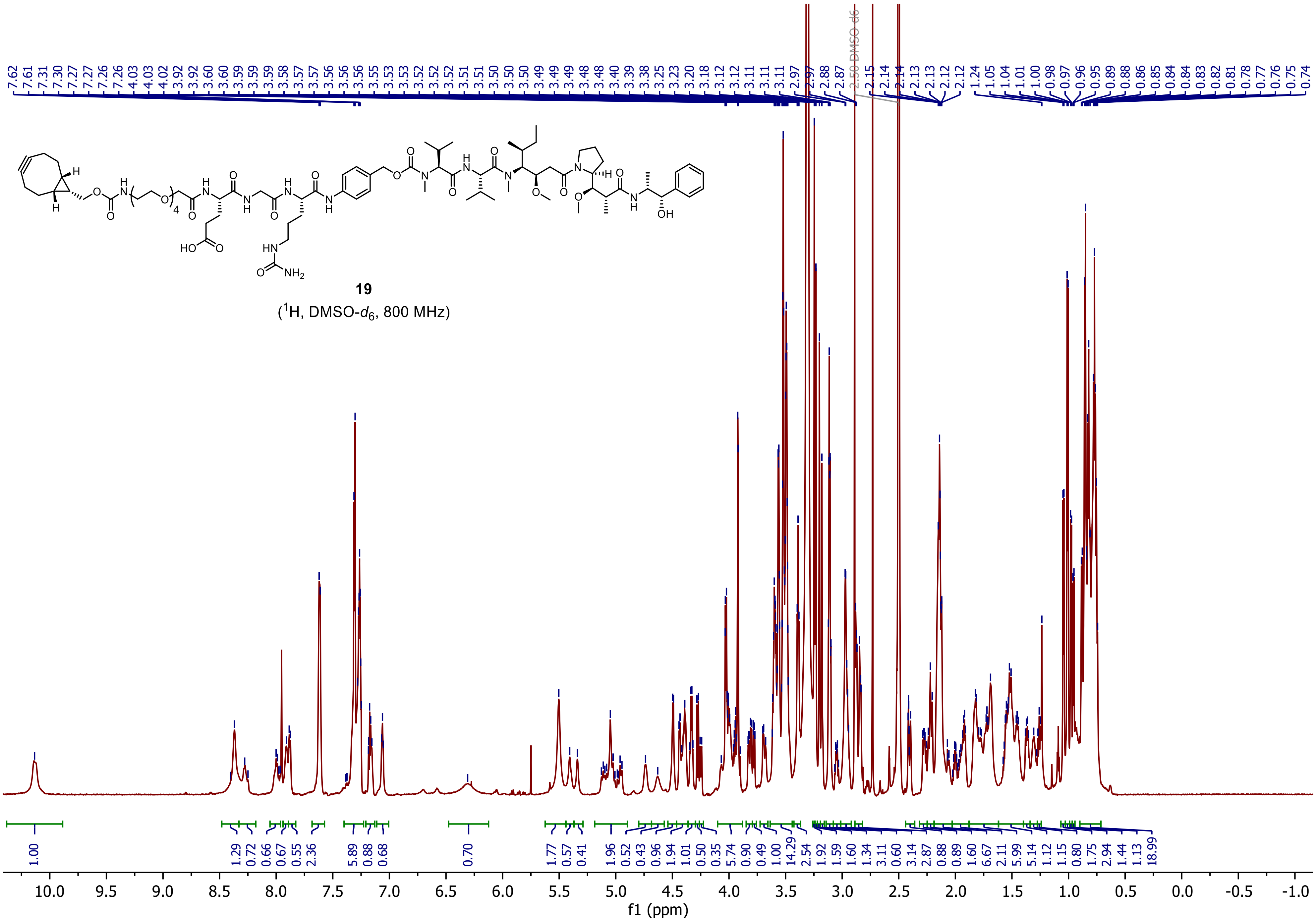

19

(^{1}H, DMSO-d_6, 800 MHz)

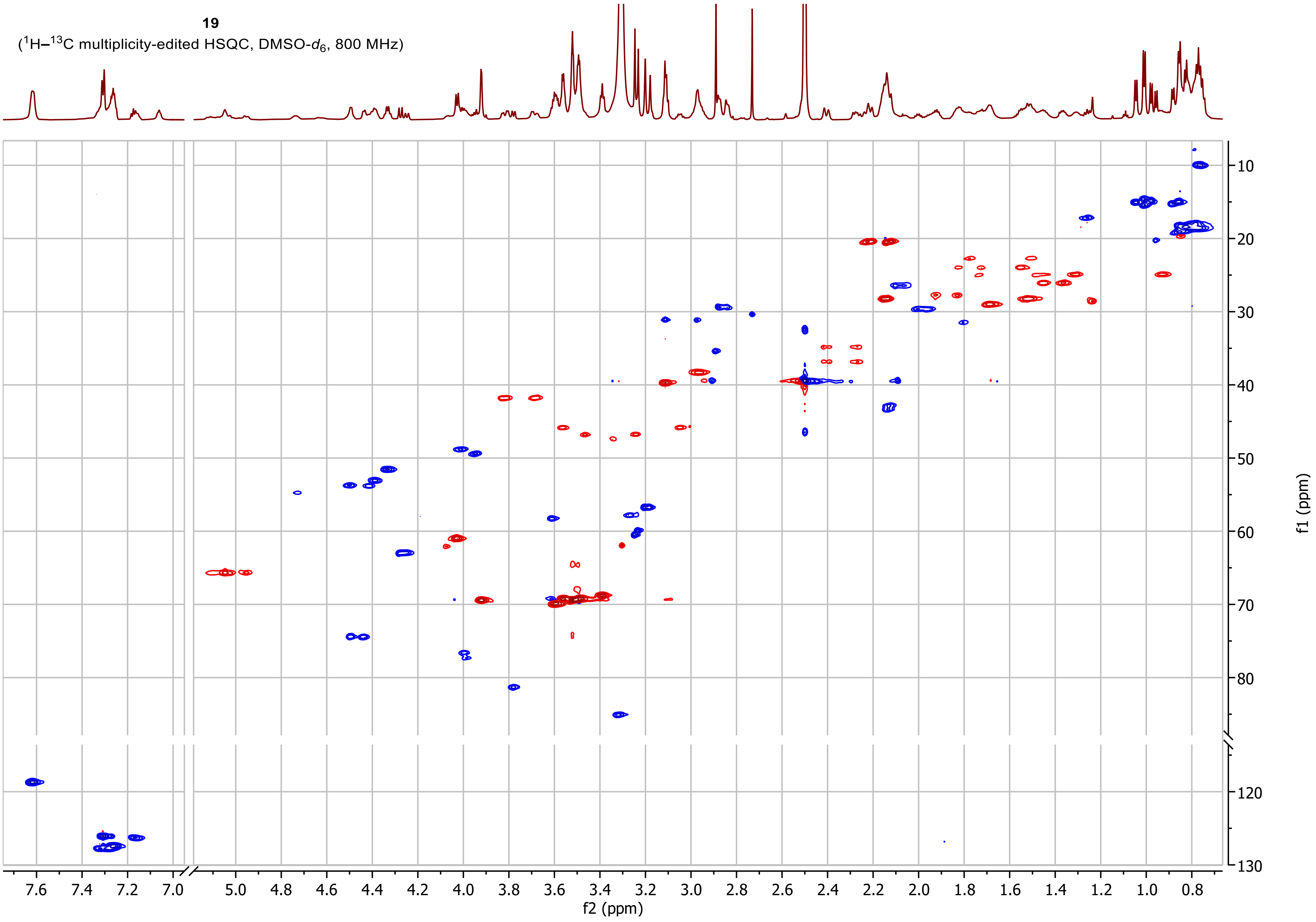

19
(¹H–¹³C multiplicity-edited HSQC, DMSO-d6, 800 MHz)
f2 (ppm)
f1 (ppm)

19

(^{1}H–^{1}H DQF COSY, DMSO-d_6, 800 MHz)

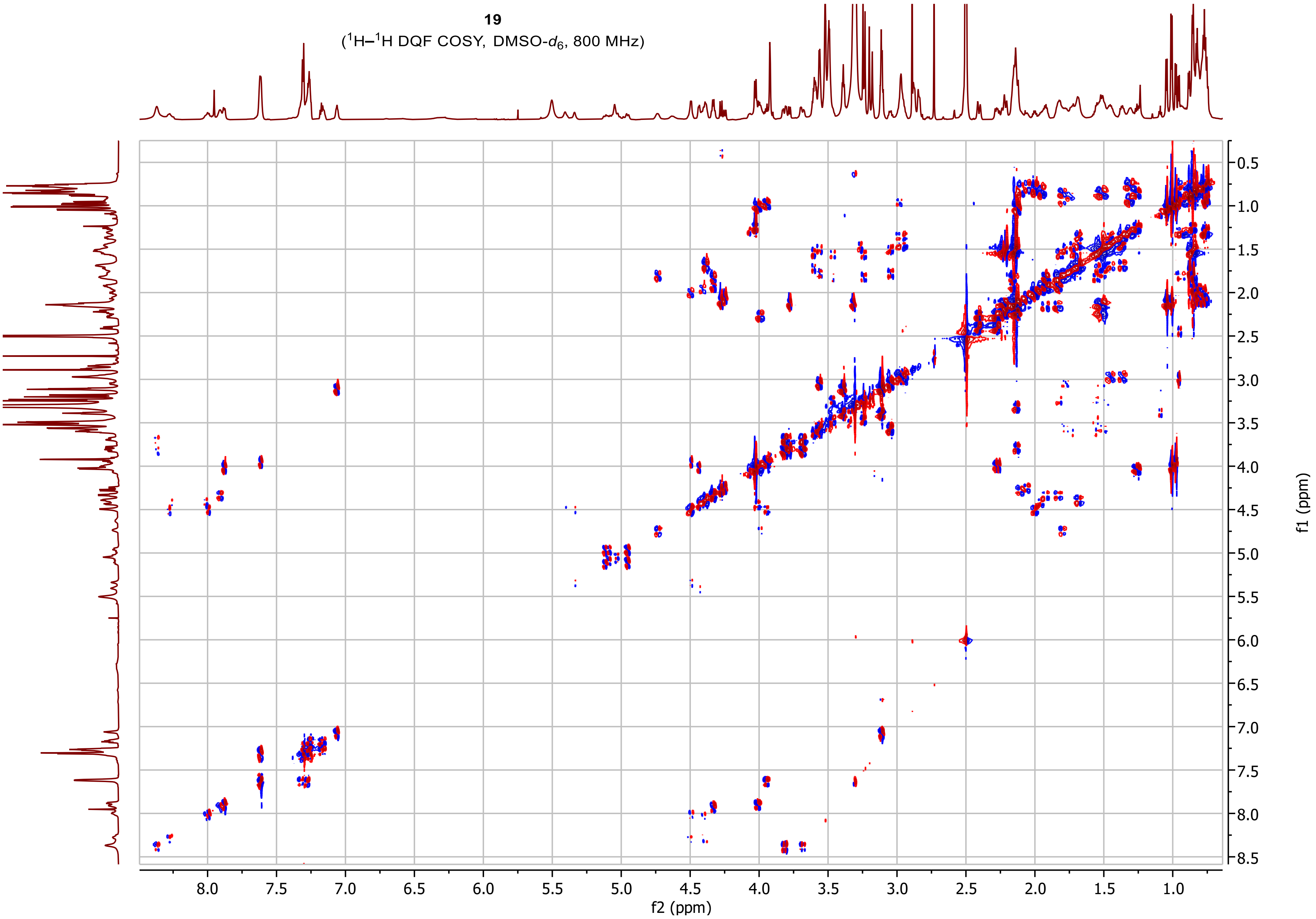

19
(^{1}H–^{13}C HMBC, DMSO-d_6, 800 MHz)

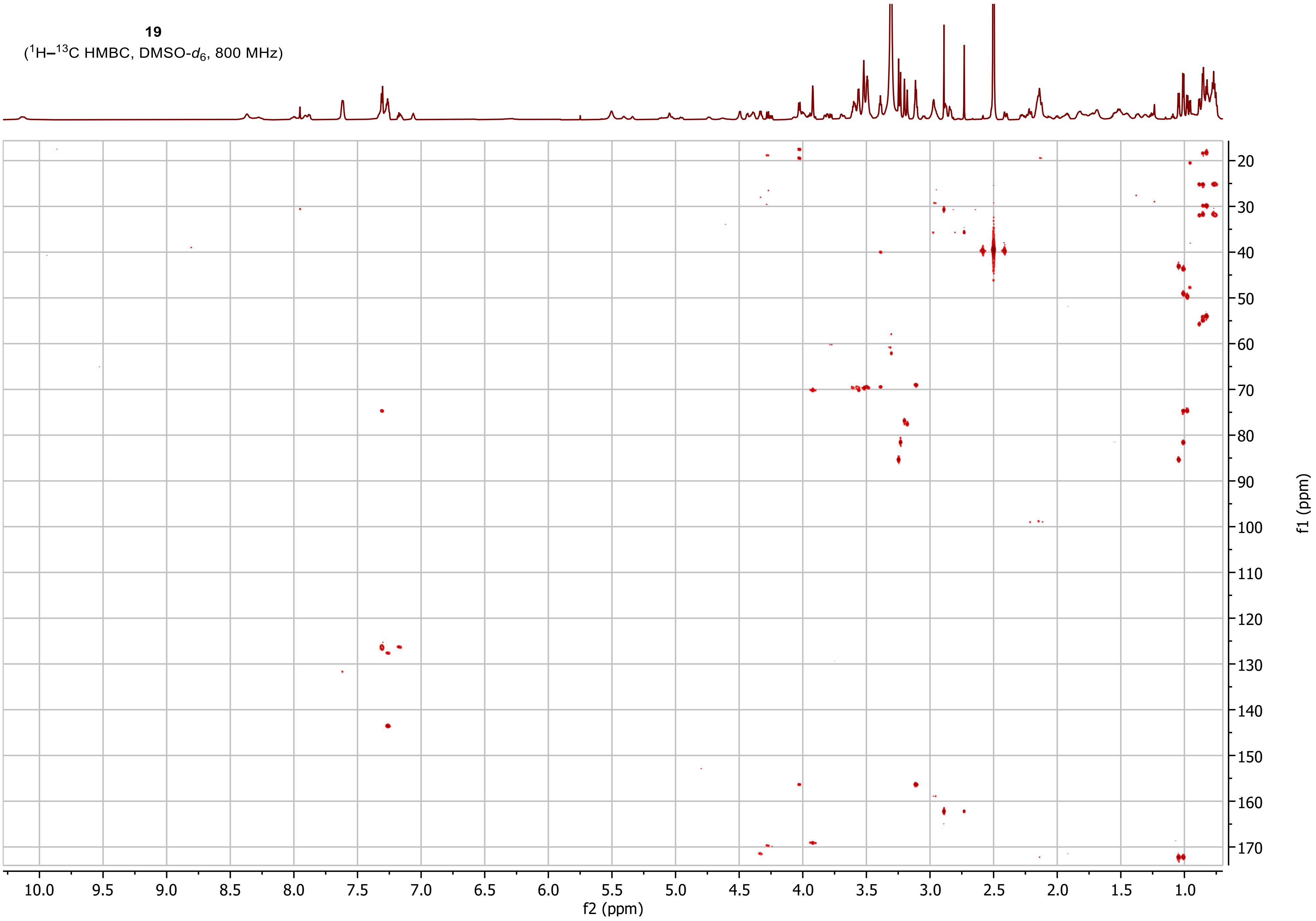

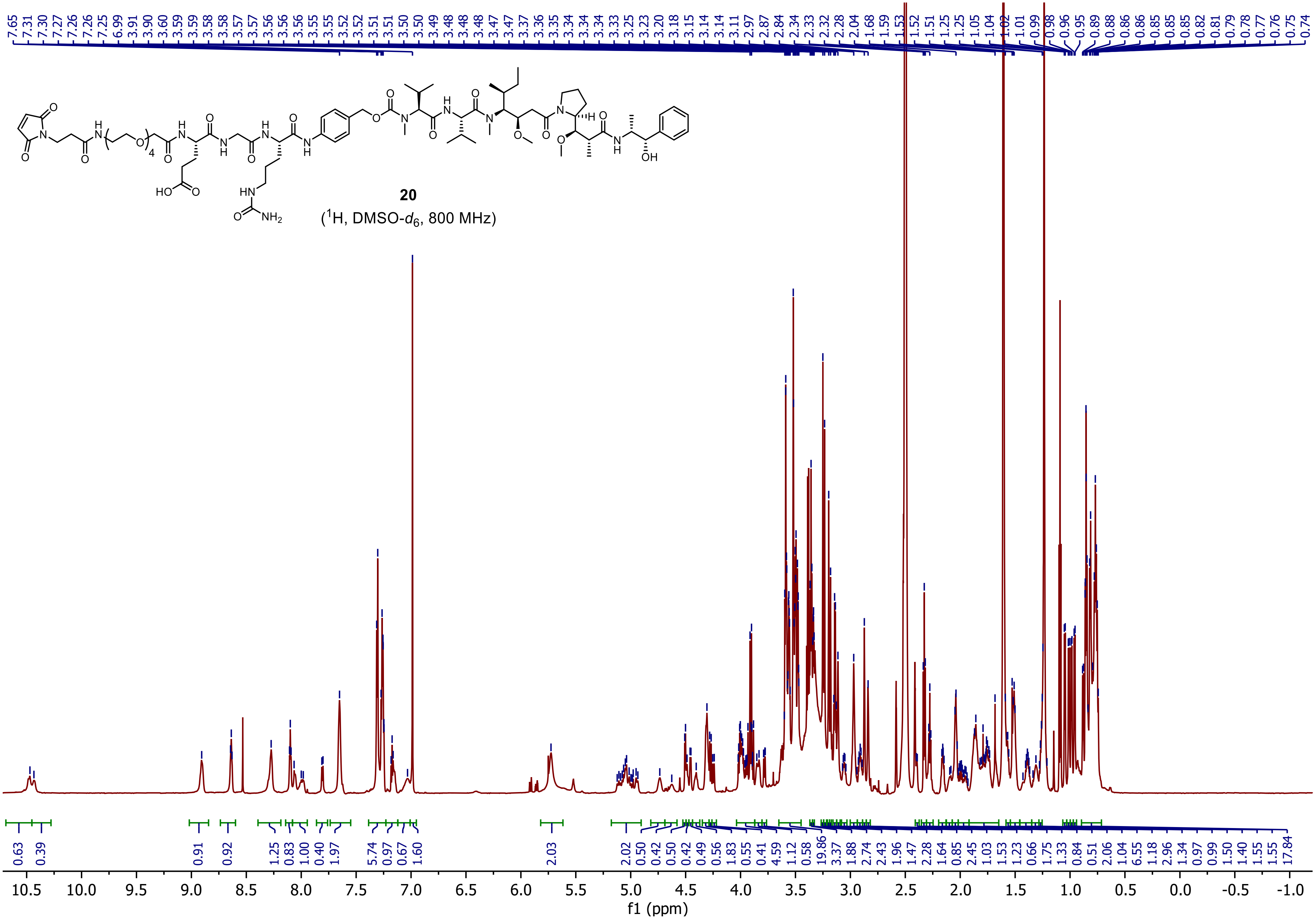
20
(1H, DMSO-d6, 800 MHz)
f1 (ppm)

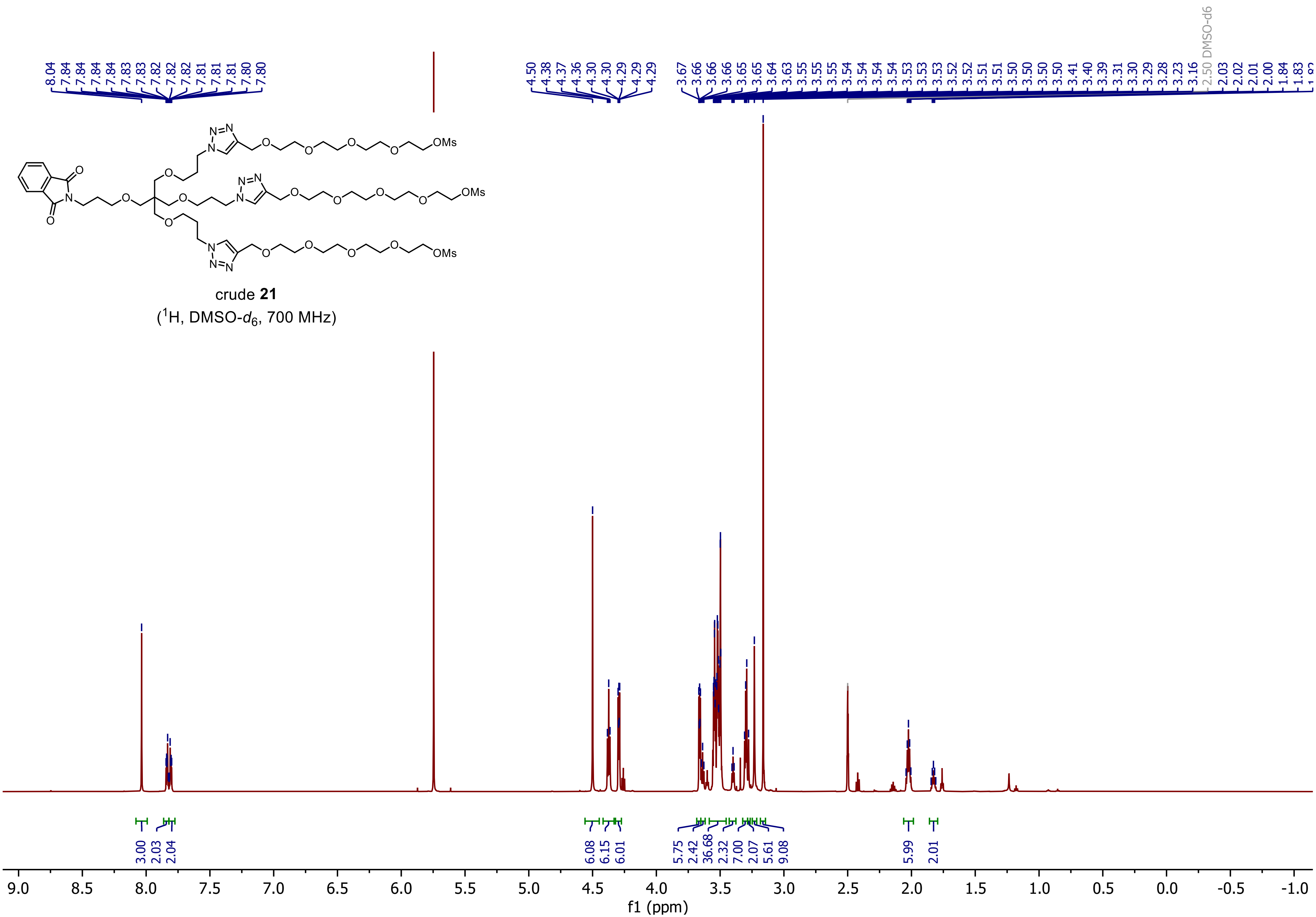

crude **21**

(^{1}H, DMSO-d_6, 700 MHz)

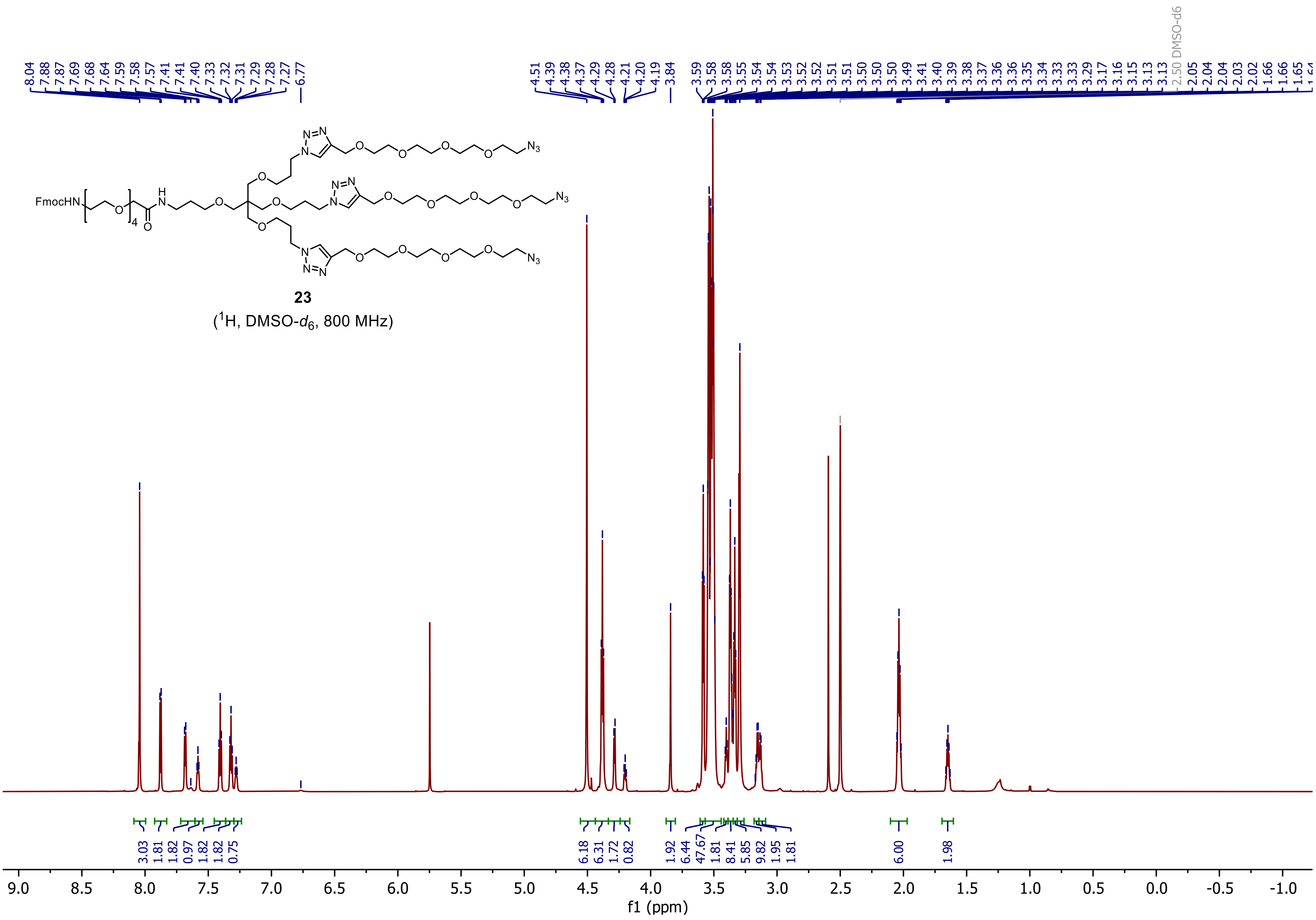

23

(^{1}H, DMSO-d_6, 800 MHz)

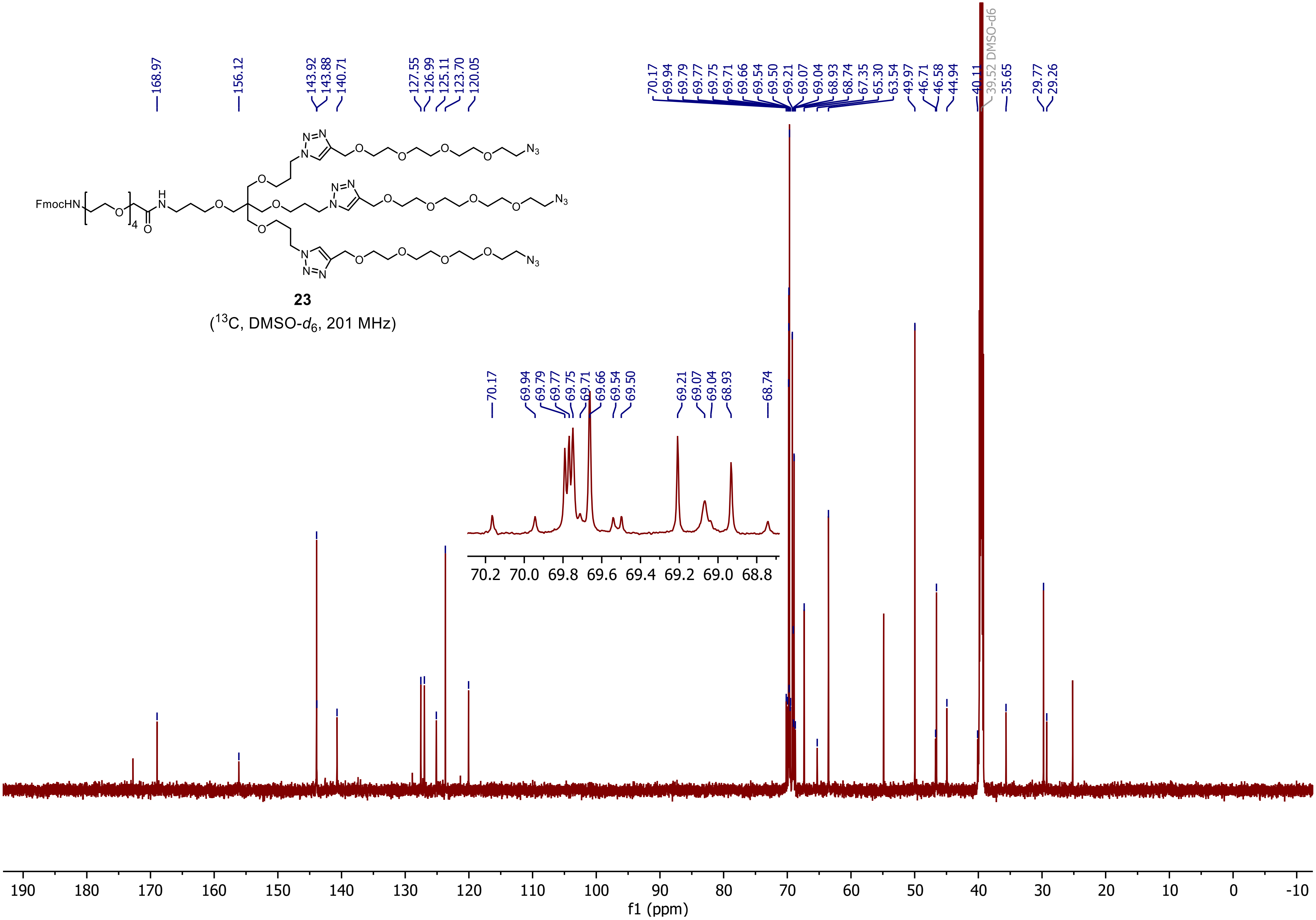
168.97
156.12
143.92
143.88
140.71
127.55
126.99
125.11
123.70
120.05
70.17
69.94
69.79
69.77
69.75
69.71
69.66
69.54
69.50
69.21
69.07
69.04
68.93
68.74
67.35
65.30
63.54
49.97
46.71
46.58
44.94
40.11
39.52 DMSO-d6
35.65
29.77
29.26
FmocHN
N3
23
(13C, DMSO-d6, 201 MHz)
f1 (ppm)

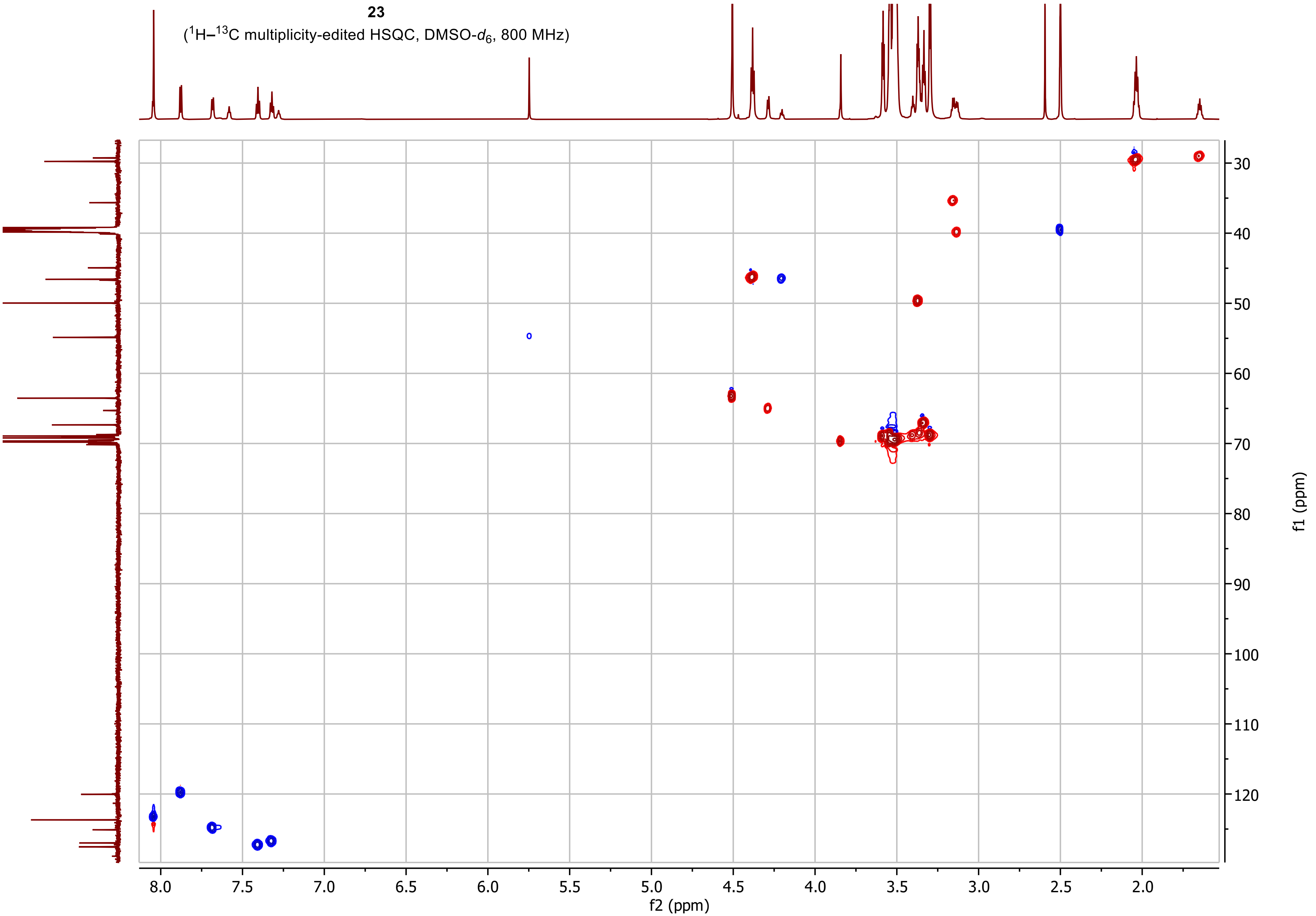

23
(^{1}H–^{13}C multiplicity-edited HSQC, DMSO-d_6, 800 MHz)
f2 (ppm)
f1 (ppm)

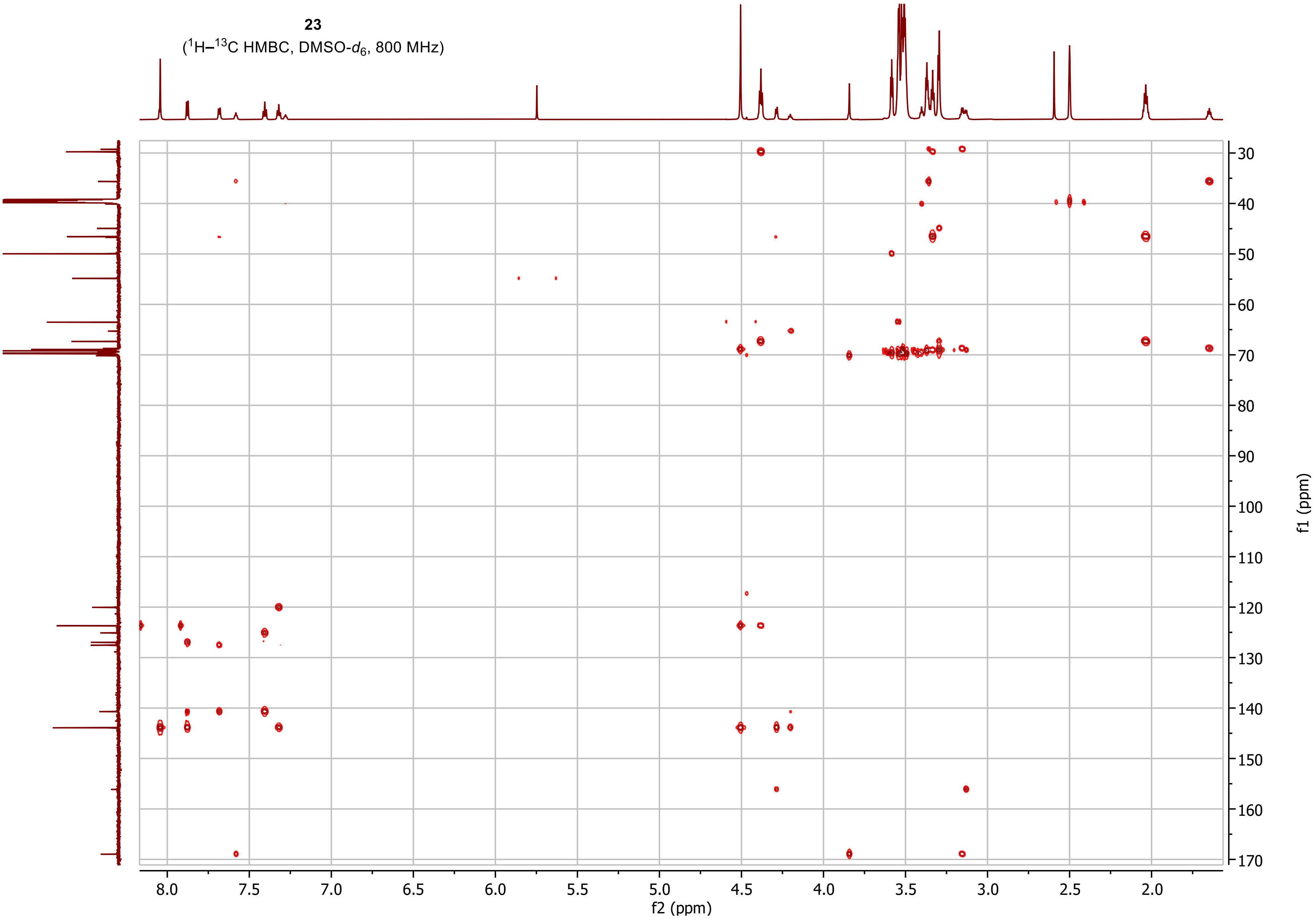
23
(^{1}H–^{13}C HMBC, DMSO-d_6, 800 MHz)
f2 (ppm)
f1 (ppm)

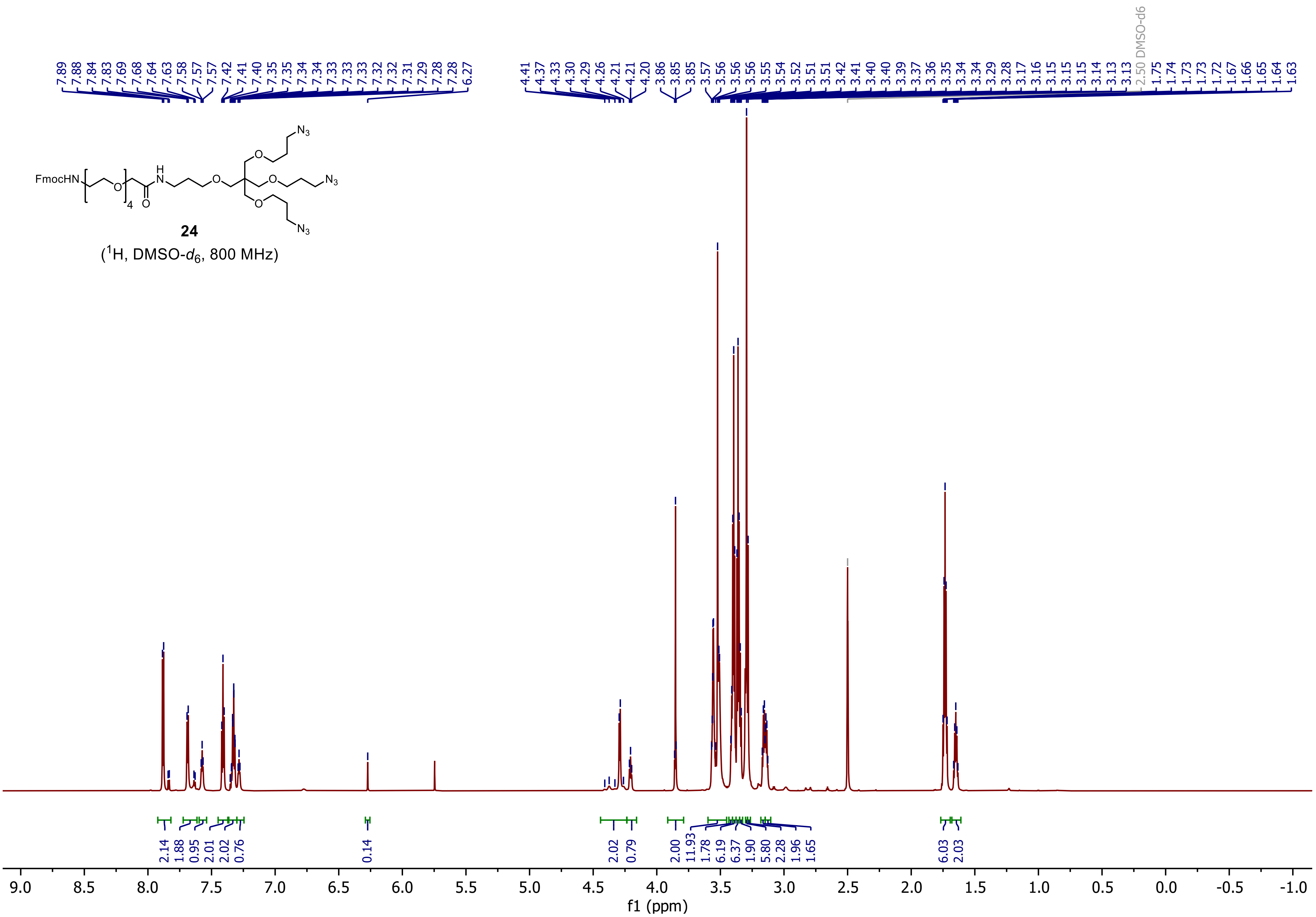

FmocHN
4
24
(¹H, DMSO-d6, 800 MHz)
2.50 DMSO-d6
f1 (ppm)

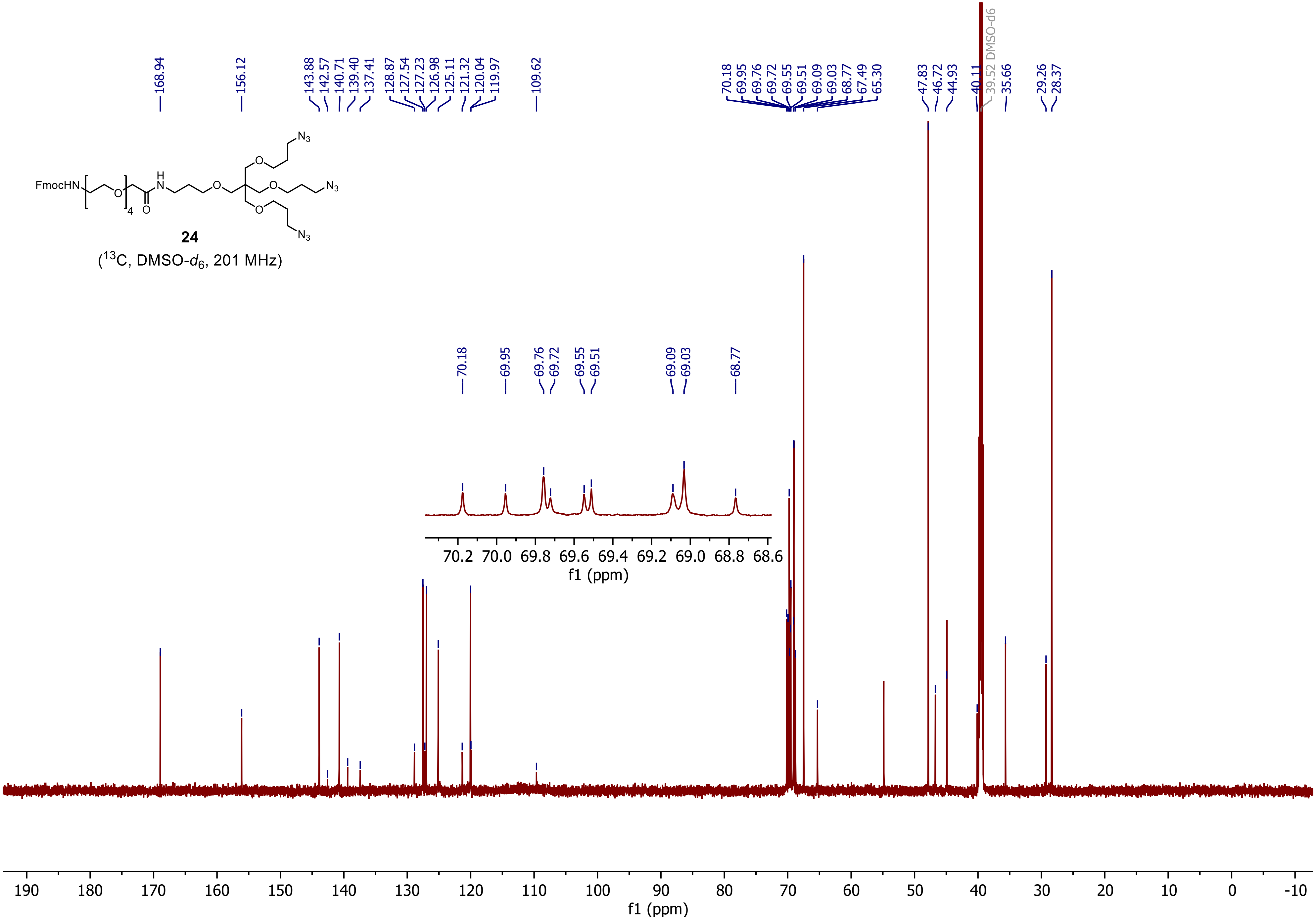
168.94
156.12
143.88
142.57
140.71
139.40
137.41
128.87
127.54
127.23
126.98
125.11
121.32
120.04
119.97
109.62
70.18
69.95
69.76
69.72
69.55
69.51
69.09
69.03
68.77
67.49
65.30
47.83
46.72
44.93
40.11
39.52 DMSO-d6
35.66
29.26
28.37
FmocHN
O
4
H
N
O
O
O
N3
O
N3
O
N3
24
(13C, DMSO-d6, 201 MHz)
f1 (ppm)
f1 (ppm)

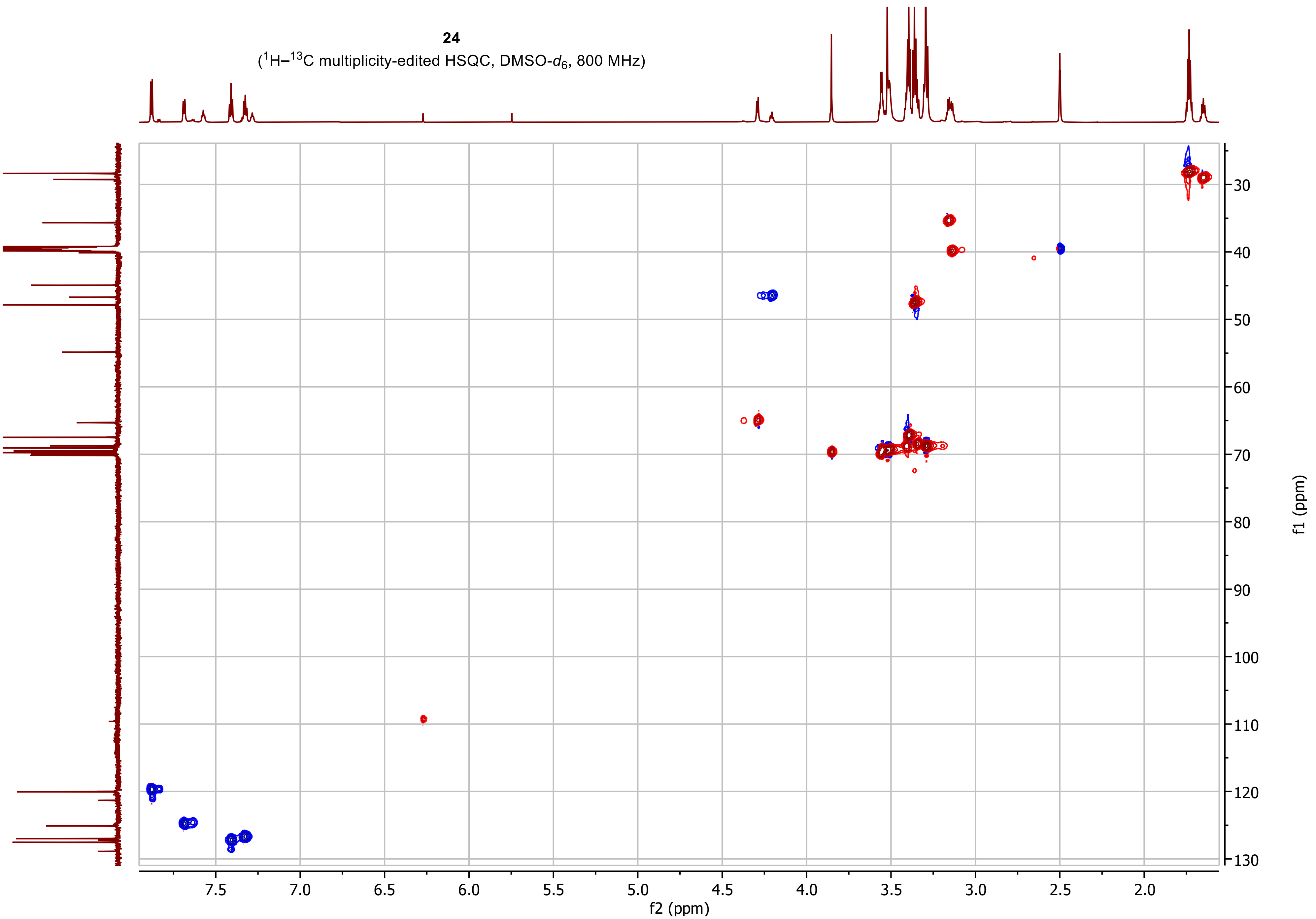
24
(¹H–¹³C multiplicity-edited HSQC, DMSO-d6, 800 MHz)
f2 (ppm)
f1 (ppm)

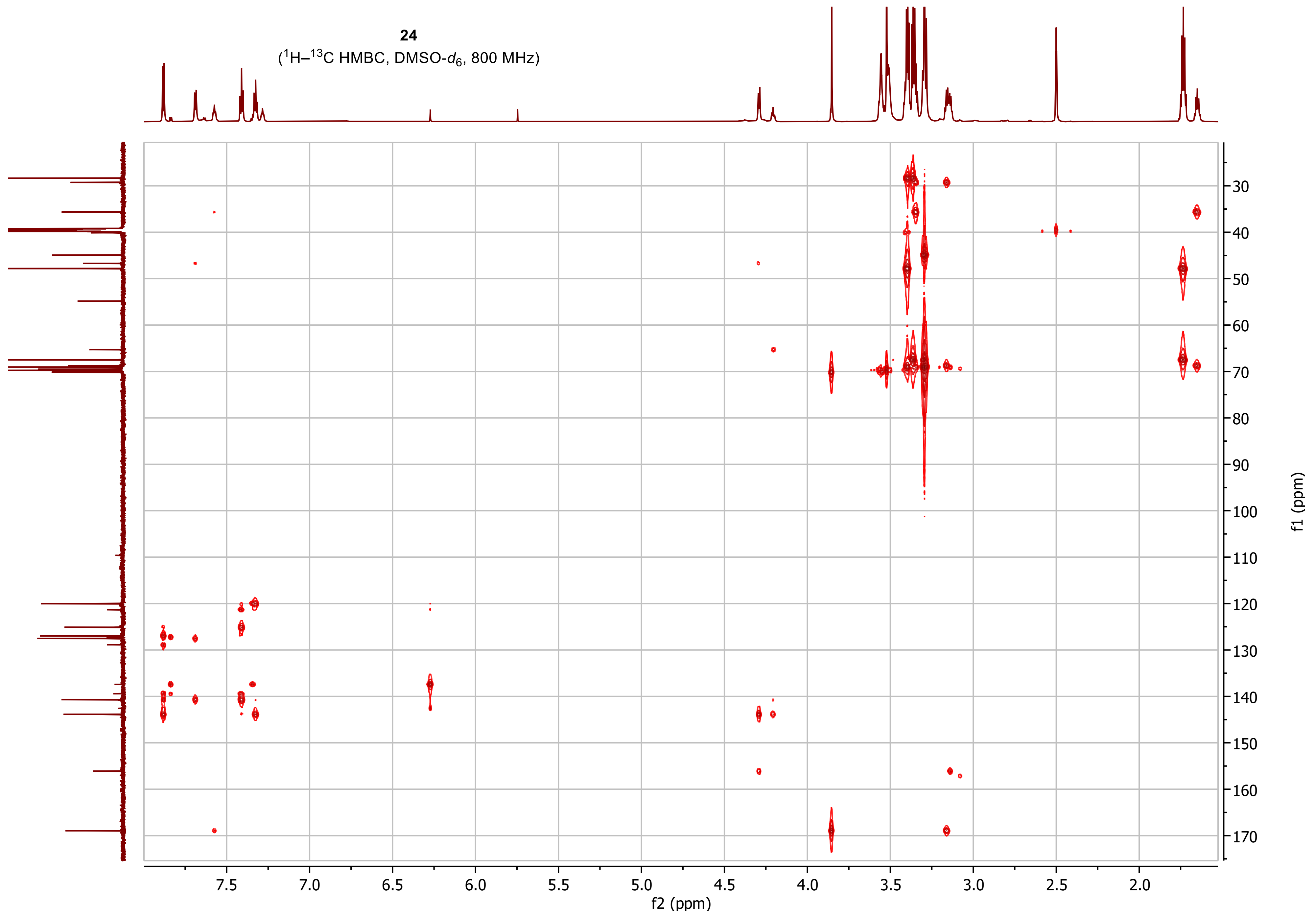
24
(¹H–¹³C HMBC, DMSO-d6, 800 MHz)
f2 (ppm)
f1 (ppm)

HRMS spectra

2, $[M+2H]^{2+}$

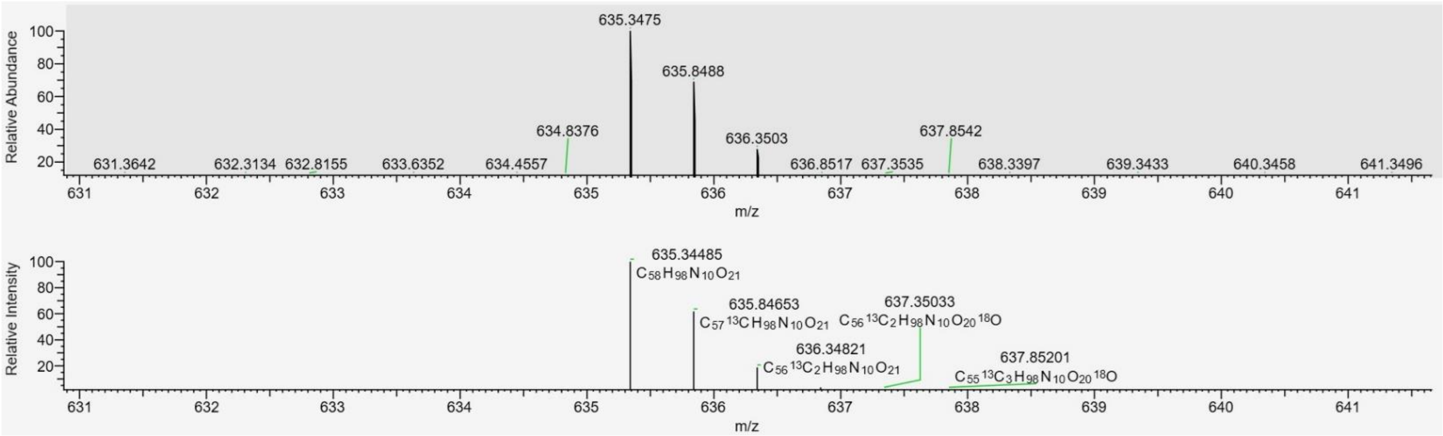

3, $[M+2H]^{2+}$

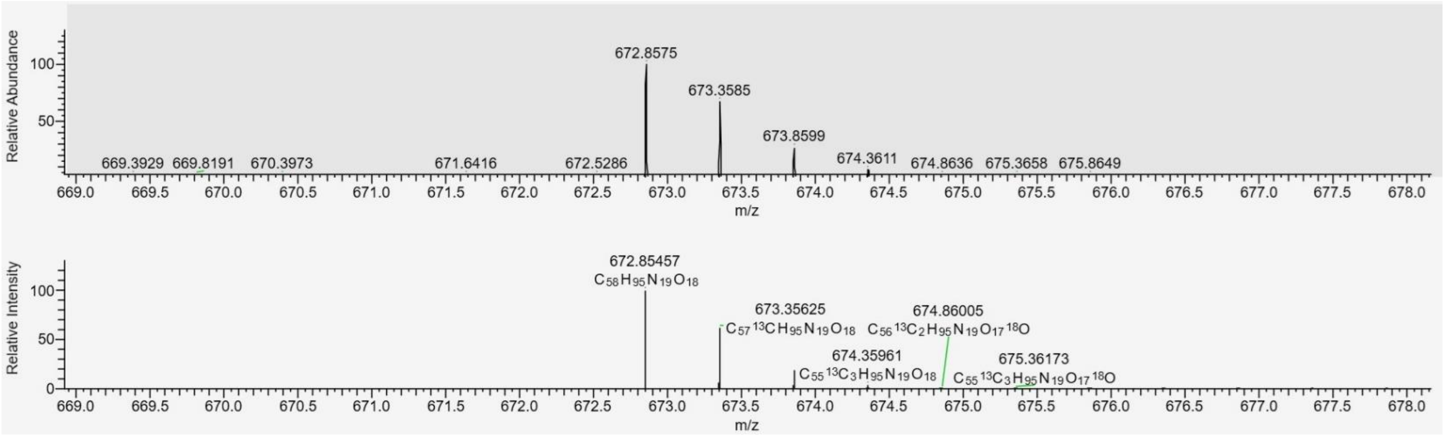

4, $[M+2H]^{2+}$

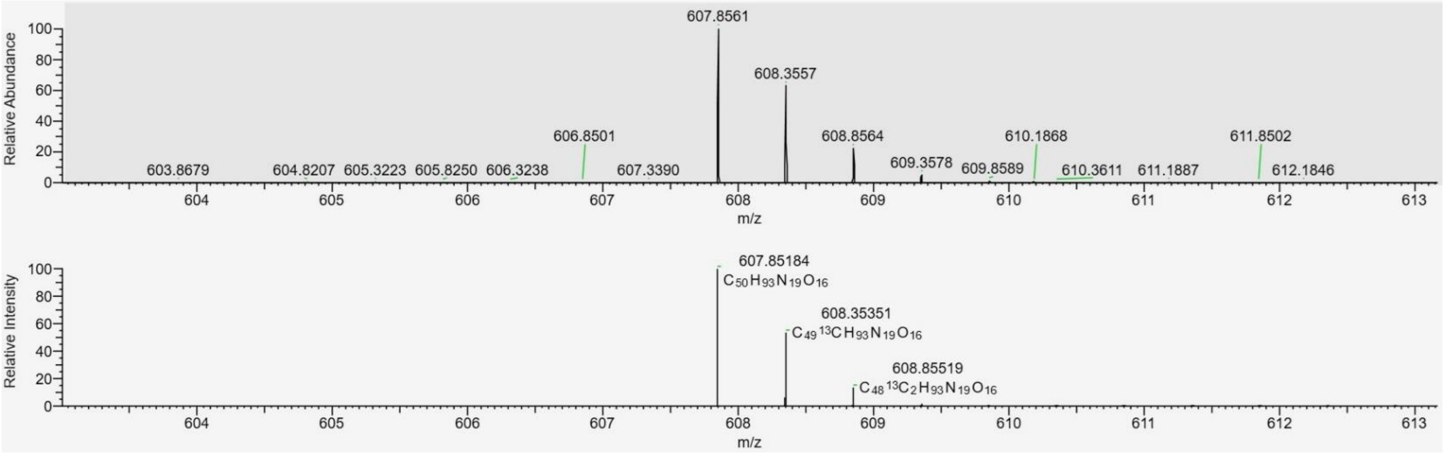

6, $[M+NH_4]^+$

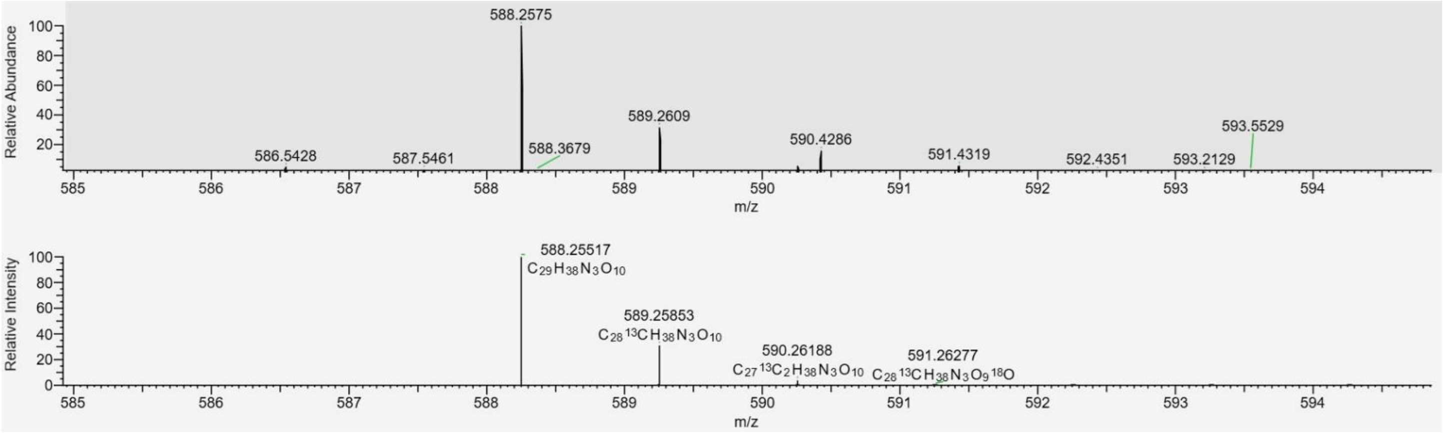

7, $[M+2H]^{2+}$

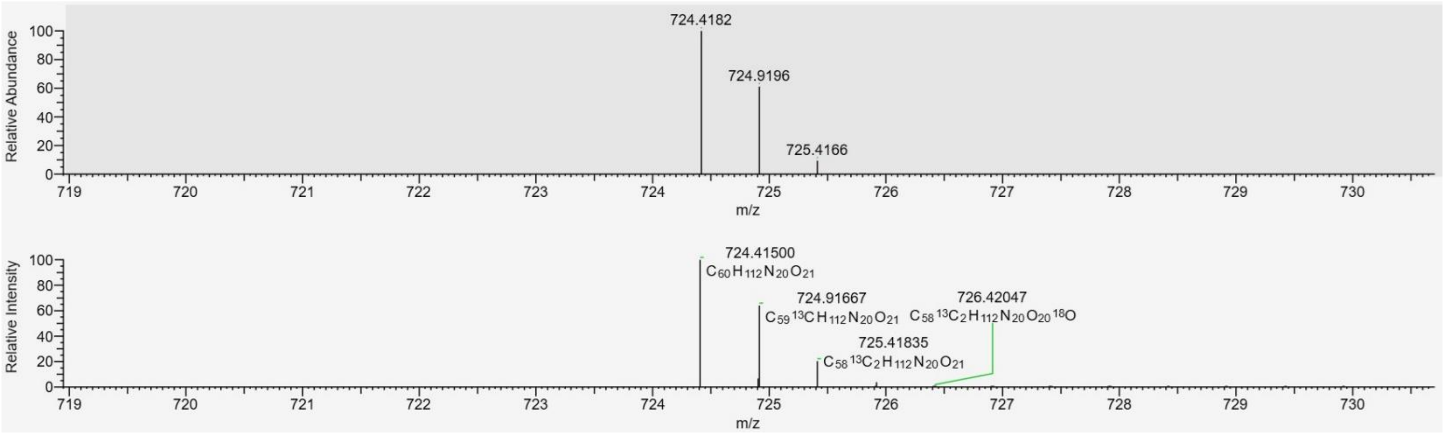

9, $[M+H]^+$

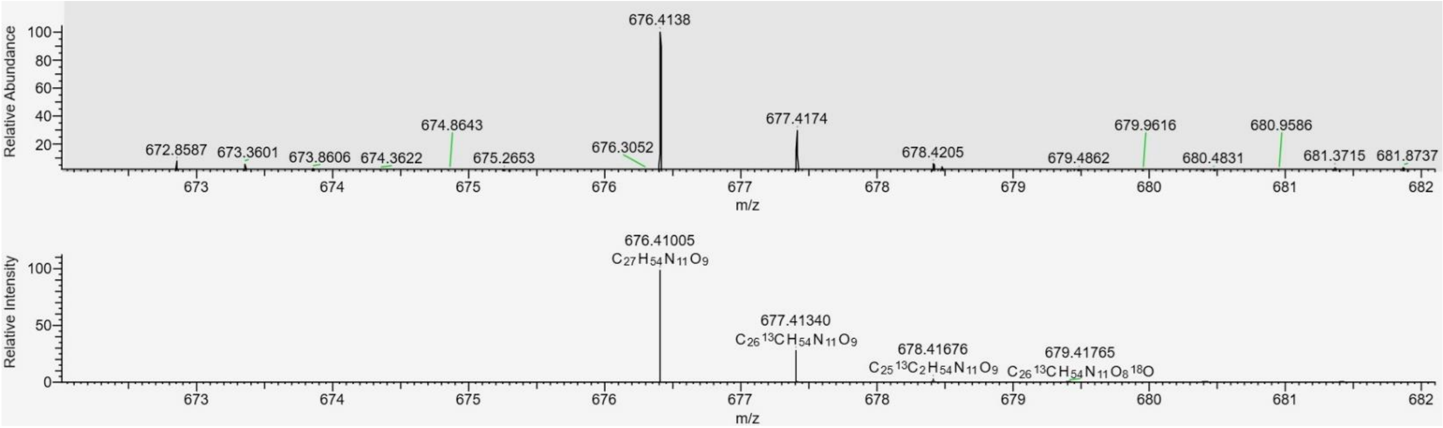

11, [M+Na]+

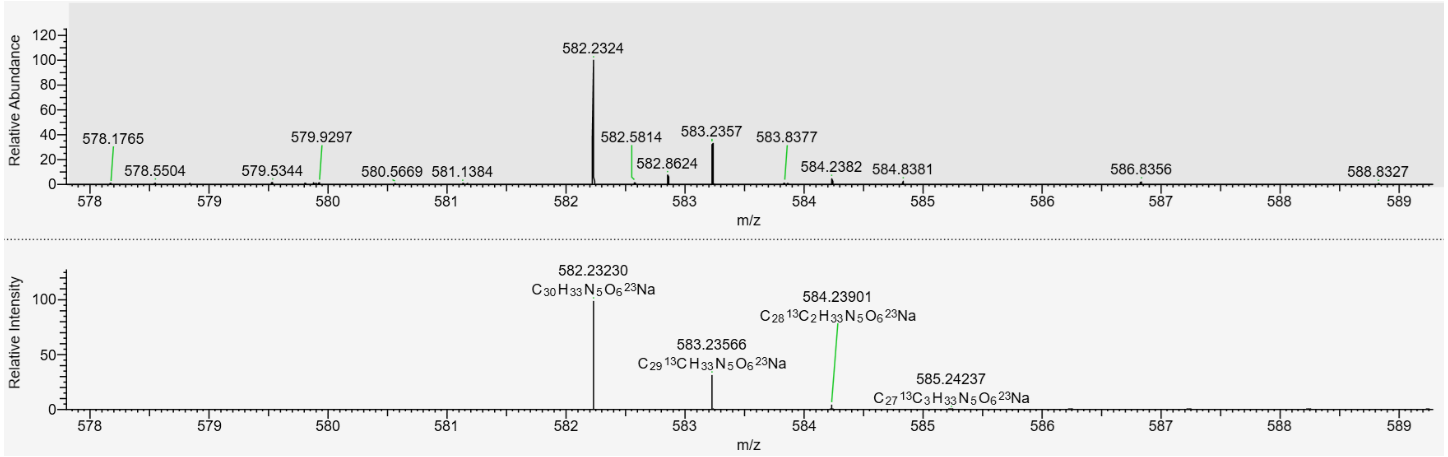

12, [M+H]+

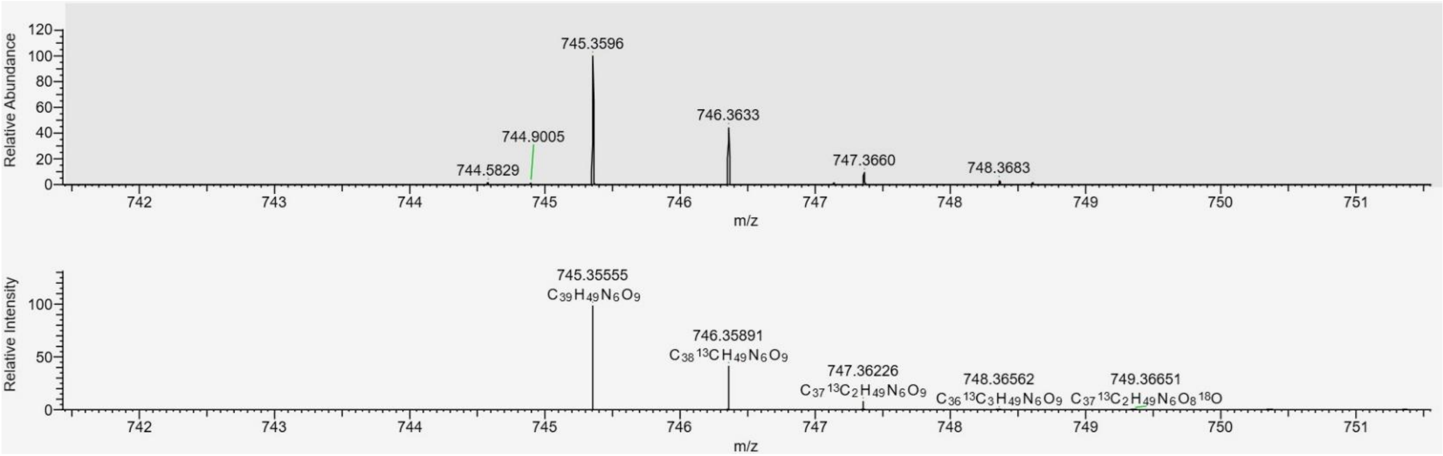

13, [M+H]+

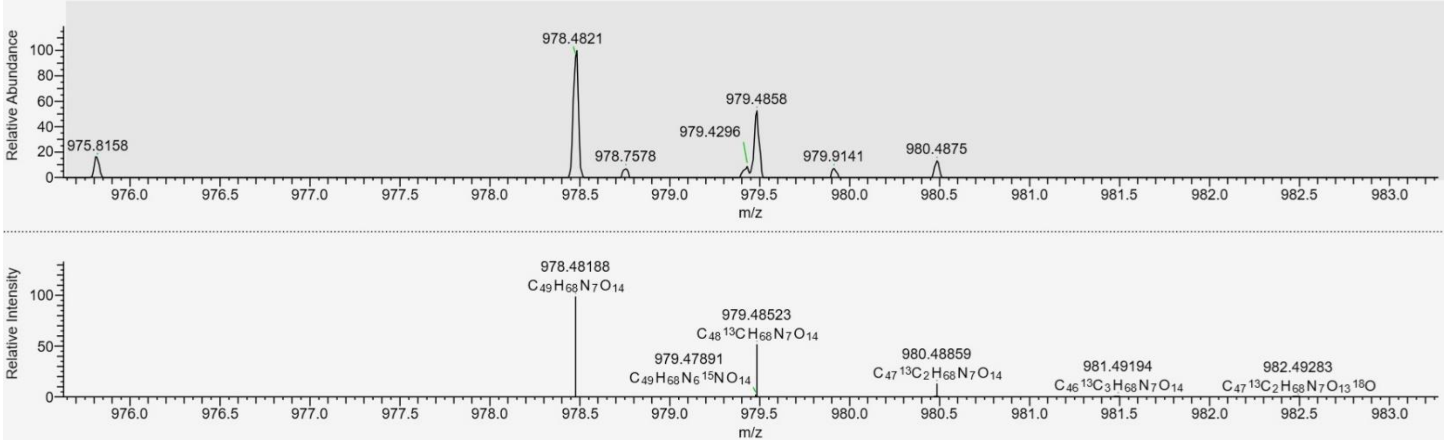

14, $[M+H]^+$

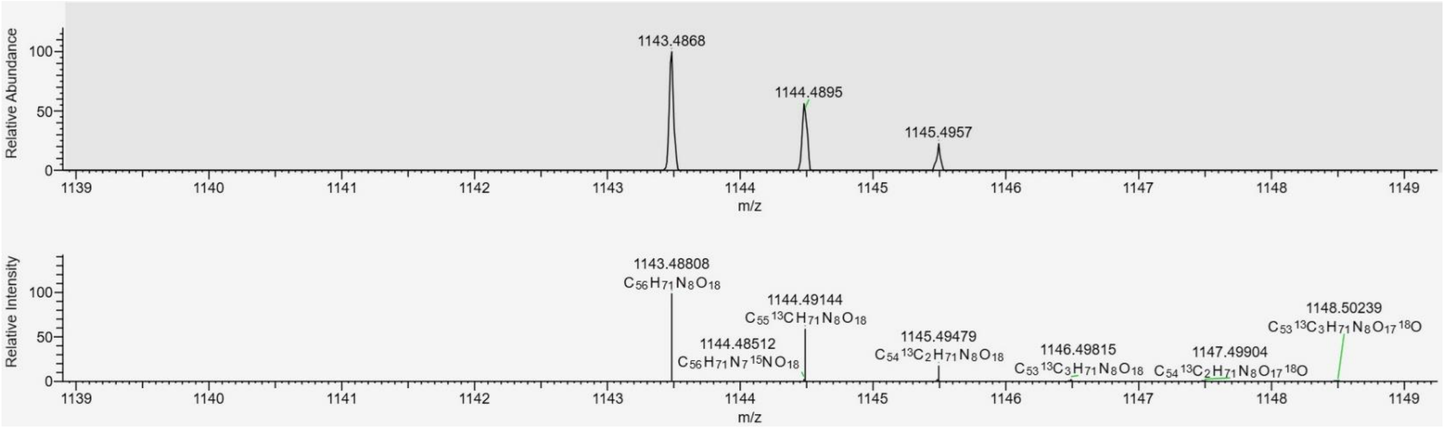

15, $[M+2H]^{2+}$

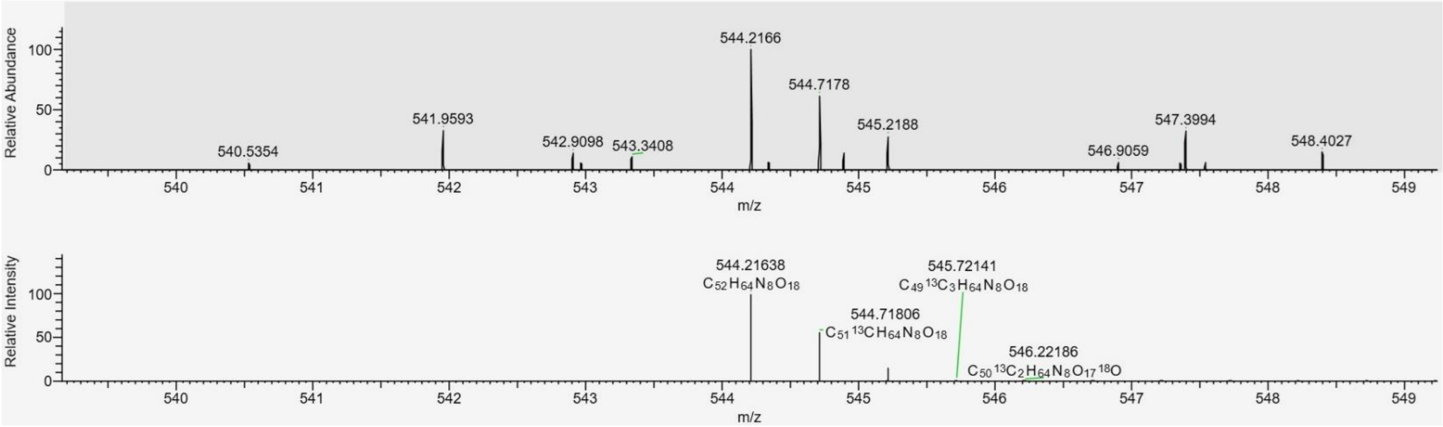

19, $[M-H]^-$

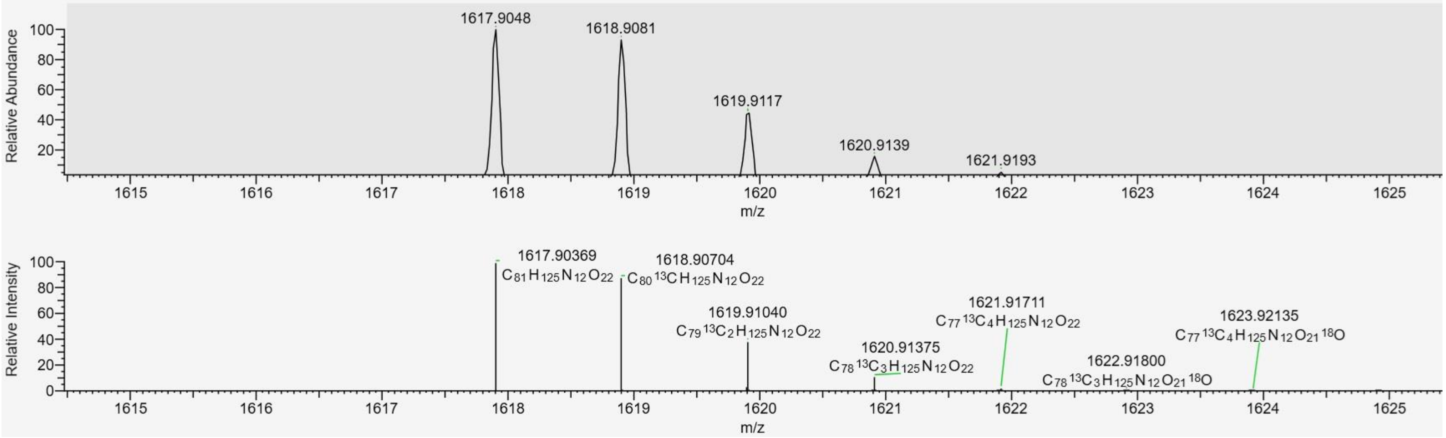

20, $[M+2H]^{2+}$

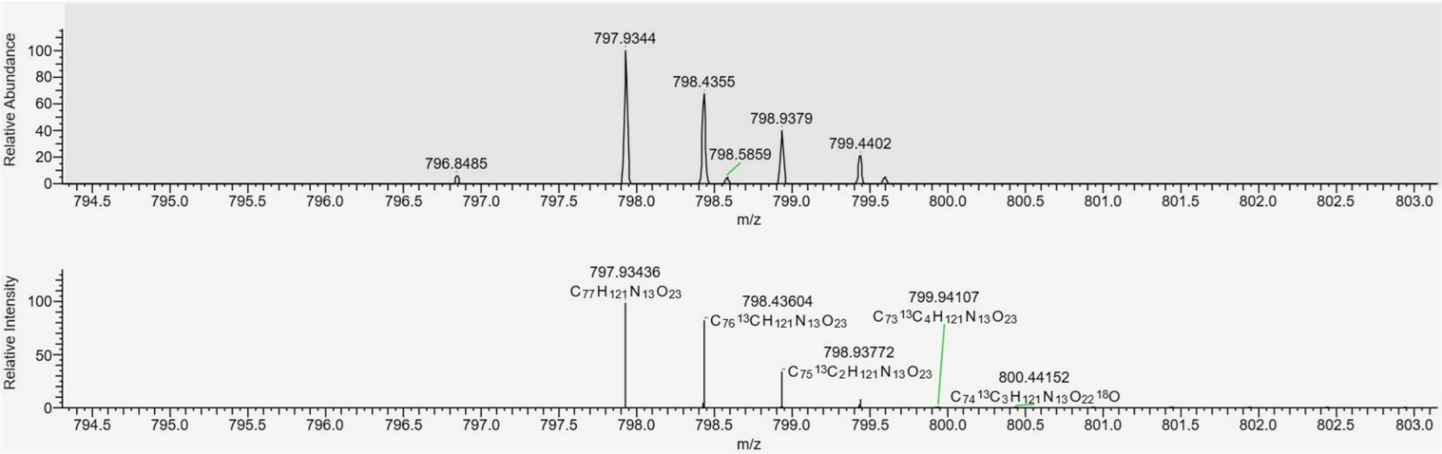

23, $[M+2H]^{2+}$

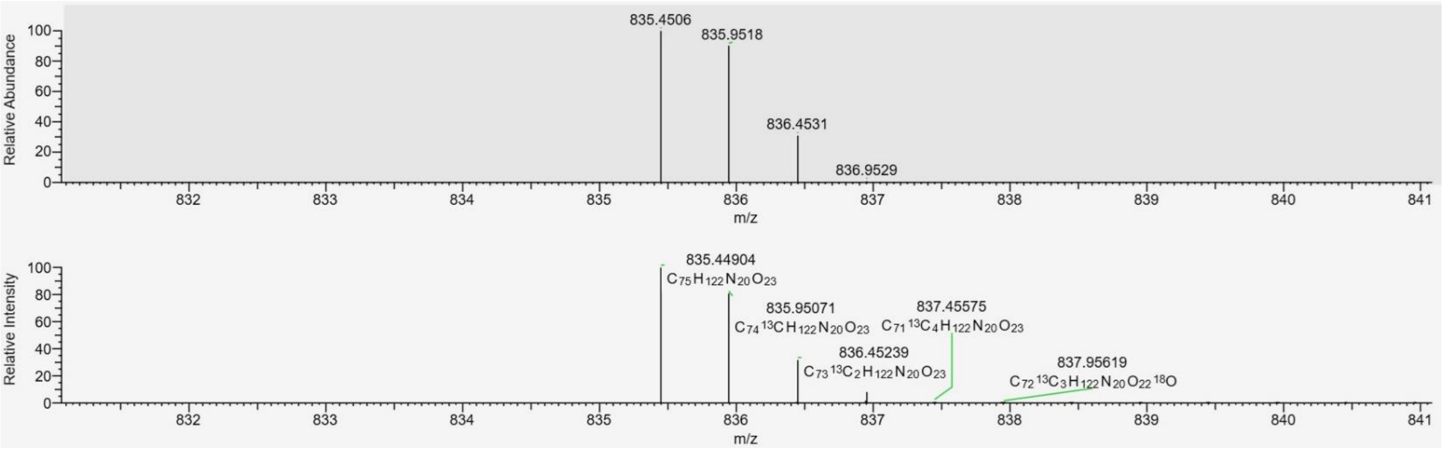

24, $[M+H]^{+}$

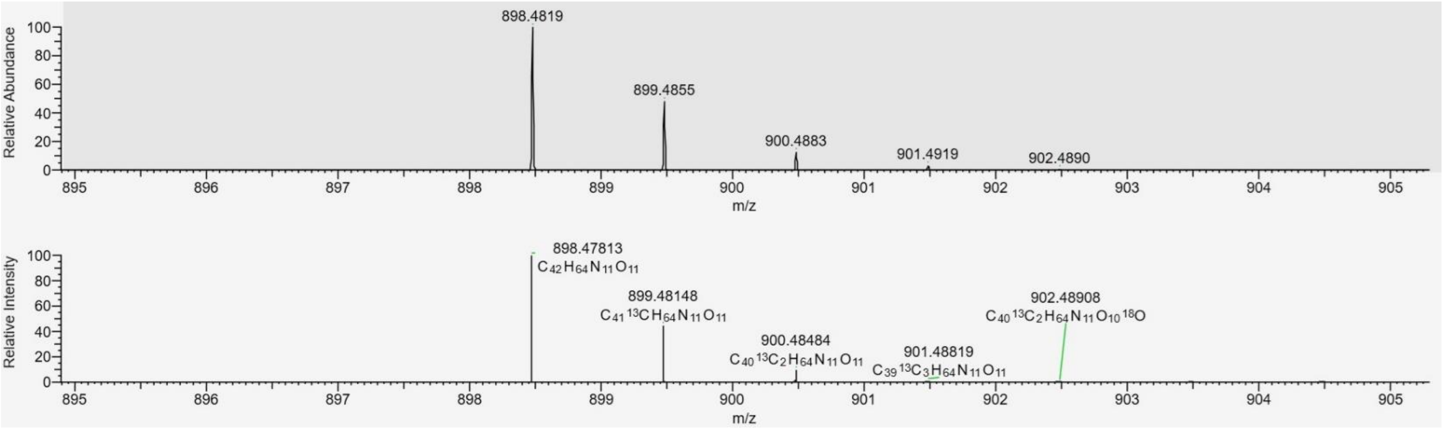

Supplement: Supplementary file 1 [file ijms-25-13356-s001.zip › ijms-3345762-supplementary.pdf]
